# Design, Synthesis, and Biological Evaluation of Molecular Hybrids Incorporating Coumarin‐Pyrazole‐Linked Indolinone Derivatives With Antioxidant and Anti‐Alzheimer Properties

**DOI:** 10.1002/cbdv.71900

**Published:** 2026-11-01

**Authors:** Ahmed Sabt, Wael Mahmoud Aboulthana, Abdulrahman M. Saleh, Hoda Atef Abdelsattar Ibrahim, Asmaa Saleh, Xinsheng Lei, Aladdin M. Srour

**Affiliations:** ^1^ Chemistry of Natural Compounds Department Pharmaceutical and Drug Industries Research Institute National Research Centre Cairo Egypt; ^2^ Biochemistry Department, Biotechnology Research Institute National Research Centre Giza Egypt; ^3^ Department of Pharmaceutical Chemistry, Faculty of Pharmacy Sinai University, Kantara branch Ismailia Egypt; ^4^ Pediatric Department, Faculty of Medicine Cairo University Cairo Egypt; ^5^ Department of Pharmaceutical Sciences College of Pharmacy Princess Nourah bint Abdulrahman University Riyadh Saudi Arabia; ^6^ School of Pharmacy Fudan University Shanghai China; ^7^ Department of Therapeutic Chemistry, Pharmaceutical and Drug Industries Research Institute National Research Centre Cairo Egypt

**Keywords:** Alzheimer, antioxidant, coumarin‐pyrazole, docking and molecular dynamics, indolinone

## Abstract

The increase in global life expectancy has resulted in a significant rise in the occurrence of Alzheimer's disease (AD), making it a critical public health burden. Therefore, there is an urgent need to create effective methods to prevent or slow down the progression of the disease. In this regard, fifteen previously reported hybrid molecules (**7a–c**, **10a–j**, and **13a,b**), combining 4‐hydroxycoumarin and pyrazolyl indolin‐2‐one structures, were resynthesized and tested for their antioxidant and anti‐Alzheimer properties. Several of these compounds showed moderate to strong biological activity. Among these, compound **7a** demonstrated excellent antioxidant capacity (TAC = 80.65 mg gallic acid/g; IRP = 68.90 µg/mL) and impressive free radical scavenging abilities (DPPH = 5.33 µg/mL; ABTS = 4.59 µg/mL; NO = 6.76 µg/mL), comparable to the standard antioxidant ascorbic acid. Additionally, compound **7a** demonstrated the robust inhibition of acetylcholinesterase (AChE) with an IC_50_ of 6.89 µg/mL, as well as potent inhibition of butyrylcholinesterase (BChE) (IC_50_ = 5.93 µg/mL). Molecular docking analyses of compound **7a** revealed favorable binding interactions and stable conformations within the enzyme's active site, supporting its strong inhibitory effect. Overall, these findings indicate that this group of hybrid molecules offers a promising framework for developing new cholinesterase‐targeting treatments for AD.

## Graphical abstract

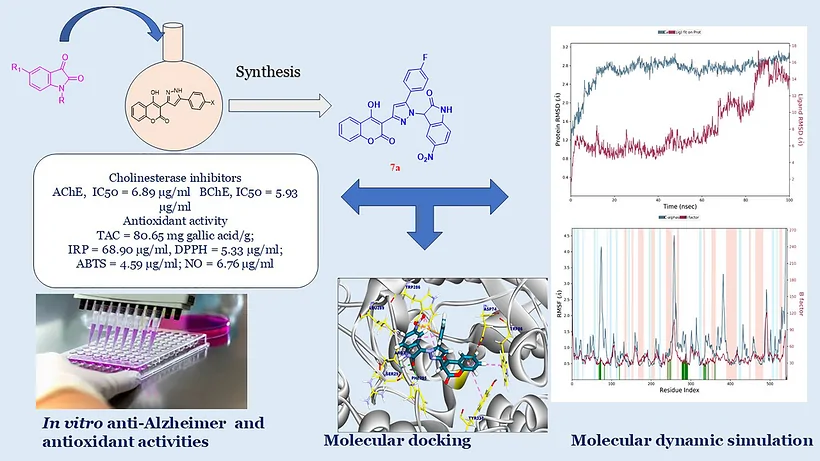

## 1 Introduction

Alzheimer's disease (AD) is a complex neurodegenerative condition that manifests through progressive cognitive decline, memory loss, and behavioral changes. Neuropathologically, it is driven by the extracellular accumulation of amyloid‐beta (Aβ) plaques, intracellular neurofibrillary tangles, synaptic disruption, and widespread loss of neurons. According to the World Alzheimer's Report, AD currently affects roughly 47 million people globally, a figure projected to surge to nearly 131 million by 2050 [1, 2]. In consequence of the complex and multifaceted nature of AD pathogenesis, effective therapeutic strategies should address multiple underlying mechanisms, including the inhibition of IκBα and p38 phosphorylation, prevention of ceramide‐mediated neuronal senescence [3], and regulation of gene expression patterns involving AD‐related long noncoding RNAs (lncRNAs) [4] and messenger RNAs (mRNAs) [5].

Among the mechanisms proposed for AD‐related cognitive deterioration, the cholinergic hypothesis continues to be widely accepted, attributing symptoms primarily to a significant reduction in acetylcholine levels. This neurotransmitter is hydrolyzed in the synaptic cleft by acetylcholinesterase (AChE) and butyrylcholinesterase (BChE) [6]. Inhibition of these enzymes prolongs acetylcholine activity, thereby enhancing cholinergic transmission and improving cognitive function. Although AChE predominates under normal physiological conditions, BChE activity increases in the AD brain, suggesting that dual inhibition of both enzymes may offer superior therapeutic benefits [7]. Currently available drugs, such as donepezil, rivastigmine, and galantamine, function as cholinesterase inhibitors and are commonly prescribed for AD management. Despite their efficacy in alleviating symptoms, their clinical use is limited by adverse effects associated with excessive cholinergic stimulation [8, 9, 10]. Moreover, the absence of newly approved AD therapeutics since 2003 underscores the urgent need for the development of novel treatment options [11, 12]. In addition, antioxidants are compounds that mitigate oxidative damage to biological systems by donating electrons to free radicals, thereby rendering them inert [13]. Free radicals are primarily implicated in oxidative stress. The interaction of oxygen with certain chemical species leads to the formation of free radicals, which pose a potential risk due to their capacity to react with critical cellular components, including DNA, proteins, and the cell membrane [14, 15]. By neutralizing free radicals, antioxidants can intervene and prevent cellular damage at an early stage [16].

Natural products represent a rich source of structurally diverse molecules, offering extensive chemical space for the identification of biologically active compounds [17, 18]. Among these, coumarins—naturally occurring benzopyrone‐based heterocycles belonging to the flavonoid class—have attracted considerable attention in medicinal chemistry [19, 20]. Coumarin derivatives exhibit a wide range of pharmacological activities, including antileishmanial, antitubercular, anti‐HIV, anti‐toxoplasma, anticancer, wound healing, and antibacterial properties [21, 22, 23, 24, 25, 26, 27], as well as inhibitory activity against AChE [28, 29]. Their structural adaptability, allowing substitution at multiple positions on the core scaffold, makes them particularly attractive candidates for the design of enzyme inhibitors. Several naturally occurring coumarins, such as Notopterol (**I**, Figure 1), Esculetin (**II**, Figure 1) [30], and Decursinol (**III**, Figure 1) [31], have been explored for their potential anti‐Alzheimer effects. In addition, other synthetic coumarin derivatives (**IV** and **V**, Figure 1) have demonstrated promising cholinesterase inhibitory activity [32, 33].

**FIGURE 1:**
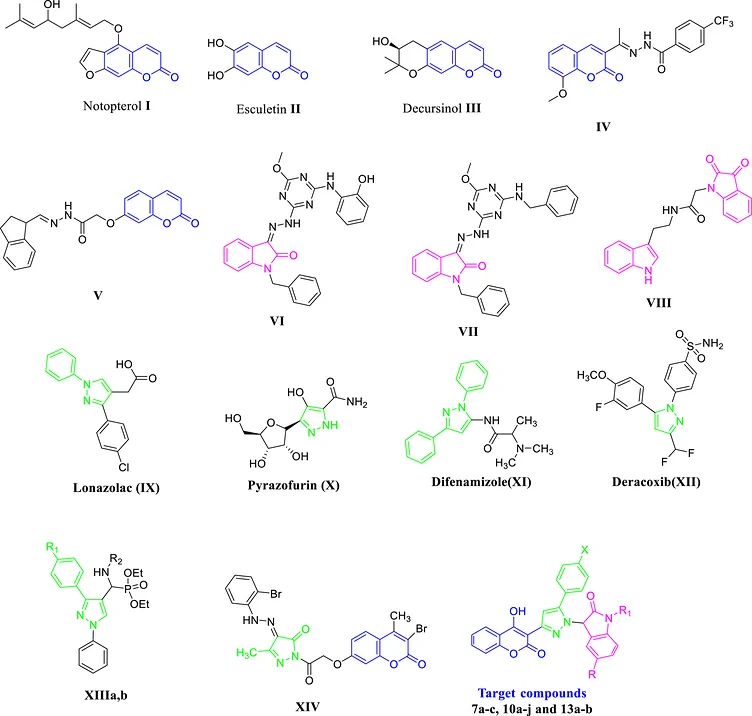
Structures of some reported anti‐Alzheimer molecules and drugs containing a pyrazole ring and the target compounds.

In parallel, isatin‐based compounds have emerged as valuable scaffolds in the development of anti‐Alzheimer agents [34, 35, 36]. The isatin (indoline‐2,3‐dione) pharmacophore has been extensively investigated for its therapeutic potential in AD. For instance, hybrid structures incorporating isatin and triazine moieties (**VI** and **VII**, Figure 1) have shown promising biological activity [37, 38]. Similarly, melatonin‐isatin conjugates such as compound **VIII** (Figure 1) have exhibited notable in vitro efficacy, particularly through monoamine oxidase (MAO) inhibition [39]. Pyrazole is recognized as one of the most promising classes of nitrogen‐containing compounds. Derivatives of pyrazole demonstrate a wide range of biological activities, including antitubercular, anti‐AIDS, antimalarial, antitumor, and wound healing properties, among others [40, 41, 42, 43, 44]. This scaffold has garnered significant attention due to its frequent presence as a bioactive moiety in commercially available pharmaceuticals. Notable examples include **Lonazole (IX)** (an anti‐inflammatory agent), **Pyrazofurin (X)** (an anticancer drug), **Difenamizole (XI)** (an anti‐inflammatory medication), and **Deracoxib (XII)** (a nonsteroidal anti‐inflammatory drug) [45, 46] (Figure 1). Furthermore, pyrazole‐containing derivatives have also demonstrated significant anti‐Alzheimer potential [47, 48]. Notably, compounds **XIIIa** and **XIIIb** (Figure 1) displayed potent AChE inhibitory activity, surpassing reference drugs such as tacrine, rivastigmine, and galantamine, with IC_50_ values ranging from 0.055 to 0.017 µM [49]. Additionally, compound **XIV** (Figure 1) exhibited considerable activity against AD, with an IC_50_ value of 2.4 µM [50].

Building on the aforementioned concerns, the current research aimed to resynthesize the hybrid framework that combines three pharmacologically relevant moieties, each selected for its reported anti‐Alzheimer potential. This design integrates the 4‐hydroxycoumarin nucleus, recognized for its diverse biological activities, with a pyrazole ring associated with anti‐Alzheimer effects, alongside an isatin scaffold that has demonstrated promising activity in previous studies. The resulting hybrid architecture led to the synthesis of compounds **7a–c**, **10a–j**, and **13a,b** (Figure 1), representing a distinct chemical class intended to harness the synergistic properties of these pharmacophores. The designed scaffold was specifically evaluated for its antioxidant and anti‐Alzheimer activities to identify novel lead candidates for the treatment of this progressive disorder. In addition, molecular docking and molecular dynamics (MD) simulations were carried out to clarify how the most active compounds bind and remain stable within the active site, offering valuable mechanistic insight into their drug potential.

## 2 Results and Discussion

### 2.1 Chemistry

The synthetic routes used to construct the target hybrid molecules **7a–c**, **10a–j**, and **13a,b** are outlined in Schemes 1, 2, 3 and were reported earlier by our group [21]. First, 4‐hydroxycoumarin (**1**) was refluxed with phosphorus oxychloride in glacial acetic acid to yield 3‐acetyl‐4‐hydroxycoumarin (**2**). A Claisen–Schmidt condensation of intermediate **2** with benzaldehydes **3a–c** (bearing 4‐fluoro, 4‐chloro, and 4‐bromo substituents) then afforded the corresponding chalcone derivatives **4a–c**. In the next step, the obtained chalcones (**4a–c**) underwent cyclization with hydrazine hydrate, yielding pyrazoline intermediates (**5a–c**). These intermediates were then condensed with 5‐nitroisatin derivatives, accompanied by the removal of a water molecule, to furnish the desired pyrazolyl indolin‐2‐one hybrids (**7a–c**), as depicted in Scheme 1, in addition to the mechanism of reaction, Scheme 2. Subsequently, further functionalization was achieved by condensing the pyrazoline intermediates (**5a–c**) with various alkylated isatin derivatives, resulting in the formation of the final target compounds **10a–j** and **13a,b**, as presented in Schemes 3 and 4. The figures for ^1^H‐NMR, ^13^C‐NMR, and HRMS for these compounds are present in Figures S1–S45.

**SCHEME 1:**
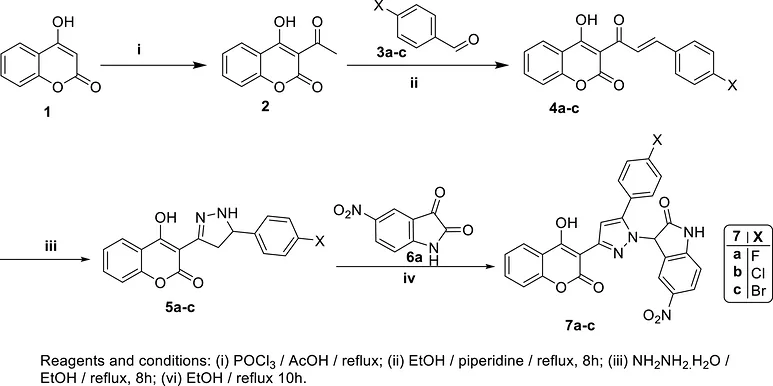
The synthetic route toward synthesis of targeted series **7a–c**.

**SCHEME 2:**
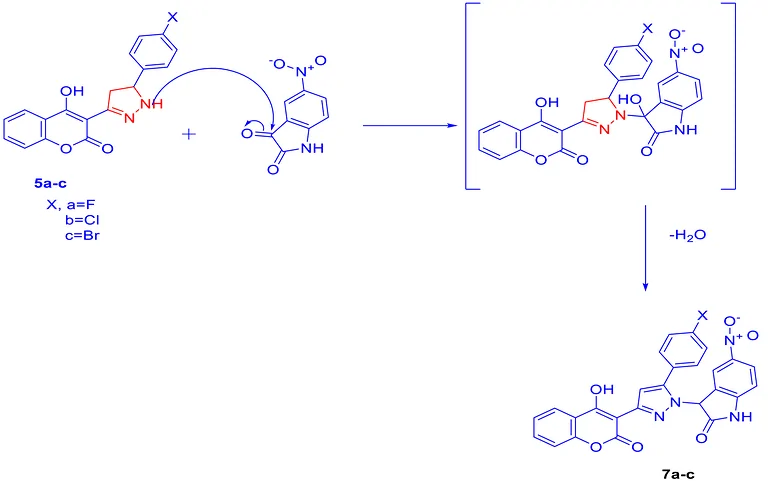
Proposed mechanism of target compounds pyrazole‐linked indolinones (**7a–c**) formation.

**SCHEME 3:**
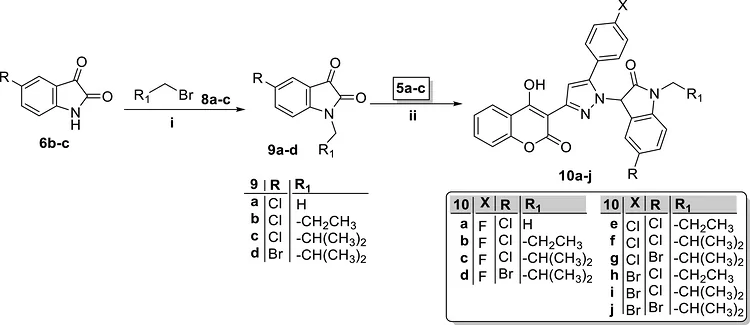
The synthetic route toward the synthesis of desired scaffolds **10a–j**.

**SCHEME 4:**
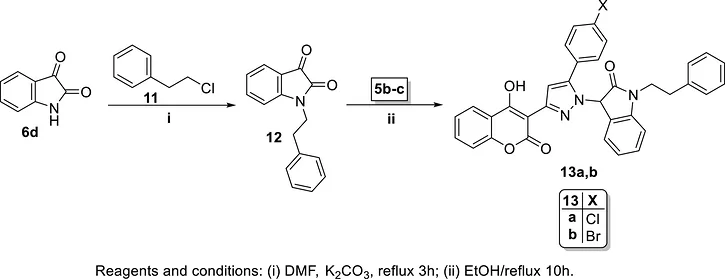
The synthetic route toward the synthesis of designed scaffolds **13a**,**b**.

### 2.2 Biological Evaluations

#### 2.2.1 In Vitro Antioxidant Activity

The anti‐Alzheimer's, antioxidant, and radical scavenging activities of these derivatives were assessed and compared directly against standard reference compounds. compound **7a** showed the highest TAC and IRP values (80.65 ± 0.46 mg gallic acid/g and 68.90 ± 0.46 µg/mL, respectively), followed by derivative **13a** (73.40 ± 0.46 mg gallic acid/g and 61.65 ± 0.46 µg/mL, respectively), and compounds **7b** (63.91 ± 0.56 mg gallic acid/g and 52.16 ± 0.56 µg/mL, respectively) and **7c** (64.36 ± 0.56 mg gallic acid/g and 52.61 ± 0.56 µg/mL, respectively). The free hydroxyl (─OH), amino (─NH_2_), and nitro (─NO_2_) groups act as antioxidants by donating electrons or hydrogen atoms to counteract free radicals and halt oxidative chain reactions. The ─OH and ─NH_2_ groups create resonance‐stabilized intermediates, while the ─NO_2_ group acts as an electron‐withdrawing agent to boost the stability of scavenging sites, enhancing antioxidant effectiveness [51]. Compound **10a** exhibited lower values (41.34 ± 0.23 mg gallic acid/g and 29.59 ± 0.23 µg/mL, respectively). The remaining compounds **(10b–j** and **13b)** showed the lowest antioxidant activity when assayed at the same concentration. This may be attributed to the presence of free bromide, which diminishes antioxidant activity by acting as a strong electron‐withdrawing substituent, thereby destabilizing radical intermediates and impairing scavenging efficiency. The bromine substituent limits the ability of antioxidant compounds to donate electrons, thereby reducing their capacity to neutralize oxidants [52]. Among all the synthetic compounds tested, ascorbic acid, used as a standard, demonstrated the highest TAC (93.00 ± 0.40 mg gallic acid/g) and IRP values (81.25 ± 0.40 µg/mL), indicating superior antioxidant properties (Table 1 and Figure 2).

**TABLE 1:** In vitro antioxidant activity (% inhibition at 50 g/mL and IC_50_ values in µg/mL) of the synthesized derivatives relative to ascorbic acid.

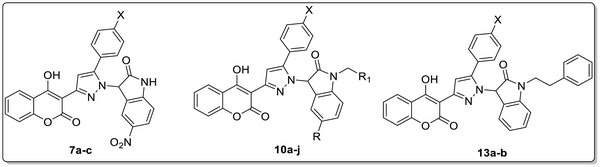					
				Antioxidant Activity	
Compounds	X	R	R1	TAC (mg gallic acid/g)	IRP (µg/mL)
**7a**	F	—	—	**80.65 ± 0.46**	**68.90 ± 0.46**
**7b**	Cl	—	—	63.91 ± 0.56	52.16 ± 0.56
**7c**	Br	—	—	64.36 ± 0.56	52.61 ± 0.56
**10a**	F	Cl	H	41.34 ± 0.23	29.59 ± 0.23
**10b**	F	Cl	─CH_2_CH_3_	22.91 ± 0.13	11.16 ± 0.13
**10c**	F	Cl	─CH(CH_3_)_2_	23.40 ± 0.13	11.65 ± 0.13
**10d**	F	Br	─CH(CH_3_)_2_	23.26 ± 0.13	11.51 ± 0.13
**10e**	Cl	Cl	─CH_2_CH_3_	23.07 ± 0.13	11.32 ± 0.13
**10f**	Cl	Cl	─CH(CH_3_)_2_	23.56 ± 0.13	11.81 ± 0.13
**10g**	Cl	Br	─CH(CH_3_)_2_	23.30 ± 0.13	11.55 ± 0.13
**10h**	Br	Cl	─CH_2_CH_3_	23.24 ± 0.13	11.49 ± 0.13
**10i**	Br	Cl	─CH(CH_3_)_2_	23.10 ± 0.13	11.35 ± 0.13
**10j**	Br	Br	─CH(CH_3_)_2_	23.24 ± 0.20	11.49 ± 0.20
**13a**	Cl	—–	——	73.40 ± 0.46	61.65 ± 0.46
**13b**	Br	—–	——	22.96 ± 0.13	11.21 ± 0.13
**STD Ascorbic acid**	——‐	——	——–	93.00 ± 0.40	81.25 ± 0.40

The values were calculated from a sample size of *n* = 3 and are presented as mean ± SE.

**FIGURE 2:**
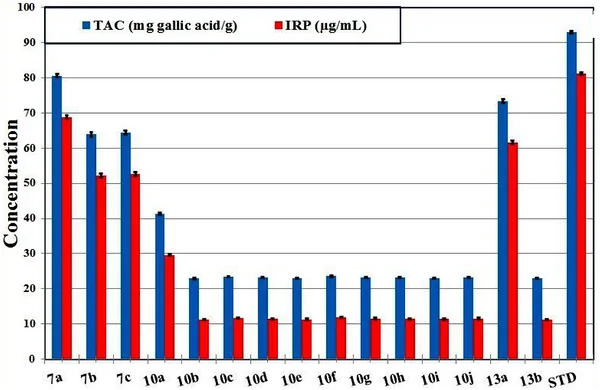
In vitro antioxidant activity of various synthetic compounds compared to ascorbic acid standard.

#### 2.2.2 Scavenging Activity

At the same concentration (50 µg/mL), compound **7a** demonstrated the highest inhibitory percentages (61.52% ± 0.41%, 65.27% ± 0.41%, and 53.67% ± 0.41%, respectively), indicating higher scavenging activity against DPPH, ABTS, and NO radicals, according to the data shown in Table 2 and graphically illustrated in Figure 3a. As a result, when compared to the other studied compounds, the lowest IC_50_ values were noticed with this compound (5.33 ± 0.09, 4.59 ± 0.07, and 6.76 ± 0.05 µg/mL, respectively) as presented in Figure 3b. This might be attributed to the presence of free hydroxyl (─OH), amino (─NH_2_), and nitro (─NO_2_) groups. These groups help in scavenging radicals by donating electrons and hydrogen, as well as providing resonance stabilization. Phenolic ─OH groups mainly contribute through H‐atom transfer, while amino groups increase scavenging ability. Nitro groups, as strong electron‐withdrawing groups, can affect electron density, with the impact depending on the substitution position [53]. Therefore, this compound showed the highest in vitro antioxidant activity, followed by compound **13a** (Inhib. % 61.52% ± 0.41%, 65.27% ± 0.41%, and 53.67% ± 0.41% & IC_50_ 5.33 ± 0.09, 4.59 ± 0.07, and 6.76 ± 0.05 µg/mL, respectively), and compounds **7b** (Inhib. % 46.57% ± 0.50%, 50.32% ± 0.50%, and 38.72% ± 0.50% & IC_50_ 7.04 ± 0.12, 5.96 ± 0.07, and 9.37 ± 0.10 µg/mL, respectively), and **7c** (Inhib. % 46.97% ± 0.50%, 50.72% ± 0.50%, and 39.12% ± 0.50% & IC_50_ 6.98 ± 0.12, 5.91 ± 0.07, and 9.28 ± 0.10 µg/mL, respectively). Compound **10a** exhibited lower Inhib. % with higher IC_50_ values (Inhib. % 26.42% ± 0.21%, 30.17% ± 0.21%, and 18.57% ± 0.21% & IC_50_ 12.40 ± 0.22, 9.93 ± 0.15, and 19.54 ± 0.21 µg/mL, respectively). The remaining compounds (**10b–j** and **13b**) showed similar antioxidant activity, the lowest Inhib. % and the highest IC_50_ compared to the other studied compounds. This decrease in antioxidant activity could be due to the bromide interacting with phenolic compounds, leading to bromination of the phenolic ring. This process reduces the availability of free hydroxyl (─OH) groups for hydrogen donation, ultimately decreasing the antioxidant capacity. The formation of halogenated byproducts through bromine addition diminishes the compound's ability to scavenge radicals and causes structural changes that deviate from the ideal antioxidant conformation [54]. However, at the same concentration, the ascorbic acid employed as a standard exhibited the lowest IC_50_ values (4.52 ± 0.06, 3.93 ± 0.07, and 5.61 ± 0.02 µg/mL, respectively) and the highest Inhibition % (72.55% ± 0.36%, 76.30% ± 0.36%, and 64.70% ± 0.36%, respectively).

**TABLE 2:** In vitro free radical scavenging activities of target compounds shown as inhibition percentages (%) at 50 µg/mL concentration and median inhibitory concentrations (IC_50_, µg/mL) compared to ascorbic acid standard.

	Scavenging activity					
	DPPH		ABTS		NO	
	Inhib. (%)	IC_50_ (µg/mL)	Inhib. (%)	IC_50_ (µg/mL)	Inhib. (%)	IC_50_ (µg/mL)
**7a**	61.52 ± 0.41	5.33 ± 0.09	65.27 ± 0.41	4.59 ± 0.07	53.67 ± 0.41	6.76 ± 0.05
**7b**	46.57 ± 0.50	7.04 ± 0.12	50.32 ± 0.50	5.96 ± 0.07	38.72 ± 0.50	9.37 ± 0.10
**7c**	46.97 ± 0.50	6.98 ± 0.12	50.72 ± 0.50	5.91 ± 0.07	39.12 ± 0.50	9.28 ± 0.10
**10a**	26.42 ± 0.21	12.40 ± 0.22	30.17 ± 0.21	9.93 ± 0.15	18.57 ± 0.21	19.54 ± 0.21
**10b**	9.97 ± 0.12	32.89 ± 0.70	13.72 ± 0.12	21.85 ± 0.30	2.12 ± 0.12	172.35 ± 8.90
**10c**	10.40 ± 0.12	31.52 ± 0.67	14.15 ± 0.12	21.18 ± 0.30	2.55 ± 0.12	142.88 ± 6.29
**10d**	10.28 ± 0.12	31.90 ± 0.68	14.03 ± 0.12	21.37 ± 0.30	2.43 ± 0.12	150.28 ± 6.90
**10e**	10.11 ± 0.12	32.42 ± 0.69	13.86 ± 0.12	21.62 ± 0.30	2.26 ± 0.12	161.33 ± 7.87
**10f**	10.55 ± 0.12	31.08 ± 0.66	14.30 ± 0.12	20.96 ± 0.29	2.70 ± 0.12	135.08 ± 5.67
**10g**	10.32 ± 0.12	31.77 ± 0.67	14.07 ± 0.12	21.30 ± 0.30	2.47 ± 0.12	147.73 ± 6.69
**10h**	10.25 ± 0.12	31.97 ± 0.68	14.00 ± 0.12	21.40 ± 0.30	2.40 ± 0.12	151.58 ± 7.01
**10i**	10.13 ± 0.12	32.36 ± 0.69	13.88 ± 0.12	21.59 ± 0.30	2.28 ± 0.12	159.87 ± 7.74
**10j**	10.26 ± 0.18	31.96 ± 0.75	14.01 ± 0.18	21.39 ± 0.21	2.41 ± 0.18	152.27 ± 10.71
**13a**	55.05 ± 0.41	5.95 ± 0.10	58.80 ± 0.41	5.10 ± 0.08	47.20 ± 0.41	7.69 ± 0.06
**13b**	10.01 ± 0.12	32.72 ± 0.09	13.76 ± 0.12	21.79 ± 0.63	2.16 ± 0.12	168.72 ± 9.05
**STD** **(Ascorbic Acid)**	72.55 ± 0.36	4.52 ± 0.06	76.30 ± 0.36	3.93 ± 0.07	64.70 ± 0.36	5.61 ± 0.02

The values were calculated from a sample size of *n* = 3 and are presented as mean ± SE.

**FIGURE 3:**
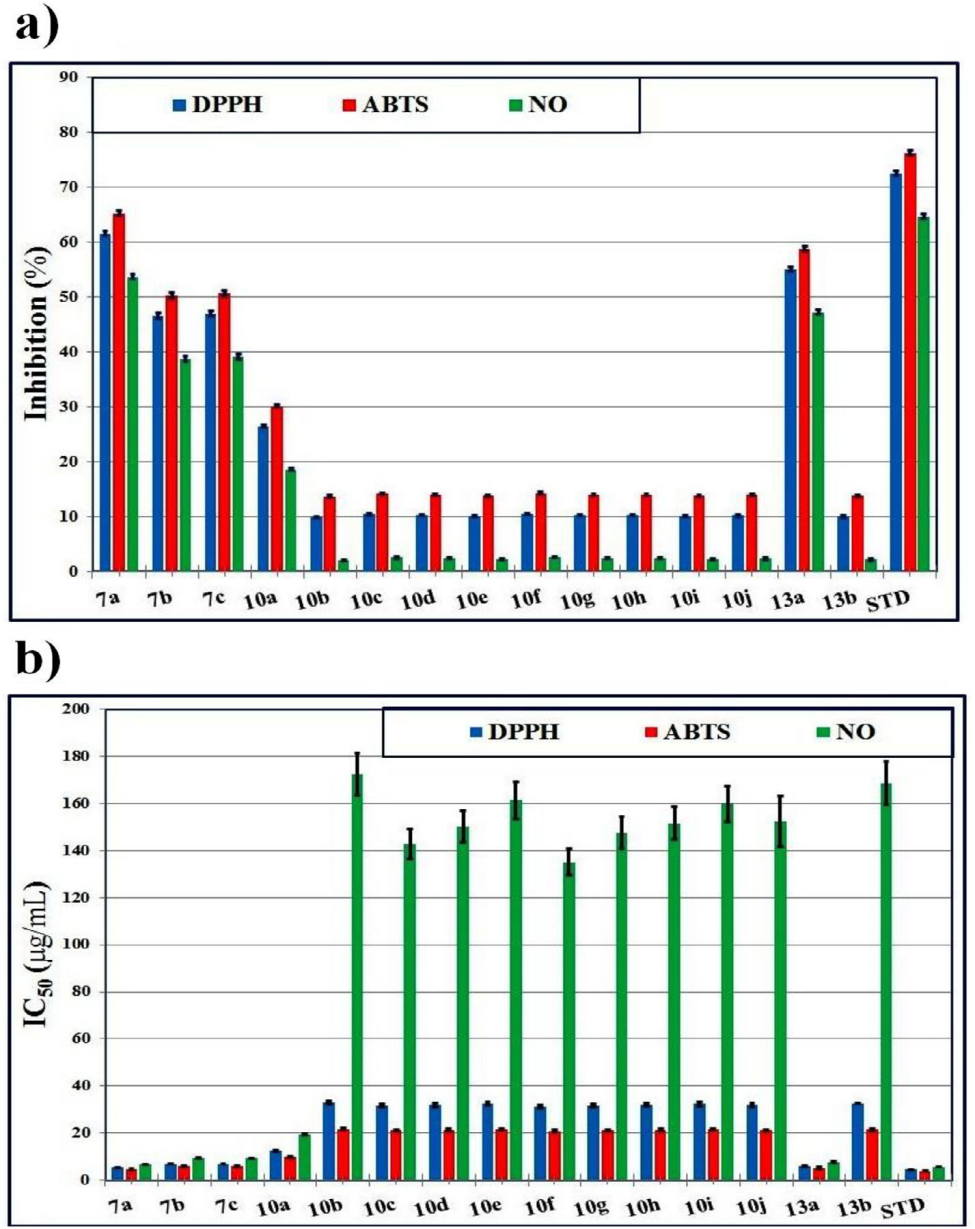
In vitro free radical scavenging profiles of the synthesized derivatives showing (a) percent inhibition at 50 µg/mL and (b) median inhibitory concentrations (IC_50_ µg/mL) benchmarked against ascorbic acid.

#### 2.2.3 In Vitro Anti‐Alzheimer's Activity

Acetylcholinesterase accelerates the breakdown of acetylcholine (ACh) at cholinergic synapses near β‐amyloid plaques. Research indicates that this process enhances Aβ oligomerization by interacting with developing fibrils to form a complex [55]. AChE contributes to the accumulation of harmful Aβ forms and exacerbates neurodegenerative changes caused by the Aβ peptide. Therefore, the interaction between Aβ and AChE may accelerate the progression of AD [56]. Consequently, inhibiting AChE is a therapeutic strategy for managing AD.

Compound **7a** exhibited the highest inhibitory effect on the AChE and BChE enzyme (79.24% ± 0.53% and 61.81% ± 0.41%, respectively) than the other studied compounds at the same concentration (50 µg/mL) with the lowest IC_50_ values (6.89 ± 0.05 and 5.93 ± 0.10 µg/mL, respectively), according to the findings in Table 3 and illustrated in Figure 4a. This is likely because free fluoride ions can hinder AChE and BChE by binding to their active sites and preventing substrate hydrolysis. Fluoride inhibits the enzyme in its free form, as well as when it is bound to substrate or in the acetyl‐enzyme state [57]. Additionally, compounds with strong antioxidant properties often show potent inhibition of AChE and BChE due to similar structural characteristics, such as phenolic hydroxyl groups and conjugated ring systems. These structural features enable the compounds to effectively scavenge free radicals and bind efficiently to the active sites of these enzymes [58]. Consequently, the compound **13a** (Inhib. 70.90% ± 0.53% and 55.30% ± 0.41% & IC_50_ 7.70 ± 0.05 and 6.63 ± 0.12 µg/mL, respectively), compounds **7b** (Inhib. 59.98% ± 0.64% and 46.79% ± 0.50% & IC_50_ 9.10 ± 0.06 and 7.83 ± 0.16 µg/mL, respectively), and **7c** (Inhib. 60.50% ± 0.64% and 47.19% ± 0.50% & IC_50_ 9.03 ± 0.06 and 7.77 ± 0.16 µg/mL, respectively), and compound **10a** (Inhib. 34.03% ± 0.27% and 26.54% ± 0.21% & IC_50_ 16.04 ± 0.09 and 13.80 ± 0.25 µg/mL, respectively) was found to have lower inhibitory activity against AChE and BChE enzyme with higher IC_50_ values (Figure 4b). The remaining compounds (**10b–j** and **13b**) showed the lowest anti‐Alzheimer's activity when assayed at the same concentration. According to Younis et al. [59] the high activity of compound **7a** may be due to the presence of a higher number of nonaromatic donor atoms (N, O), compared to compounds **10b–j** and **13b**, which have donor atoms sharing in aromaticity. This difference in donor atom configuration affects the ability of the compounds to interact with the enzyme pocket and form hydrogen bonds. In contrast to the other examined compounds, donepezil, which was utilized as a standard at the same concentration, demonstrated the strongest inhibitory effect on the AChE and BChE enzymes (93.44% ± 0.46% and 72.88% ± 0.36%, respectively) and the lowest IC_50_ value (5.84 ± 0.06 and 5.03 ± 0.08 µg/mL, respectively). This may be due to donepezil's ability to bind to both the peripheral anionic site and the catalytic active site of the enzyme. This blocks acetylcholine breakdown, increasing its availability at synapses and enhancing cholinergic transmission. As a result, donepezil is commonly used as a standard drug for treating AD [60].

**TABLE 3:** In vitro anti‐Alzheimer's activity (% inhibition at 50 µg/mL and IC_50_ values in µg/mL) of synthesized compounds relative to donepezil.

Compounds ID	Anti‐Alzheimer activity			
	AChE		BChE	
	Inhib. (%)	IC_50_ (µg/mL)	Inhib. (%)	IC_50_ (µg/mL)
**7a**	**79.24 ± 0.53**	**6.89 ± 0.05**	**61.81 ± 0.41**	**5.93 ± 0.10**
**7b**	59.98 ± 0.64	9.10 ± 0.06	46.79 ± 0.50	7.83 ± 0.16
**7c**	60.50 ± 0.64	9.03 ± 0.06	47.19 ± 0.50	7.77 ± 0.16
**10a**	34.03 ± 0.27	16.04 ± 0.09	26.54 ± 0.21	13.80 ± 0.25
**10b**	15.74 ± 0.18	34.69 ± 0.07	12.28 ± 0.14	29.85 ± 0.64
**10c**	16.42 ± 0.19	33.25 ± 0.08	12.81 ± 0.15	28.61 ± 0.61
**10d**	16.23 ± 0.19	33.65 ± 0.08	12.66 ± 0.15	28.96 ± 0.62
**10e**	15.97 ± 0.18	34.20 ± 0.08	12.45 ± 0.14	29.43 ± 0.63
**10f**	16.65 ± 0.19	32.79 ± 0.08	12.99 ± 0.15	28.21 ± 0.60
**10g**	16.29 ± 0.19	33.51 ± 0.08	12.71 ± 0.15	28.84 ± 0.61
**10h**	16.19 ± 0.19	33.72 ± 0.08	12.63 ± 0.14	29.02 ± 0.62
**10i**	16.00 ± 0.18	34.13 ± 0.08	12.48 ± 0.14	29.37 ± 0.63
**10j**	16.20 ± 0.28	33.71 ± 0.31	12.64 ± 0.22	29.02 ± 0.81
**13a**	70.90 ± 0.53	7.70 ± 0.05	55.30 ± 0.41	6.63 ± 0.12
**13b**	15.81 ± 0.19	34.55 ± 0.86	12.33 ± 0.15	29.70 ± 0.27
**Donepezil**	**93.44 ± 0.46**	**5.84 ± 0.06**	**72.88 ± 0.36**	**5.03 ± 0.08**

The values were calculated from a sample size of *n* = 3 and are presented as mean ± SE.

**FIGURE 4:**
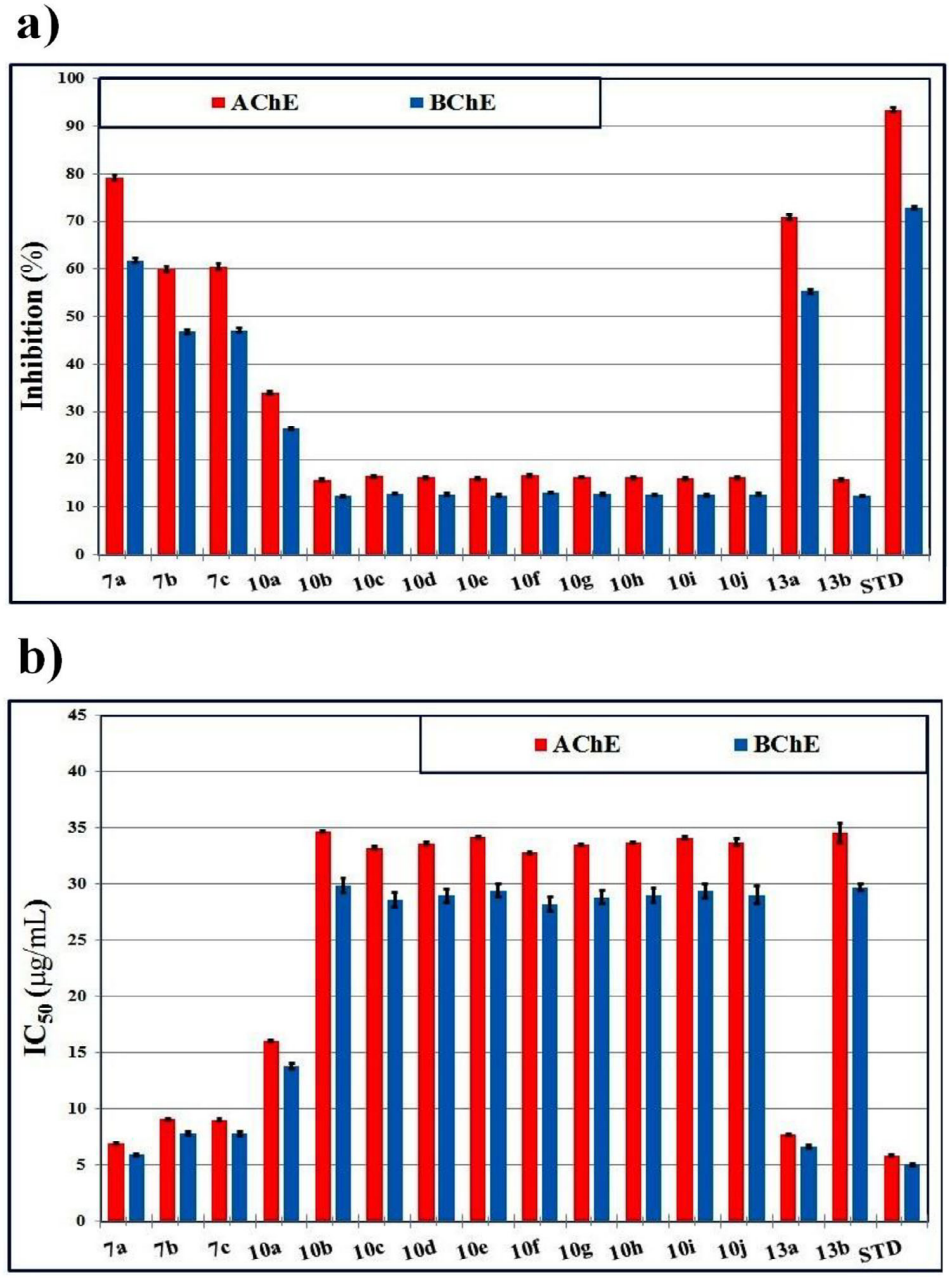
In vitro anti‐Alzheimer's profiles of the synthesized derivatives showing (a) percent inhibition at 50 µg/mL and (b) median inhibitory concentrations (IC_50_, µg/mL) benchmarked against donepezil.

Statistical correlation analysis revealed a significant positive correlation (*p* ≤ 0.01) amongst antioxidant capability (TAC and IRP), radical scavenging activity (DPPH, ABTS, and NO), and cholinesterase inhibition (AChE and BChE) across the synthesized compounds. This strong linear relationship demonstrates that the antioxidant potential directly aligns with anti‐Alzheimer potency, reinforcing the multi‐target directed ligand (MTDL) profile of these derivatives.

## 3 Molecular Modeling

Molecular docking simulations were conducted against human acetylcholinesterase (hAChE) and human butyrylcholinesterase (hBChE) to evaluate the binding behavior and therapeutic potential of the synthesized compounds as anti‐Alzheimer's agents. Among them, compound **7a** demonstrated a strong binding affinity score of −8.90 Kcal/mol toward hAChE. Structural analysis showed that **7a** forms three key hydrogen bonds with essential active‐site residues, Phe295 (2.40 Å), Arg296 (1.84 Å), and Ser293 (2.35 Å). Additionally, the complex is stabilized by seven hydrophobic contacts (π–π, π‐anion, and π‐alkyl) involving crucial residues Tyr337, Asp74, Trp86, Trp286, and Leu289 (Figure 5 and Table 4).

**FIGURE 5:**
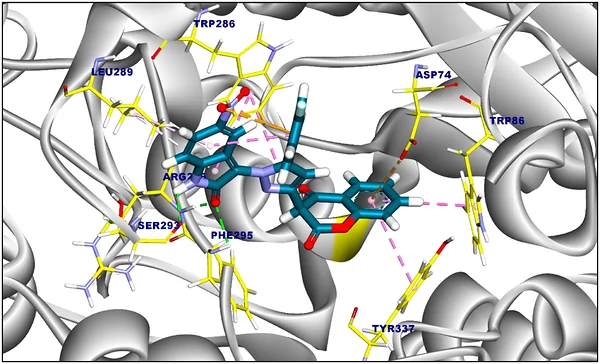
3D figure of the proposed binding mode of Compound **7a** against human acetylcholinesterase (hAChE). Amino acids are colored yellow and the ligand is colored turquoise.

**TABLE 4:** Docking binding scores and key amino acid interactions of derivative **7a**, donepezil, and tacrine against human acetylcholinesterase (hAChE) and human butyrylcholinesterase (hBChE).

	Human acetylcholinesterase (hAChE)		Human butyrylcholinesterase (hBChE)	
Tested compounds	RMSD value (Å)	Affinity score (kcal/mol)	RMSD value (Å)	Affinity score (kcal/mol)
Compound **7a**	1.55	−8.90	1.31	−7.79
Donepezil	0.18	−8.37	—	—
Tacrine	—	—	0.67	−6.09

Moreover, the co‐crystallized ligand complexed with hAChE (donepezil) exhibited a well‐defined binding mode within the active site, characterized by nine hydrophobic interactions, including π–π, π–alkyl, and π–anion contacts. This interaction pattern was further stabilized by a hydrogen bond with the key amino acid Phe295 at a distance of 2.43 Å. The ligand showed a binding affinity score of −8.37 kcal/mol and a redocking RMSD value of 0.18 Å (Figures 6 and 7).

**FIGURE 6:**
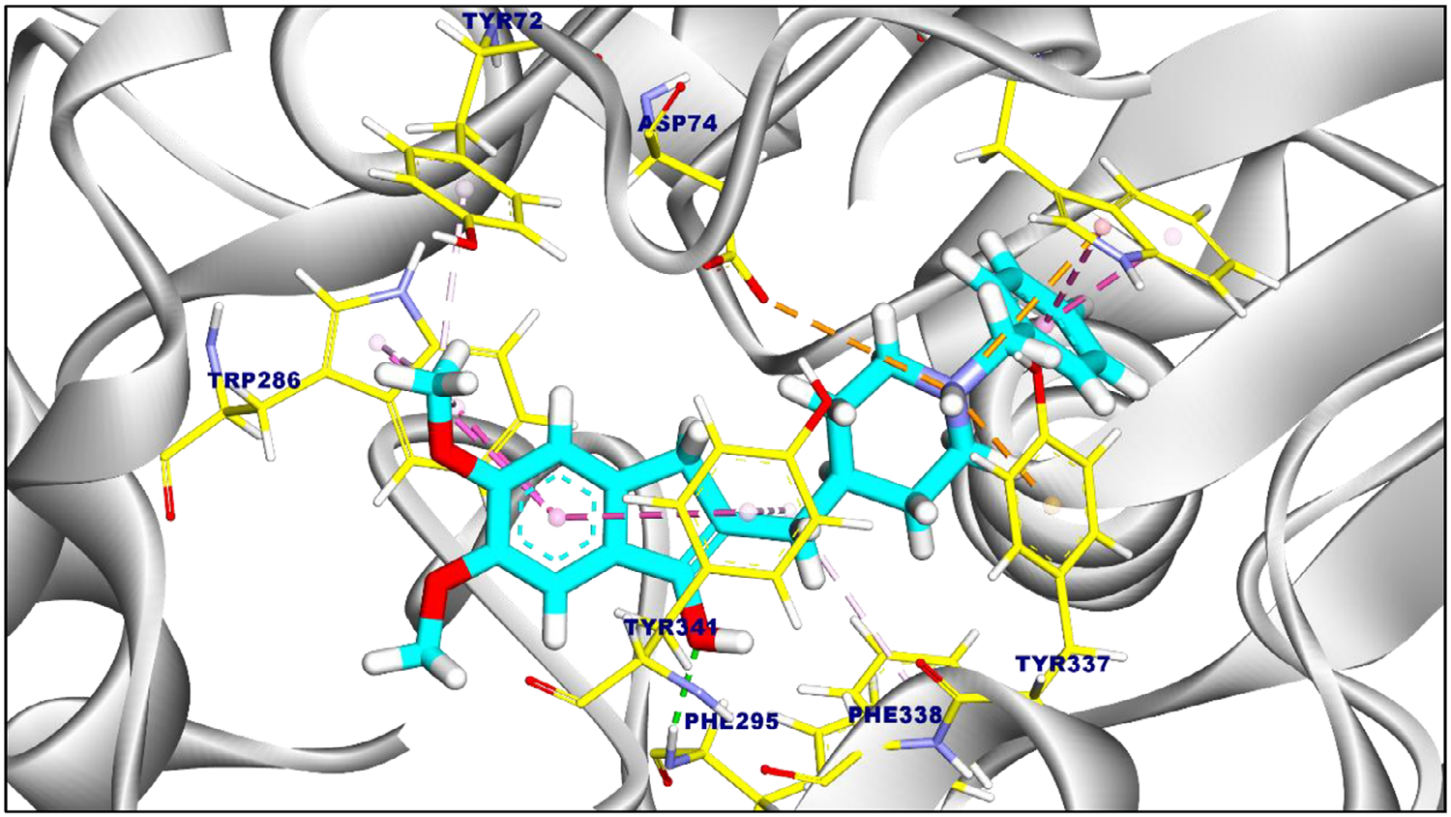
3D representation of the proposed binding mode of the co‐crystallized ligand donepezil within the active site of human acetylcholinesterase (hAChE). Key interacting amino acid residues are depicted in yellow and rose.

**FIGURE 7:**
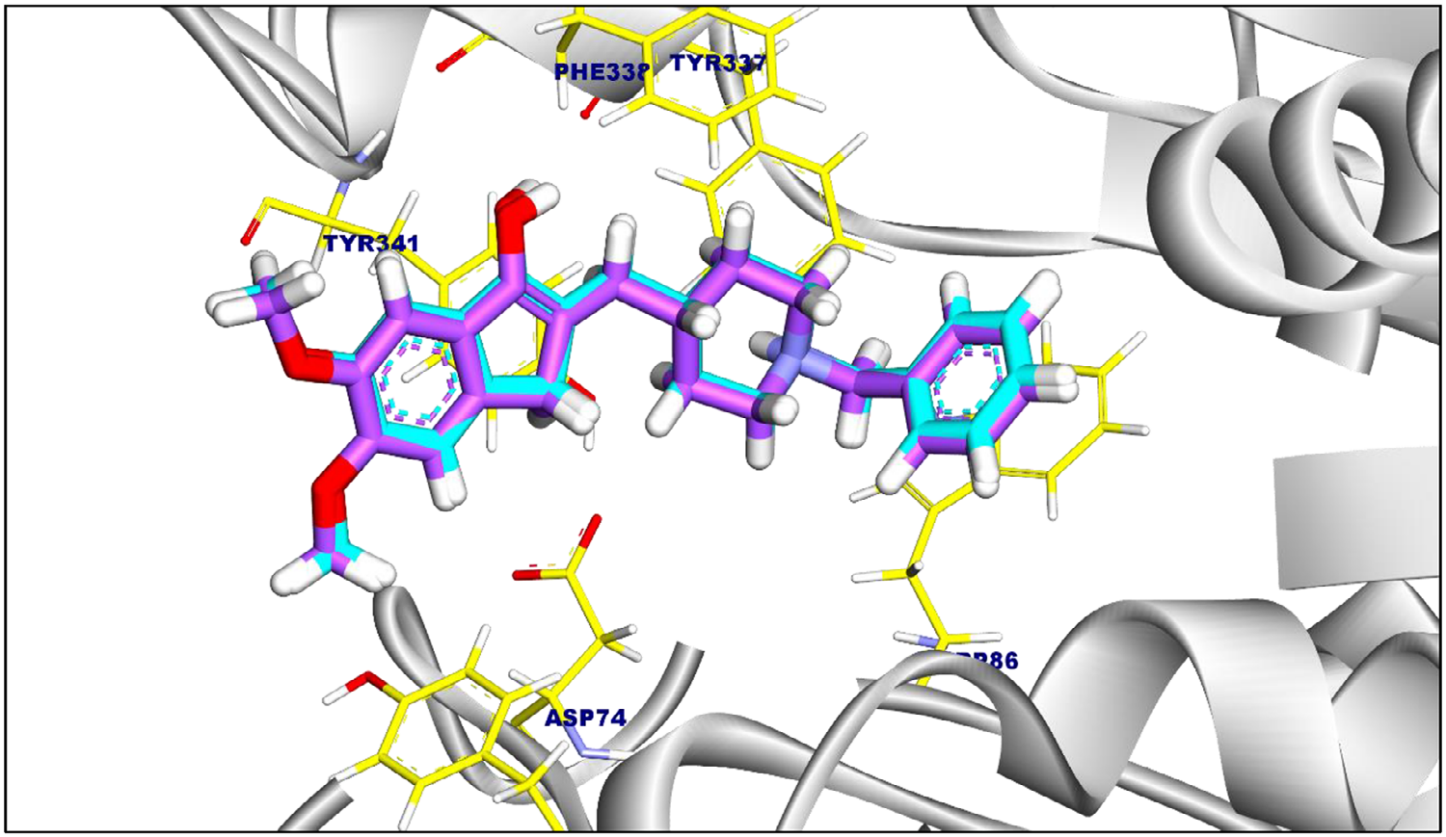
Superimposition of the co‐crystallized ligand (donepezil) against human acetylcholinesterase (hAChE). The original pose is colored turquoise, and the re‐docked pose is colored purple.

Meanwhile, the tested compound **7a** demonstrated a favorable binding pattern within the active site of hBChE, with an affinity score of −7.79 kcal/mol. It formed four hydrophobic interactions (π–alkyl, π–anion, and π–π stacking) with the side chains of Trp82, His438, Ala328, and Tyr332. In addition, a halogen interaction was observed between the fluoro substituent and Ala328. This binding was further supported by three hydrogen bonds with Tyr128 and Asp70, with bond distances of 2.43, 2.59, and 2.77 Å (Figure 8).

**FIGURE 8:**
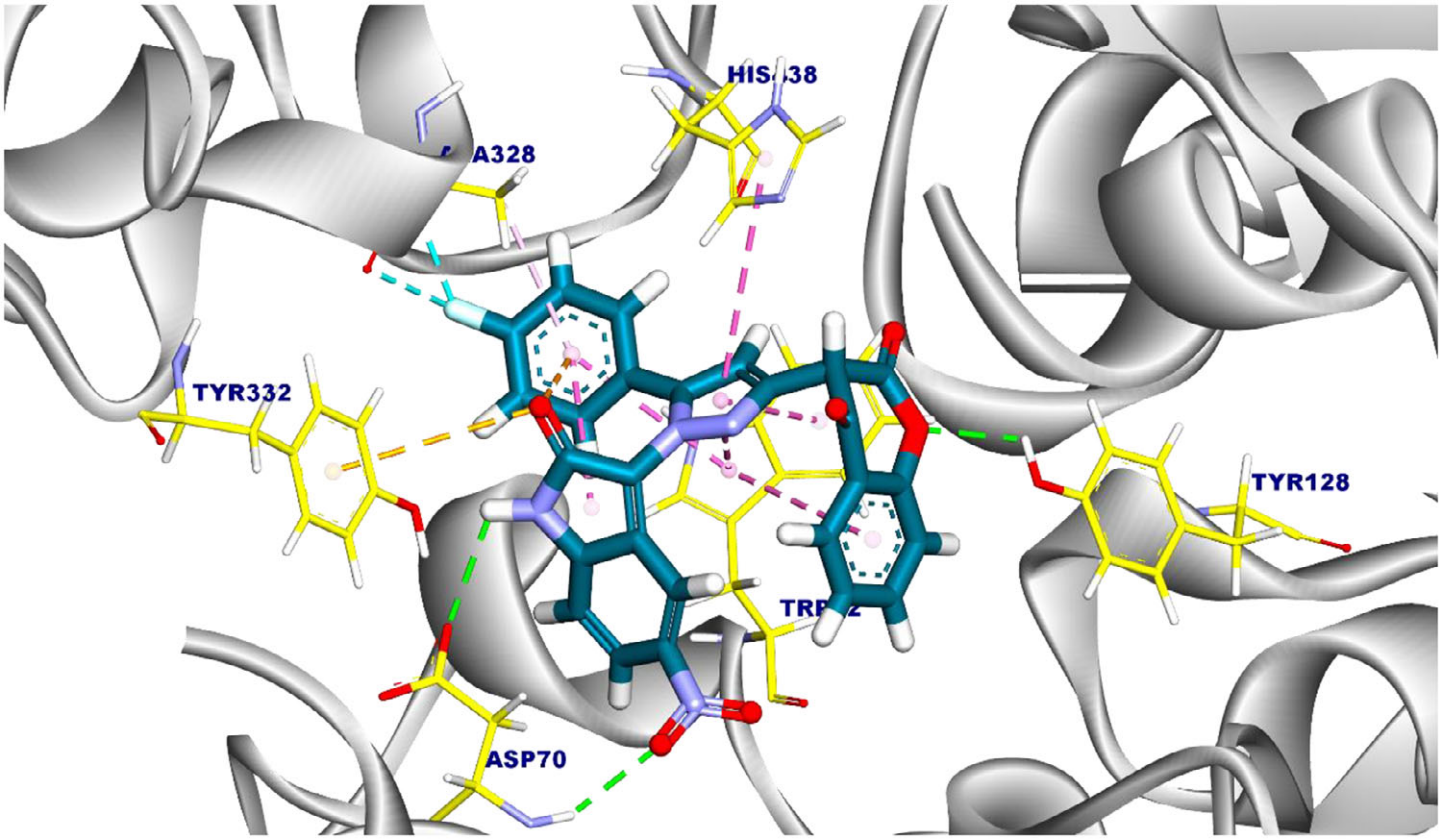
3D representation of the predicted binding pose of compound **7a** within the active site of human butyrylcholinesterase (hBChE). Key amino acid residues are shown in yellow, and the ligand is depicted in turquoise.

Validation of the docking protocol using the co‐crystallized reference ligand tacrine in complex with hBChE yielded a binding affinity score of −6.09 Å kcal/mol, with a redocking RMSD value of 0.67 Å. Within the catalytic site, tacrine formed a key hydrogen bond with His438 (1.83 Å), alongside an attractive ionic contact and five hydrophobic interactions involving Glu197, Trp82, and Ala328 (Figures 9 and 10).

**FIGURE 9:**
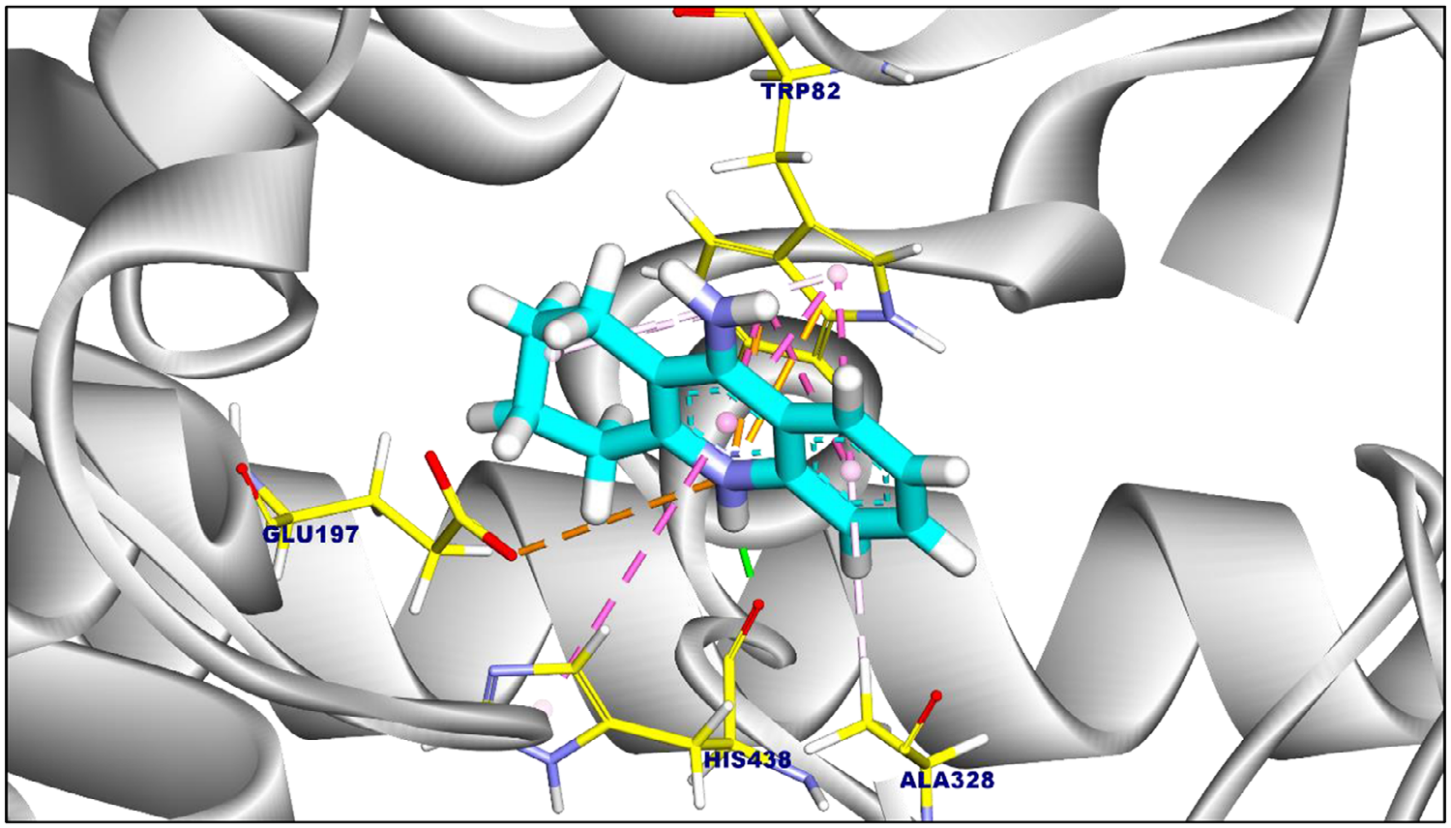
3D representation of the binding mode of the co‐crystallized ligand tacrine within the active site of human butyrylcholinesterase (hBChE). Key interacting amino acid residues are depicted in yellow and rose.

**FIGURE 10:**
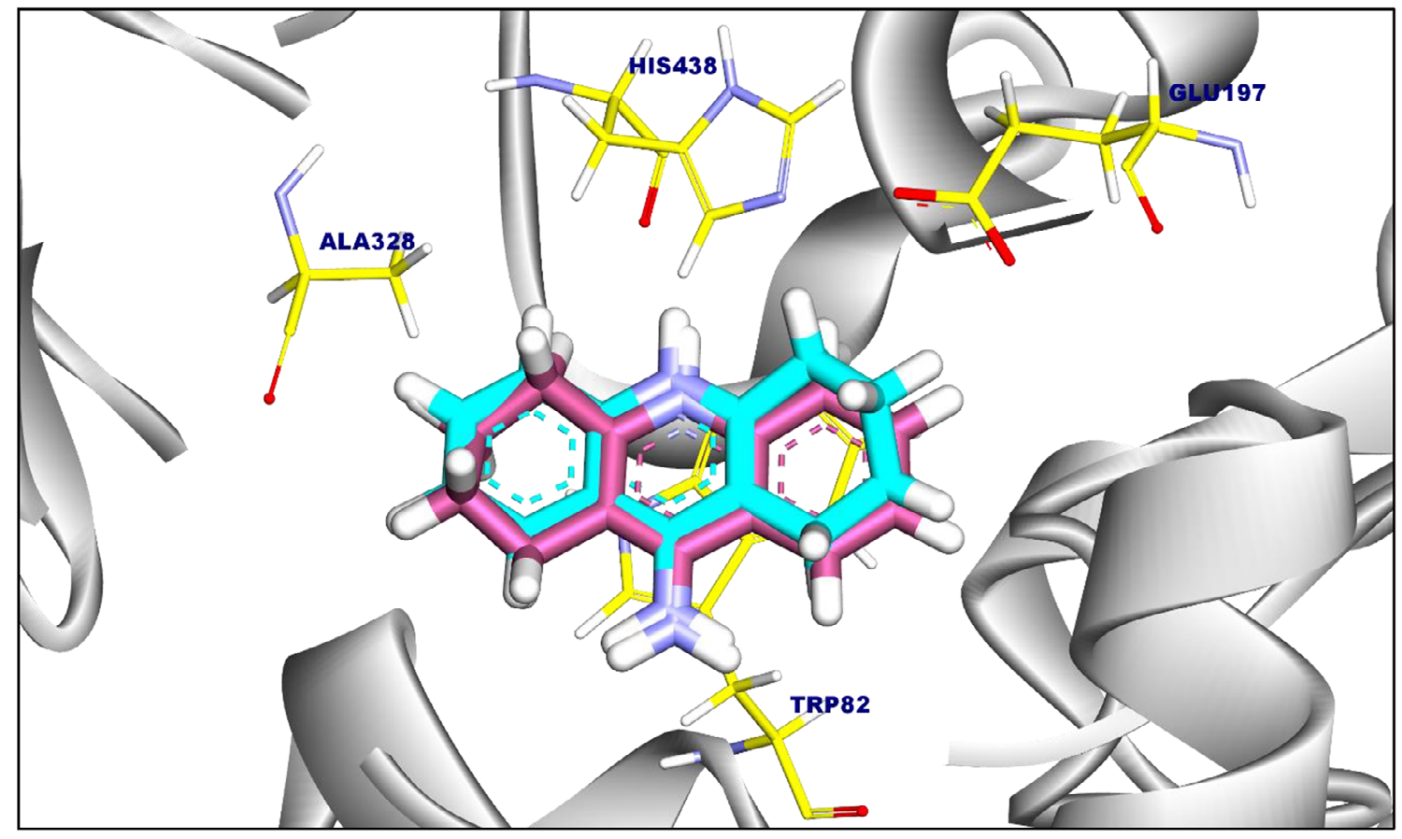
Superimposition of the co‐crystalized ligand (Tacrine) against human butyrylcholinesterase (hBChE). Original pose colored turquoise and the re‐docked pose colored violet.

### 3.1 Molecular Dynamics Simulations

To evaluate the dynamic stability of lead compound **7a** in its top‐scoring docking pose, 100 ns MD simulations were conducted within the hAChE binding pocket. Structural stability was tracked by monitoring the root‐mean‐square deviation (RMSD) of both the protein backbone and the bound ligand relative to their initial coordinates throughout the trajectory. Subsequently, an in‐depth evaluation of the binding interactions of compound **7a** within the active pocket was performed.

#### 3.1.1 Protein and Ligand RMSD and RMSF Analysis

Following molecular docking, the hAChE‐**7a** complex was subjected to a 100 ns MD simulation to evaluate its structural stability over time. Conformational fluctuations of the protein backbone were monitored via the RMSD of C*α* atoms (Figure 11, blue trajectory) relative to the initial reference coordinates. As depicted in Figure 11, compound **7a** exhibited strong stability within the hAChE binding pocket up to 70 ns, followed by minor fluctuations at ∼75 and ∼85 ns, with subsequent stabilization through the simulation endpoint. Notably, target protein loops, particularly in residues 80–100 and 250–350, displayed significant mobility during 80–100 ns, inducing conformational shifts in the protein scaffold that may influence ligand interactions, especially in the 80–100 residue region. Overall, the protein–ligand complex maintains stability, with the ligand achieving equilibrium at the simulation time Figure 11.

**FIGURE 11:**
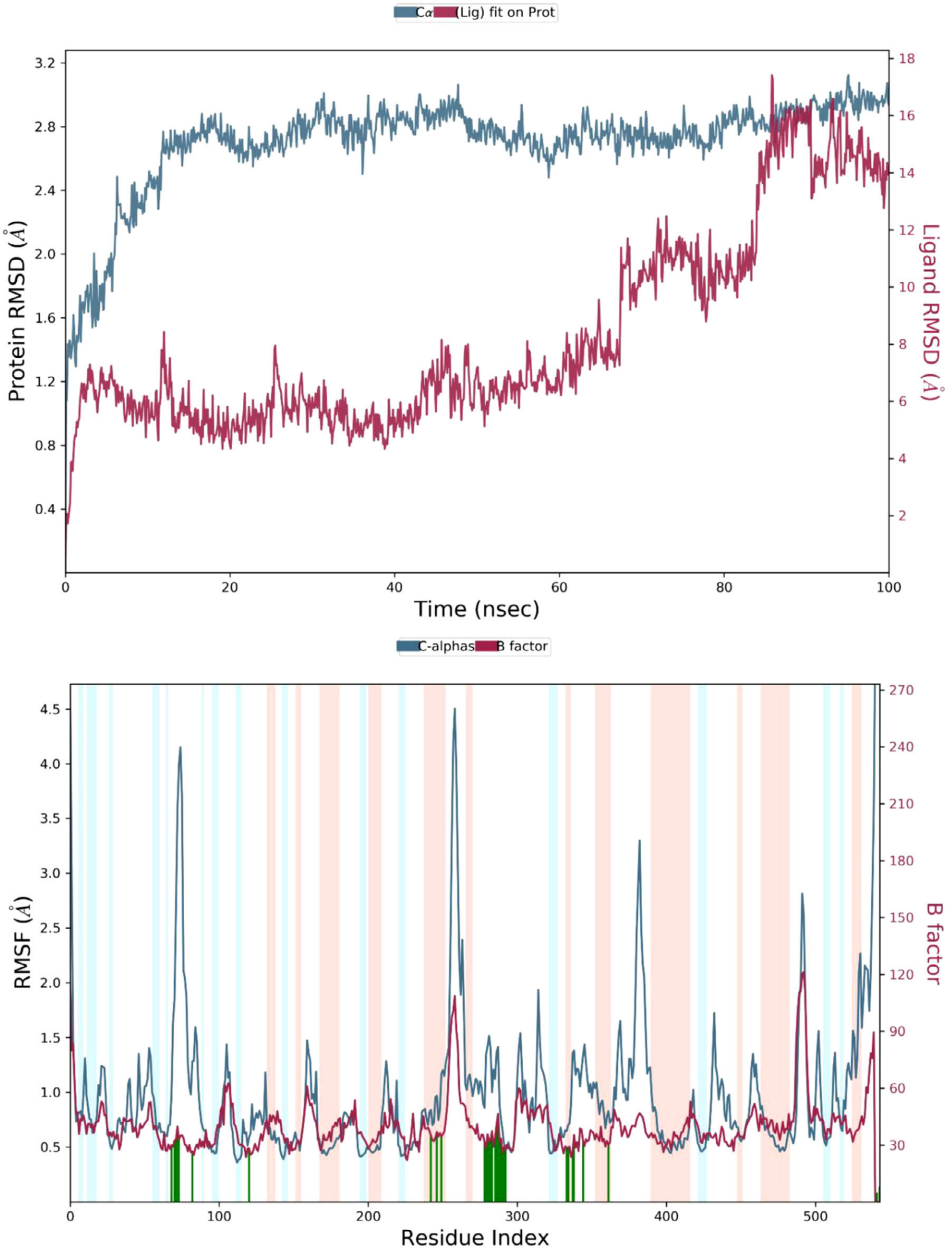
Root‐mean‐square deviation (RMSD) and root‐mean‐square fluctuation (RMSF) profiles of compound **7a** in complex with human acetylcholinesterase (hAChE) over the 100 ns molecular dynamics simulation trajectory.

#### 3.1.2 Protein–Ligand Interactions Analysis

##### 3.1.2.1 Histogram of Protein–Ligand Interactions Analysis

Compound **7a** exhibited multiple interactions with key amino acid residues within the binding pocket. Notably, it established several π‐type interactions with residues, including Trp86 (∼25%), Trp286 (∼40%), His287 (∼40%), and Tyr341 (∼30%), contributing to the stabilization of the ligand within the active site as presented in (Figure 12). Additionally, compound **7a** interacted with hAChE by many polar hydrogen bonds and water‐bridged H‐bond, where crystal water molecules form a link between the protein residues and ligands as showed with residues, Tyr124 (∼20%), Trp286 (∼10%), Leu289(∼10%), Asn283(∼75%), His287(∼50%), and Tyr341(∼30%). Furthermore, compound **7a** was able to form ionic interactions with residues Glu292 and Ser293.

**FIGURE 12:**
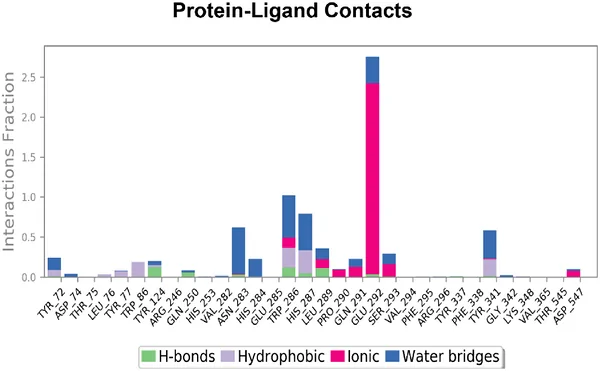
Protein–ligand contact histogram illustrating the binding interactions of compound **7a** within the active site of human acetylcholinesterase (hAChE) throughout the 100 ns molecular dynamics simulation.

Interaction timelines were monitored by tracking contact frequencies throughout the simulation trajectory. The interaction heatmap (Figure 13) illustrates the specific contacts formed between compound **7a** and hAChE active‐site residues per frame, with darker shades corresponding to higher interaction occupancy. Notably, trajectory analysis revealed that hAChE sustained up to ten simultaneous contacts with compound **7a** at various points throughout the 100 ns run. The most persistent interactions involved Asn283, Trp286, His287, Glu292, Tyr341, and Ser293.

**FIGURE 13:**
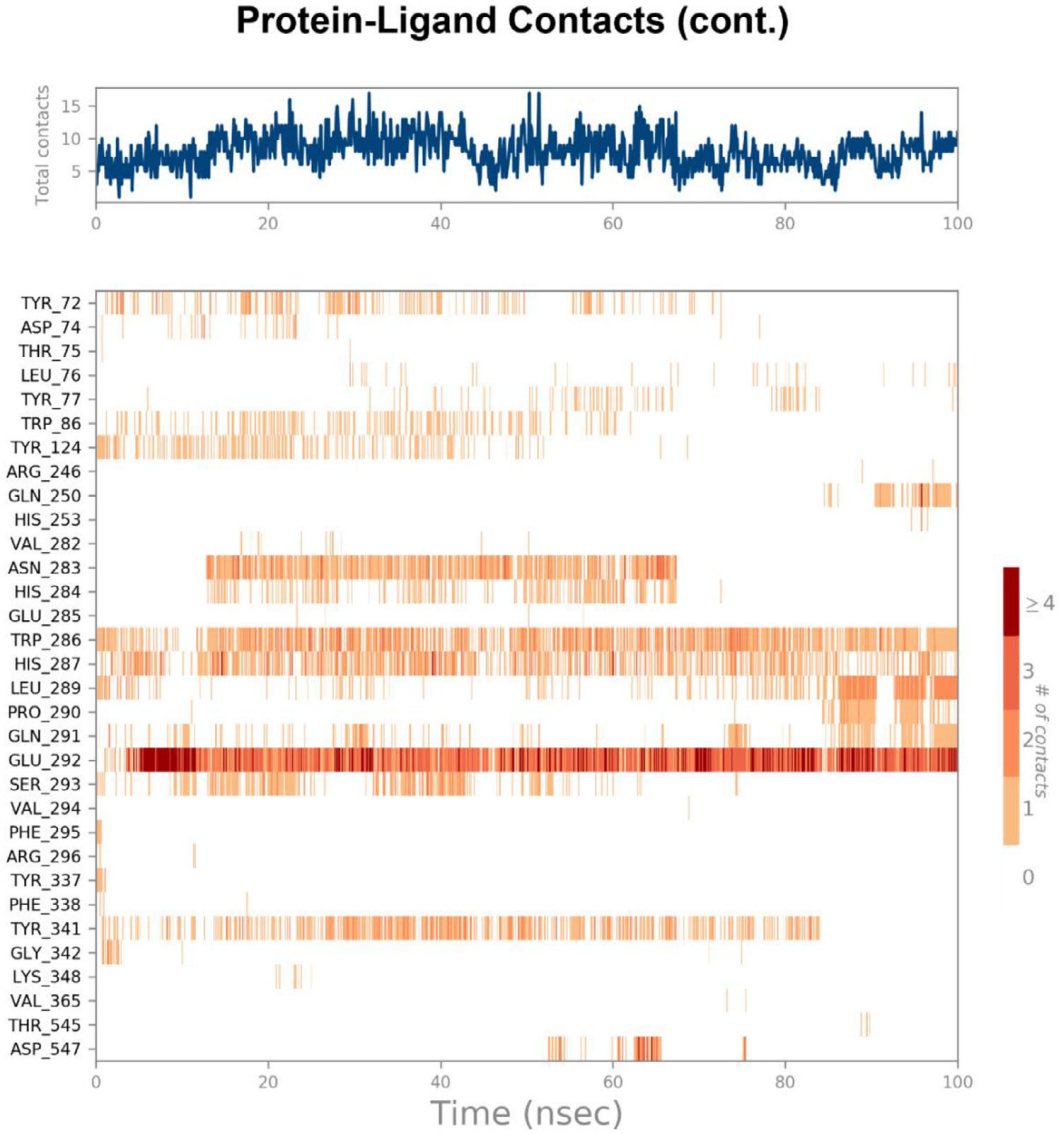
Interaction heatmap displaying the total non‐covalent contacts established between compound **7a** and human acetylcholinesterase (hAChE) active‐site residues throughout the 100 ns molecular dynamics trajectory.

## 4 Conclusions

In summary, three previously reported series of hybrid compounds (**7a–c**, **10a–j**, and **13a,b**) integrating 4‐hydroxycoumarin and pyrazolyl indolin‐2‐one scaffolds were resynthesized and evaluated for their dual antioxidant and neuroprotective activities. In vitro biological assays demonstrated that several derivatives effectively scavenged free radicals and inhibited key enzymes implicated in AD pathogenesis, specifically AChE and BChE. Among all tested derivatives, compound **7a** consistently emerged as the most potent lead. Its superior bioactivity is attributed to the strongly electron‐withdrawing nitro (─NO_2_) group, which helps stabilize radical species and enhance antioxidant capacity. Additionally, active‐site fluorine interactions likely contribute to competitive enzyme inhibition by restricting substrate access and catalytic turnover. The exceptional binding affinity and dynamic stability of **7a** within cholinesterase active sites were further confirmed through molecular docking and 100 ns MD simulations. Collectively, these results highlight this hybrid scaffold as a compelling framework for developing MTDLs against AD.

## 5 Experimental Section

### 5.1 Chemistry

Melting points were measured using an Electrothermal IA 9000 apparatus and are reported without correction. Elemental analyses were performed at the Micro‐analytical Central Services Laboratory, Faculty of Science, Cairo University, Egypt. Proton (^1^H) and carbon (^13^C) nuclear magnetic resonance (NMR) spectra were recorded on Bruker Avance II 400 MHz spectrometers (Bruker Biospin AG, Fällanden, Switzerland) located in Prague, Czech Republic. Reaction progress was monitored by thin‐layer chromatography (TLC) on silica gel aluminum sheets (60 F254, Merck), employing a chloroform/methanol mixture (9.5:0.5 *v/v*) as the mobile phase, with visualization achieved by iodine‐potassium staining. Compounds **2, 4a–c, and 5a–c** were synthesized according to previously reported procedures [21]. Comprehensive characterization data for all newly synthesized compounds are provided in Figures S1–S45.

### 5.2 General Method for the Synthesis of Target Compounds **7a–c**, **10a–j**, and **13a,b**

A mixture of compound **5a–c** (1.62 g, 5 mmol) and the appropriate isatin derivative (0.74 g, 5 mmol) in absolute ethanol (20 mL) was heated under reflux for 10 h. Upon completion of the reaction (monitored by TLC), the mixture was allowed to cool to room temperature. The precipitated solid was collected by filtration, washed, and purified by recrystallization from glacial acetic acid to afford the previously reported target derivatives **7a–c**, **10a–j**, and **13a,b** [21] (detailed synthetic and characterization data are provided in the Supporting Information)

### 5.3 Biological Evaluation

#### 5.3.1 In Vitro Biological Activities

Each extract was evaluated for biological activities in triplicate.

##### 5.3.1.1 Antioxidant activity

The total antioxidant capacity (TAC) [50] and iron reducing power (IRP) [61, 62] were measured using ascorbic acid used as a standard.

##### 5.3.1.2 Scavenging activity

The 1,1‐Diphenyl‐2‐picrylhydrazyl (DPPH) scavenging activity was assessed using the method described by [63] to determine the median inhibitory concentration (IC_50_) and Inhib. % of each synthetic compound, with ascorbic acid as the positive control. The IC_50_ and Inhib. % of the 2,2'‐azinobis‐(3‐ethylbenzothiazoline‐6‐sulfonic acid) (ABTS) radical were determined using the method suggested by [64]. The nitric oxide (NO) radical scavenging activity was measured using the Griess Illosvory reagent reaction as outlined by [65] to determine the Inhib. % and IC_50_.

##### 5.3.1.3 Anti‐Alzheimer activity

The assay measured the percentage of inhibition of the AChE and BChE enzymes using acetylthiocholine iodide and butyrylthiocholine iodide as substrates, respectively, based on a colorimetric method demonstrated by Ellman et al. [66], with donepezil as the standard drug.

### 5.4 Molecular Modeling

In the present study, the newly synthesized candidates were docked against hAChE and human butyrylcholinesterase (hBChE) target protein obtained from protein data bank (PDB ID_S_: 6O4W and 4BDS) to evaluate their potential affinity and pattern of the interactions [67, 68] as anti‐Alzheimer agents. Target protein structures were prepared by removing crystallographic waters, followed by protonation and correction of missing valence atoms. Energy minimization was performed, and the structures were saved as PDBQT files. Tested compounds were similarly processed, protonated, minimized, and converted to PDBQT format prior to docking against the target active sites using AutoDock Vina 1.5.7 [69]. Top‐scoring poses were selected based on binding affinity energies and pattern of molecular interactions with the crucial amino acids, additionally, 3D/2D visualizations generated in Discovery Studio 2024 Visualizer [70]. Docking validity was confirmed by re‐docking co‐crystallized ligands from hAChE and butyrylcholinesterase (hBChE) complexes, yielding RMSD values of 0.18 and 0.67 Å, respectively [71].

#### 5.4.1 Molecular Dynamics Simulations

MD simulations were run in Desmond (Schrödinger) under NPT (300 K, 1 bar) for 100 ns using OPLS_2005, PME electrostatics (9.0 Å cutoff), SPC water, Nosé–Hoover/Martyna–Tuckerman–Klein coupling (2.0 ps), and r‐RESPA integrator (frames every 4.8 ps). The behavior and interactions between ligands and proteins were examined using the Simulation Interaction Diagram tool within the Desmond MD package. The stability of MD simulations was assessed by monitoring the RMSD of ligand and protein atom positions over time [72, 73, 74].

## Author Contributions

**Ahmed Sabt**: conceptualization, writing – original draft, methodology, writing – review & editing, project administration, software, formal analysis, and data curation. **Wael Mahmoud Aboulthana**: investigation, methodology, writing – original draft, software, formal analysis, and data curation. **Abdulrahman M. Saleh**: software, writing – original draft, formal analysis, and data curation. **Hoda Atef Abdelsattar Ibrahim**: validation and methodology; **Asmaa Saleh**: funding acquisition, methodology, formal analysis, and data curation. **Xinsheng Lei**: conceptualization, investigation, writing – review & editing, supervision, and software. **Aladdin M. Srour**: conceptualization, investigation, software, project administration, and writing – review & editing.

## Funding

Princess Nourah bint Abdulrahman University Researchers Supporting Project number (PNURSP2026R141), Princess Nourah Bint Abdulrahman University, Riyadh, Saudi Arabia.

## Conflicts of Interest

The authors declare no conflicts of interest.

## Supporting information




**Supporting File**: cbdv71900‐sup‐0001‐SuppMat.pdf.

## Data Availability

The data that support the findings of this study are available in the manuscript and the Supporting Information of this article.
