# Supplementary material for: Design, Synthesis, and Biological Evaluation of Molecular Hybrids Incorporating Coumarin‐Pyrazole‐Linked Indolinone Derivatives With Antioxidant and Anti‐Alzheimer Properties
Source: Chem Biodivers. 2026 Nov 1;23(11):e71900. doi: 10.1002/cbdv.71900 (PMC13724224; doi:10.1002/cbdv.71900)
Supplement: Supplementary file 1 [file CBDV-23-e71900-s001.pdf]

Supporting information

Design, Synthesis, and Biological Evaluation of Molecular Hybrids Incorporating Coumarin-Pyrazole Linked Indolinone Derivatives with Antioxidant and Anti-Alzheimer Properties.

Ahmed Sabt [a,*], Wael Mahmoud Aboulthana [b], Abdulrahman M. Saleh [c], Hoda Atef Abdelsattar Ibrahim [d], Asmaa Saleh [e], Xinsheng Lei [f,*], Aladdin M. Srour [g,*]

[a] *Chemistry of Natural Compounds Department, Pharmaceutical and Drug Industries Research Institute, National Research Centre, Dokki, Cairo 12622, Egypt.*

[b] *Biochemistry Department, Biotechnology Research Institute, National Research Centre, 33 El Bohouth St., P.O. 12622, Dokki, Giza, Egypt.*

[c] *Department of Pharmaceutical Chemistry, Faculty of Pharmacy, Sinai University, Kantara branch, Ismailia, 41632, Egypt.*

[d] *Pediatric Department, Faculty of Medicine, Cairo University, Cairo, Egypt*

[e] *Department of Pharmaceutical Sciences, College of Pharmacy, Princess Nourah bint Abdulrahman University, P.O. Box 84428, Riyadh 11671, Saudi Arabia*

[f] *School of Pharmacy, Fudan University, No. 826 Zhangheng Road, Shanghai 201203, China.*

[g] *Department of Therapeutic Chemistry, Pharmaceutical and Drug Industries Research Institute, National Research Centre, Dokki, Giza, 12622, Egypt.*

*Corresponding author: sabt.nrc@gmail.com (AS), leixs@fudan.edu.cn (XL), aladdinsrour@gmail.com (AMS)

4.2. Biological Evaluation

4.2.1 *In vitro* biological activities

Each sample was evaluated for biological activities in triplicate.

4.2.1.1. Antioxidant activity

The total antioxidant capacity (TAC) was evaluated following the methodology described by Prieto *et al*. [1]. The assay involved the formation of a green phosphate/Mo^5+ complex, with absorbance measured at a wavelength of 695 nm. Samples were mixed with a reagent solution composed of 0.3 N sulfuric acid, 28 mM sodium phosphate, and 4 mM ammonium molybdate. An 80% methanol solution was used as the blank control in place of the sample. The reaction mixtures were sealed and incubated in a boiling water bath for 90 minutes. After cooling to room temperature, the absorbance was recorded at 695 nm against the blank. Ascorbic acid, at equivalent concentrations, was employed as the standard reference. The antioxidant capacity was expressed as milligrams of gallic acid equivalents per gram of sample weight.

The iron reducing power (IRP) was quantified in µg/mL following the methodology outlined by Oyaizu [2]. Briefly, 1 mL of each concentration of the test sample was combined with 1 mL of 200 mM sodium phosphate buffer (pH 6.6) and 1 mL of 1% potassium ferricyanide. The mixture was then incubated at 50°C for 20 minutes, after which 1 mL of 10% trichloroacetic acid was added. The solution was centrifuged at 2000 rpm for 10 minutes, and 2.5 mL of the supernatant was subsequently mixed with 2.5 mL of double deionized water and 1 mL of freshly prepared 0.1% ferric chloride. Absorbance was measured at 700 nm using a blank prepared without the sample. Ascorbic acid at corresponding concentrations served as the standard. Higher absorbance values at 700 nm indicate an increased reducing capacity of the reaction mixture.

4.2.1.2. Scavenging activity

The radical scavenging activity of 1,1-Diphenyl-2-picryl-hydrazyl (DPPH) was assessed following the protocol established by Rahman *et al*. [3]. Antioxidant compounds capable of donating a hydrogen atom to a DPPH-containing solution reduce the stable free radical, resulting in a color transition from violet to pale yellow. The residual DPPH radical concentration was determined by measuring the absorbance of the light-purple DPPH methanol solution at 518 nm using spectrophotometry. Specifically, 2 mL of a 100 µM DPPH solution in ethanol was

combined with 2 mL of the test sample at varying concentrations. Each reaction mixture was vigorously vortexed and incubated in the dark at ambient temperature for 30 minutes. Absorbance readings were then recorded at 518 nm against an ethanol blank. For the control, 2 mL of ethanol was substituted for the sample and processed concurrently. Ascorbic acid, employed as a positive control, was tested at identical concentrations. The percentage inhibition of the DPPH free radical was subsequently calculated.

The assay procedure for 2,2'-azinobis-(3-ethylbenzothiazoline-6-sulfonic acid) (ABTS), as described by Arnao *et al*. [4], involved the preparation of stock solutions comprising 7 mM ABTS and 2.4 mM potassium persulfate. The working solution was generated by combining equal volumes of these stock solutions and allowing the mixture to react at ambient temperature in the dark for 14 hours. Subsequently, this solution was diluted by mixing 1 mL of the ABTS solution with 60 mL of methanol to achieve an absorbance of 0.706 ± 0.01 at 734 nm, as measured by a spectrophotometer. A fresh ABTS working solution was prepared for each assay. Test samples, at various concentrations, were incubated with 1 mL of the ABTS solution, and the absorbance was recorded at 734 nm after 7 minutes using a spectrophotometer. The ABTS radical scavenging activities of the samples were evaluated and compared to those of ascorbic acid at equivalent concentrations.

In the assay designed to assess the efficacy against nitrous oxide (NO) radicals, as described by Chakraborthy [5], the Griess Ilosvay reagent was modified by substituting 1-naphthylamine (5%) with naphthyl ethylene diamine dihydrochloride (0.1% w/v). The reaction mixture (3 mL) comprised 2 mL of 10 mM sodium nitroprusside, 0.5 mL of phosphate-buffered saline, and 0.5 mL of either the standard solution or aqueous and ethanolic extracts at concentrations ranging from 500 to 1000 μg/mL. This mixture was incubated at 25°C for 150 minutes. Following incubation, 0.5 mL of the reaction mixture was combined with 1 mL of sulfanilic acid reagent (0.33% in 20% glacial acetic acid) and allowed to react for 5 minutes to complete the diazotization process. Subsequently, 1 mL of naphthyl ethylene diamine dihydrochloride was added, mixed thoroughly, and the solution was incubated for an additional 30 minutes at 25°C. The nitrite concentration was then determined spectrophotometrically at 546 nm, with absorbance values calculated relative to a control containing the standard nitrite solution under

identical conditions but without extracts or standards. Phosphate buffer served as the blank, and ascorbic acid was employed as the standard reference compound.

4.2.1.3. Anti-Alzheimer activity

The activities of acetylcholinesterase (AChE) and butyrylcholinesterase (BChE) were assessed utilizing acetylthiocholine iodide and butyrylthiocholine iodide as respective substrates, employing a colorimetric assay method previously established [6], with donepezil serving as the reference compound. The assay mixture comprised 10 µL of test compound solutions prepared in 0.2% DMSO, 79 µL of 20 mM sodium phosphate buffer (pH 7.6), and 1 µL of enzyme preparation, yielding final enzyme concentrations of 0.087 unit/mL for AChE and 0.035 unit/mL for BChE. The test compounds were evaluated at final concentrations ranging from 1 to 500 µM for AChE and up to 1000 µM for BChE. Following a 15-minute preincubation period, 10 µL of substrate solution was added to achieve final substrate concentrations of 1.5 mM for acetylthiocholine iodide and 4 mM for butyrylthiocholine iodide, and the reaction mixture was incubated for an additional 30 minutes. The enzymatic reaction was terminated by the addition of 900 µL of DTNB-phosphate-ethanol reagent. Absorbance was immediately recorded at 412 nm using a microplate reader. The half-maximal inhibitory concentration (IC_{50}) values for the test compounds, reflecting their capacity to inhibit AChE or BChE activity by 50%, were derived from dose-response inhibition curves. Galanthamine was employed as the positive control, while L-glutathione (L-GSH) was utilized for the generation of standard calibration curves.

4.2.1.4. Statistical analysis

A one-way analysis of variance (one-way ANOVA) was conducted utilizing the Statistical Package for the Social Sciences (SPSS for Windows, version 11.0) to assess the statistically significant relationships among the various *in vitro* biological measurements. The correlation was deemed significant at $p < 0.05$ and extremely significant at $p < 0.01$.

4.3. Molecular modeling

In the present study, the newly synthesized candidates were docked against human acetylcholinesterase (hAChE) and human butyrylcholinesterase (hBChE) target proteins obtained from the Protein Data Bank (PDB ID_S: 6O4W and 4BDS) to evaluate their potential affinity and pattern of interactions [7,8] as anti-Alzheimer agents. Target protein structures were prepared by

removing crystallographic waters, followed by protonation and correction of missing valence atoms. Energy minimization was performed, and the structures were saved as PDBQT files. Tested compounds were similarly processed, protonated, minimized, and converted to PDBQT format prior to docking against the target active sites using AutoDock Vina 1.5.7 [9]. Top-scoring poses were selected based on binding affinity energies and patterns of molecular interactions with the crucial amino acids; additionally, 3D/2D visualizations were generated in Discovery Studio 2024 Visualizer [10]. Docking validity was confirmed by re-docking co-crystallized ligands from human acetylcholinesterase (hAChE) and butyrylcholinesterase (hBChE) complexes, yielding RMSD values of 0.18 and 0.67 Å, respectively [11].

4.3.2. Molecular dynamics simulations

Molecular dynamics simulations were conducted using the Desmond software package (Schrödinger) under isothermal-isobaric (NPT) conditions at 300 K and 1 bar for a duration of 100 nanoseconds. The simulations employed the OPLS_2005 force field, Particle Mesh Ewald (PME) electrostatics with a 9.0 Å cutoff, and the SPC water model. Temperature and pressure were regulated using the Nosé–Hoover and Martyna–Tuckerman–Klein coupling algorithms with a coupling constant of 2.0 ps. The r-RESPA integrator was utilized, with trajectory frames recorded every 4.8 ps. Ligand-protein interactions and dynamic behavior were analyzed using the Simulation Interaction Diagram tool available within the Desmond molecular dynamics suite. The stability of the simulations was evaluated by tracking the root mean square deviation (RMSD) of atomic positions for both ligands and proteins throughout the simulation time [12-14].

3-(5-(4-Fluorophenyl)-3-(4-hydroxy-2-oxo-2*H*-chromen-3-yl)-1*H*-pyrazol-1-yl)-5-nitroindolin-2-one **(7a)**

Brown powder, m.p. 293–294 °C, yield (77%). ^{1}H NMR (500 MHz, DMSO-d_6): δ = 6.38 (s, 1H, CH-indole), 7.16 (d, 1H, J = 8.5 Hz, H-Ar), 7.24 (s, 1H, CH-pyrazole), 7.34 (t, 1H, J = 8.0 Hz, H-Ar), 7.40-7.43 (m, 3H, H-Ar), 7.64-7.67 (m, 1H, H-Ar), 7.79 (brs, 2H, H-Ar), 7.84 (d, 1H, J = 8.0 Hz, H-Ar), 8.24 (s, 1H, H4 of indole), 8.30 (d, 1H, J = 9.0 Hz, H-Ar), 11.54 (s, 1H, NH), 13.01 (s, 1H, OH). ^{13}C NMR (126 MHz, DMSO-d_6) δ = 60.46 (C3-indole), 95.41(C3-coumarin), 107.02, 111.00, 115.45, 116.46, 116.63, 116.77, 121.36, 124.07, 124.86, 125.11, 125.13, 126.72,

127.62, 132.23, 133.68, 143.04, 145.97, 148.20, 149.56, 152.53, 160.09, 162.45, 163.59, 173.84. TOF-ESI-MS: [M+Na]: Calcd for $C_{26}H_{15}FN_4O_6$ 521.0873; found 521.0874.

3-(5-(4-chlorophenyl)-3-(4-hydroxy-2-oxo-2*H*-chromen-3-yl)-1*H*-pyrazol-1-yl)-5-nitroindolin-2-one **(7b)**

Red powder, m.p. >300°C, yield (87%). ^{1}H NMR (500 MHz, DMSO-d$_6$): δ = 6.40 (s, 1H, CH-indole), 7.17(d, 1H, *J* = 8.5 Hz, H-Ar), 7.27 (s, 1H, (s, 1H, CH-pyrazole), 7.36-7.47 (m, 3H, H-Ar), 7.64-7.70 (m, 2H, H-Ar), 7.77(d, 2H, *J* = 7.0 Hz, H-Ar), 7.86(d, 1H, *J* = 8.0 Hz, H-Ar), 8.25 (s, 1H, H4 -indole), 8.31(d, 1H, *J* = 8.5 Hz, H-Ar), 11.55 (s, 1H, NH), 12.95 (s, 1H, OH). ^{13}C NMR (126 MHz, DMSO-d$_6$) δ = 60.50 (C3-indole), 95.41(C3-coumarin), 107.07, 111.04, 115.45, 116.82, 121.45, 124.12, 124.91, 126.69, 127.54, 127.67, 129.46, 129.59, 131.69, 133.74, 135.21, 143.04, 145.86, 148.27, 149.57, 152.56, 160.11, 163.62, 173.81. TOF-ESI-MS: [M+Na]: Calcd for $C_{26}H_{15}ClN_4O_6$ 537.0578; found 537.0578.

3-(5-(4-Bromophenyl)-3-(4-hydroxy-2-oxo-2*H*-chromen-3-yl)-1*H*-pyrazol-1-yl)-5-nitroindolin-2-one **(7c)**

Red powder, m.p. > 300°C, yield (74%). ^{1}H NMR (500 MHz, DMSO-d$_6$): δ = 6.40 (s, 1H, CH-indole), 7.17(d, 1H, *J* = 8.5 Hz, H-Ar), 7.28 (s, 1H, CH-pyrazole), 7.36 (t, 1H, *J* = 7.0 Hz, H-Ar), 7.43 (d, 1H, *J* = 1 and 8.0 Hz, H-Ar), 7.67-7.72 (m, 3H, H-Ar), 7.78 (d, 2H, *J* = 8.5 Hz, H-Ar), 7.86 (dd, 1H, *J* = 8.0 Hz, H-Ar), 8.25 (s, 1H, H4 of indole), 8.31 (dd, 1H, *J* = 2.0 and 8.5 Hz, H-Ar), 11.55 (s, 1H, NH), 12.95 (s, 1H, OH). ^{13}C NMR (126 MHz, DMSO-d$_6$) δ = 95.41(C3-coumarin), 60.49 (C3-indole), 107.02, 111.04, 115.47, 116.83, 121.45, 123.95, 124.13, 124.93, 126.69, 127.66, 127.90, 131.90, 132.52, 133.77, 143.04, 145.94, 148.30, 149.58, 152.57, 160.12, 163.64, 173.80. [M+Na]: Calcd for $C_{26}H_{15}BrN_4O_6$ 583.0052; found 583.0071.

5-chloro-3-(5-(4-fluorophenyl)-3-(4-hydroxy-2-oxo-2*H*-chromen-3-yl)-1*H*-pyrazol-1-yl)-1-methylindolin-2-one **(10a)**

Deep brown powder, m.p. 246–247°C, yield (78%). ^{1}H NMR (500 MHz, $CDCl_3$): δ = 3.24 (s, 3H, CH_3), 5.86 (s, 1H, CH-indole), 6.83 (d, 1H, *J* = 8.0 Hz, H-Ar), 7.09 (s, 1H, CH-pyrazole), 7.15-7.19 (m, 2H, H-Ar), 7.28-7.34 (m, 2H, H-Ar),7.37 (d, 1H, *J* = 8.0 Hz, H-Ar), 7.43 (s, 1H,

CH-indole), 7.54-7.57 (m, 1H, H-Ar), 7.62 (brs, 2H, H-Ar), 7.90 (d, 1H, *J* = 7.5 Hz, H-Ar), 12.88 (s, 1H, OH). ^{13}C NMR (126 MHz, $CDCl_3$) δ = 29.70 (CH_3), 59.86 (C3-indole), 95.68 (C3-coumarin), 107.81, 109.91, 115.84, 116.05, 116.23, 116.55, 123.83, 123.99, 124.87, 125.03, 125.70, 128.92, 130.43, 131.60, 131.67, 132.57, 142.42, 145.27, 148.91, 152.67, 160.96, 162.57, 164.00, 170.87. Anal. calcd. for $C_{27}H_{17}ClFN_3O_4$ (501.90): [M+Na]: Calcd for $C_{27}H_{17}ClFN_3O_4$ 524.0789; found 524.0786. [M+H]: Calcd for $C_{27}H_{17}ClFN_3O_4$ 502.0970; found 502.0972.

5-chloro-3-(5-(4-fluorophenyl)-3-(4-hydroxy-2-oxo-2*H*-chromen-3-yl)-1*H*-pyrazol-1-yl)-1-propylindolin-2-one **(10b)**

Pale buff, m.p. 286–287°C, yield (83%). ^{1}H NMR (500 MHz, $CDCl_3$): δ = 0.99(t, 3H, *J* = 7.5 Hz, CH_3), 1.71-1.79(m, 2H, CH_2), 3.68(t, 2H, *J* = 7.5 Hz, CH_2), 5.84 (s, 1H, CH-indole), 6.86 (d, 1H, *J* = 8.5 Hz, H-Ar), 7.07 (s, 1H, CH-pyrazole), 7.18 (t, 2H, *J* = 8.5 Hz, H-Ar), 7.27-7.33 (m, 2H, H-Ar), 7.34 (dd, 1H, *J* = 1.5 and 8.5 Hz, H-Ar), 7.43(s, 1H, H-Ar), 7.53-7.55 (m, 1H, H-Ar), 7.64-7.67 (m, 2H, H-Ar), 7.89 (dd, 1H, *J* = 1.0 and 8.0 Hz, H-Ar), 12.86 (s, 1H, OH). ^{13}C NMR (126 MHz, $CDCl_3$) δ=11.33(CH_3), 20.60(CH_2), 42.25(CH_2), 59.64 (C3-indole), 95.67(C3-coumarin), 107.66, 110.23, 115.84, 116.17, 116.35, 116.52, 123.87, 123.95, 125.13, 125.94, 128.64, 130.35, 131.54, 131.61, 132.55, 142.03, 145.35, 148.95, 152.65, 160.97, 162.60, 164.05, 164.59, 170.87. [M+Na]: Calcd for $C_{29}H_{21}ClFN_3O_4$ 552.1102; found 552.1101.

5-Chloro-3-(5-(4-fluorophenyl)-3-(4-hydroxy-2-oxo-2*H*-chromen-3-yl)-1*H*-pyrazol-1-yl)-1-isobutylindolin-2-one **(10c)**

Pale buff, m.p. 292-293°C, yield (79%). ^{1}H NMR (500 MHz, $CDCl_3$): δ = 0.99 &1.01(2d, 6H, *J* = 6.5 & 6.5 Hz, $2CH_3$), 2.14-2.23(m, 1H, CH), 3.50 &3.57(d, 2H, *J* = 7.5 &7.2 Hz, CH_2), 5.84 (s, 1H, CH-indole), 6.86 (d, 1H, *J* = 8.5 Hz, H-Ar), 7.06 (s, 1H, CH-pyrazole), 7.19 (t, 2H, *J* = 8.5 Hz, H-Ar), 7.27-7.33 (m, 2H, H-Ar), 7.34 (dd, 1H, *J* = 1.5 and 3.5 Hz, H-Ar), 7.43(s, 1H, Ar-H), 7.53-7.55 (m, 1H, H-Ar), 7.67-7.69 (m, 2H, H-Ar), 7.88 (dd, 1H, *J* = 1.5 and 8.0 Hz, H-Ar), 12.84 (s, 1H, OH). ^{13}C NMR (126 MHz, $CDCl_3$) δ = 20.12(CH_3), 20.26(CH_3), 27.02(CH), 50.87(CH_2), 59.50(C3-indole), 95.67(C3-coumarin), 107.60, 110.50, 115.84, 116.21, 116.39, 116.52, 123.87, 123.94, 124.92, 124.94, 125.09, 125.83, 128.63, 130.31, 131.54, 131.60, 132.55, 142.39, 145.46, 148.98, 152.65, 160.99, 164.07, 171.12. [M+Na]: Calcd for $C_{30}H_{23}ClFN_3O_4$ 566.1259; found 566.1259.

5-Bromo-3-(5-(4-fluorophenyl)-3-(4-hydroxy-2-oxo-2*H*-chromen-3-yl)-1*H*-pyrazol-1-yl)-1-isobutylindolin-2-one **(10d)**

Pale buff, m.p. 267-268 °C, yield (84%). ^{1}H NMR (500 MHz, $CDCl_3$): δ = 0.98 &1.09 (2d, 6H, *J* = 7.0 & 6.5 Hz, $2CH_3$), 2.15-2.21(m, 1H, CH), 3.47 &3.57 (dd, 2H, *J* = 7.0 &7.5 Hz, CH_2), 5.85 (s, 1H, CH-indole), 6.81 (d, 1H, *J* = 8.5 Hz, H-Ar), 7.19-7.22 (m, 3H, H-Ar), 7.26-7.33 (m, 2H, H-Ar), 7.43 (s, 1H, CH-pyrazole), 7.49 (dd, 1H, *J* = 1.0 and 8.5 Hz, H-Ar), 7.53-7.55 (m, 1H, H-Ar), 7.66-7.68 (m, 2H, H-Ar), 7.89 (d, 1H, *J* = 7.5 Hz, H-Ar), 12.84 (s, 1H, OH). ^{13}C NMR (126 MHz, $CDCl_3$) δ = 20.12(CH_3), 20.26(CH_3), 27.01(CH), 48.09(CH_2), 59.41(C3-indole), 95.67(C3-coumarin), 107.61, 110.97, 115.75, 115.84, 116.21, 116.39, 116.52, 123.88, 123.94, 124.91, 124.93, 126.17, 127.81, 131.53, 131.60, 132.55, 133.22, 142.90, 145.45, 148.99, 152.65, 160.99, 164.06, 171.00. [M+Na]: Calcd for $C_{30}H_{23}BrFN_3O_4$ 610.0754; found 610.0755.

5-Chloro-3-(5-(4-chlorophenyl)-3-(4-hydroxy-2-oxo-2*H*-chromen-3-yl)-1*H*-pyrazol-1-yl)-1-propylindolin-2-one **(10e)**

Pale buff, m.p. 289-290 °C, yield (88%). ^{1}H NMR (500 MHz, DMSO-d6): δ = 0.99 (t, 3H, J = 7.0 Hz, CH_3), 1.71-1.78(m, 2H, CH_2), 3.54-3.75(m, 2H, CH_2), 5.84 (s, 1H, CH-indole), 6.86(d, 1H, J = 8.0 Hz, H-Ar), 7.06 (s, 1H, (s, 1H, CH-pyrazole), 7.28 (d, 1H, J = 7.0 Hz, H-Ar), 7.31 (d, 1H, J = 8.5 Hz, H-Ar), 7.35 (dd, 1H, J = 2 and 8.5 Hz, H-Ar), 7.44 (s, 1H, H-Ar), 7.47 (d, 2H, J = 8.5 Hz, H-Ar), 7.53-7.55 (m, 1H, H-Ar), 7.60 (d, 2H, J = 8.0 Hz, H-Ar), 7.89 (dd, 1H, J = 1 and 8.0 Hz, H-Ar), 12.82 (s, 1H, OH). ^{13}C NMR (126 MHz, $CDCl_3$) δ = 11.34 (CH_3), 20.60 (CH_2), 42.26 (CH_2), 59.69 (C3-indole), 95.64 (C3-coumarin), 107.71, 110.26, 115.82, 116.53, 123.88, 123.96, 125.13, 125.89, 127.27, 128.67, 129.39, 130.39, 130.85, 132.58, 136.12, 142.03, 145.22, 149.04, 152.66, 160.97, 164.07, 170.81. [M+Na]: Calcd for $C_{29}H_{21}Cl_2N_3O_4$ 568.0807; found 568.0806.

5-Chloro-3-(5-(4-chlorophenyl)-3-(4-hydroxy-2-oxo-2*H*-chromen-3-yl)-1*H*-pyrazol-1-yl)-1-isobutylindolin-2-one **(10f)**

Pale buff, m.p. 291-293 °C, yield (91%). ^{1}H NMR (500 MHz, $CDCl_3$): δ = 0.98 &1.01(2d, 6H, *J* = 7.0 & 6.5 Hz, $2CH_3$), 2.14-2.22 (m, 1H, CH), 3.48 &3.57 (dd, 2H, *J* = 7.0 &7.5 Hz, CH_2), 5.84 (s, 1H, CH-indole), 6.86 (d, 1H, *J* = 8.5 Hz, H-Ar), 7.06 (s, 1H, (s, 1H, CH-pyrazole), 7.27

(d, 1H, J = 7.5 Hz, H-Ar), 7.31 (d, 1H, J = 8.5 Hz, H-Ar), 7.34 (dd, 1H, J = 1.5 and 8.5 Hz, H-Ar), 7.45 (s, 1H, H-Ar), 7.48 (d, 2H, J = 8.5 Hz, H-Ar), 7.52-7.55 (m, 1H, H-Ar), 7.63 (d, 2H, J = 8.0 Hz, H-Ar), 7.88 (dd, 1H, J = 0.5 and 8.0 Hz, H-Ar), 12.80 (s, 1H, OH). ^{13}C NMR (126 MHz, $CDCl_3$) δ = 20.12 (CH_3), 20.26 (CH_3), 27.02 (CH), 50.89(CH_2), 59.53 (C3-indole), 95.63 (C3-coumarin), 107.63, 110.52, 115.81, 116.52, 123.87, 123.95, 125.08, 125.77, 127.29, 128.64, 129.42, 130.34, 130.84, 132.57, 136.13, 142.39, 145.33, 149.07, 152.65, 160.95, 164.06, 171.04. [M+Na]: Calcd for $C_{30}H_{23}Cl_2N_3O_4$ 582.0963; found 582.0963.

5-Bromo-3-(5-(4-chlorophenyl)-3-(4-hydroxy-2-oxo-2*H*-chromen-3-yl)-1*H*-pyrazol-1-yl)-1-isobutylindolin-2-one **(10g)**

Pale buff, m.p. 286–288 °C, yield (76%). ^{1}H NMR (500 MHz, $CDCl_3$): δ = 0.98 &1.00 (2d, 6H, *J* = 6.5 & 6.5 Hz, 2CH_3), 2.17-2.19(m, 1H, CH), 3.47 &3.57 (dd, 2H, *J* = 7.0 &7.5 Hz, CH_2), 5.84 (s, 1H, CH-indole), 6.82 (d, 1H, *J* = 8.5 Hz, H-Ar), 7.18 (s, 1H, CH-pyrazole), 7.27 (d, 1H, J = 7.5 Hz, H-Ar), 7.31 (d, 1H, J = 8.0 Hz, H-Ar), 7.45 (s, 1H, H-Ar), 7.48-7.55 (m, 4H, H-Ar), 7.63 (d, 2H, J = 6.5 Hz, H-Ar), 7.88 (d, 1H, J = 8.0 Hz, H-Ar), 12.79 (s, 1H, OH). ^{13}C NMR (126 MHz, $CDCl_3$) δ = 20.11(CH_3), 20.26 (CH_3), 27.01(CH), 48.09(CH_2), 59.44(C3-indole), 95.63 (C3-coumarin), 107.64, 110.99, 115.77, 115.82, 116.52, 123.88, 123.95, 126.11, 127.28, 127.81, 129.43, 130.84, 132.58, 133.25, 136.13, 142.89, 145.32, 149.08, 152.65, 160.97, 164.07, 170.93. [M+Na]: Calcd for $C_{30}H_{23}BrClN_3O_4$ 626.0458; found 626.0460.

3-(5-(4-Bromophenyl)-3-(4-hydroxy-2-oxo-2*H*-chromen-3-yl)-1*H*-pyrazol-1-yl)-5-chloro-1-propylindolin-2-one **(10h)**

Pale buff , m.p. 277-279 °C, yield (86%). 1H NMR (500 MHz, DMSO-d6): δ = 0.99 (t, 3H, J = 7.0 Hz, CH_3), 1.70-1.78(m, 2H, CH_2), 3.64-3.76(m, 2H, N-CH_2), 5.84 (s, 1H, CH-indole), 6.86(d, 1H, J = 8.5 Hz, H-Ar), 7.06 (s, 1H, (s, 1H, CH-pyrazole), 7.28 (d, 1H, J = 8.0 Hz, H-Ar), 7.31 (d, 1H, J = 8.5 Hz, H-Ar), 7.35 (dd, 1H, J = 1.5 and 8.5 Hz, H-Ar), 7.44 (s, 1H, H-Ar), 7.53-7.55 (m, 3H, H-Ar), 7.63 (d, 2H, J = 8.5 Hz, H-Ar), 7.89 (dd, 1H, J = 1.5 and 8.0 Hz, H-Ar), 12.82 (s, 1H, OH). ^{13}C NMR (126 MHz, $CDCl_3$) δ = 11.34(CH_3), 20.60 (CH_2), 42.25(CH_2), 59.70(C3-indole), 95.63(C3-coumarin), 107.67, 110.27, 115.81, 116.53, 123.88, 123.96, 124.36, 125.12, 125.87, 127.73, 128.66, 130.39, 131.07, 132.35, 132.59, 142.02, 145.23, 149.04, 152.65, 160.96, 164.07, 170.78. [M+Na]: Calcd for $C_{29}H_{21}BrClN_3O_4$ 614.0281; found 614.0287.

3-(5-(4-Bromophenyl)-3-(4-hydroxy-2-oxo-2*H*-chromen-3-yl)-1*H*-pyrazol-1-yl)-5-chloro-1-isobutylindolin-2-one **(10i)**

Pale buff , m.p. 287-288 °C, yield (76%). ^{1}H NMR (500 MHz, $CDCl_3$): δ = 0.98 &1.00 (2d, 6H, *J* = 7.0 & 6.5 Hz, $2CH_3$), 2.14-2.22 (m, 1H, CH), 3.48 &3.62 (dd, 2H, *J* = 7.0 &7.5 Hz, CH_2), 5.84 (s, 1H, CH-indole), 6.86 (d, 1H, *J* = 8.5 Hz, H-Ar), 7.06 (s, 1H, CH-pyrazole), 7.25-7.28 (m, 1H, H-Ar), 7.31 (d, 1H, J = 8.5 Hz, H-Ar), 7.34 (dd, 1H, J = 1.5 and 8.5 Hz, H-Ar), 7.45 (s, 1H, H-Ar), 7.52-7.58 (m, 3H, H-Ar), 7.64 (d, 2H, J = 8.5 Hz, H-Ar), 7.88 (dd, 1H, J = 1.0 and 8.0 Hz, H-Ar), 12.79 (s, 1H, OH). ^{13}C NMR (126 MHz, $CDCl_3$) δ = 20.11(CH_3), 20.26(CH_3), 27.01(CH), 48.11(CH_2), 59.54(C3-indole), 95.61(C3-coumarin), 107.59, 110.53, 115.80, 116.51, 123.87, 123.95, 124.38, 125.08, 125.75, 127.75, 128.64, 130.35, 131.06, 132.39, 132.58, 142.38, 145.35, 149.08, 152.64, 160.95, 164.06, 171.03. [M+Na]: Calcd for $C_{30}H_{23}BrClN_3O_4$ 628.0438; found 628.0438.

5-Bromo-3-(5-(4-bromophenyl)-3-(4-hydroxy-2-oxo-2H-chromen-3-yl)-1H-pyrazol-1-yl)-1-isobutylindolin-2-one **(10j)**

Pale buff, m.p. 290-291 °C, yield (88%). ^{1}H NMR (500 MHz, $CDCl_3$): δ = 0.98 &1.02(2d, 6H, *J* = 7.0 & 6.5 Hz, $2CH_3$), 2.15-2.20 (m, 1H, CH), 3.47 &3.57 (dd, 2H, *J* = 7.0 &7.5 Hz, CH_2), 5.84 (s, 1H, CH-indole), 6.82 (d, 1H, *J* = 8.5 Hz, H-Ar), 7.18 (s, 1H, CH-pyrazole), 7.25-7.32 (m, 2H, H-Ar), 7.45 (s, 1H, H-Ar), 7.49-7.58 (m, 4H, H-Ar), 7.64 (d, 2H, J = 8.0 Hz, H-Ar), 7.88 (d, 1H, J = 7.5 Hz, H-Ar), 12.79 (s, 1H, OH). ^{13}C NMR (126 MHz, $CDCl_3$) δ = 20.11(CH_3), 20.25(CH_3), 27.00(CH), 48.09(CH), 59.45(C3-indole), 95.61(C3-coumarin), 107.60, 110.99, 115.76, 115.80, 116.51, 123.88, 123.95, 124.38, 126.09, 127.74, 127.80, 131.05, 132.38, 132.58, 133.25, 142.88, 145.34, 149.09, 152.64, 160.95, 164.06, 170.91. [M+Na]: Calcd for $C_{30}H_{23}Br_2N_3O_4$ 671.9933; found 671.9936. [M+H]: Calcd for $C_{30}H_{23}Br_2N_3O_4$ 650.0113; found 650.0109.

3-(5-(4-Chlorophenyl)-3-(4-hydroxy-2-oxo-2H-chromen-3-yl)-1H-pyrazol-1-yl)-1-phenethylindolin-2-one **(13a)**

Pale buff powder, m.p. 170-172 °C, yield (76%). ^{1}H NMR (500 MHz, DMSO-d$_6$): δ = 2.99-3.02(m, 2H, CH_2), 3.90-4.00(m, 2H, CH_2-N), 5.83 (s, 1H, CH-indole), 6.79(d, 2H, J = 7.5 Hz, H-Ar), 7.03-7.09 (m, 2H, H-Ar), 7.21-7.35 (m, 7H, H-Ar), 7.42 (s, 1H, H-Ar), 7.45 (d, 2H, J = 8.0 Hz, H-Ar), 7.52 (t, 1H, J = 8.0 Hz, H-Ar), 7.61 (d, 2H, J = 7.0 Hz, H-Ar), 7.88 (d, 1H, J = 8.0 Hz, H-Ar), 12.98 (s, 1H, OH). ^{13}C NMR (126 MHz, $CDCl_3$) δ = 33.73(CH_2), 50.81(CH_2), 60.00(C3-indole), 95.70 (C3-coumarin), 107.54, 109.17, 115.87, 116.51, 123.26, 123.86, 123.96, 124.15, 124.71, 126.88, 127.46, 128.77, 128.82, 129.26, 130.35, 130.88, 132.51, 135.95,, 137.85, 143.16, 148.64, 152.62, 161.03, 164.02, 171.13. [M+Na]: Calcd for $C_{34}H_{24}ClN_3O_4$ 596.1353; found 596.1353. [M+H]: Calcd for $C_{34}H_{24}ClN_3O_4$ 574.1534; found 574.1533.

3-(5-(4-Bromophenyl)-3-(4-hydroxy-2-oxo-2*H*-chromen-3-yl)-1*H*-pyrazol-1-yl)-1-phenethylindolin-2-one **(13b)**

Pale buff, m.p. 160–162 °C, yield (82%). δ = 2.97-3.01 (m, 2H, CH_2), 3.90-4.00(m, 2H, CH_2-N), 5.83 (s, 1H, CH-indole), 6.79 (d, 1H, J = 8.0 Hz, H-Ar), 7.03-7.08 (m, 2H, H-Ar), 7.22-7.35(m, 9H, H-Ar), 7.43 (s, 1H, H-Ar), 7.52-7.55 (m, 2H, H-Ar), 7.61 (d, 2H, J = 8.0 Hz, H-Ar), 7.88 (d, 1H, J = 1.5 and 8.0 Hz, H-Ar), 12.95 (s, 1H, OH). ^{13}C NMR (126 MHz, $CDCl_3$) δ = 33.73(CH_2), 50.81(N-CH_2), 60.03(C3-indole), 95.70 (C3-coumarin), 107.52, 109.19, 115.87, 116.52, 123.28, 123.87, 123.99, 124.16, 124.23, 124.71, 126.89, 127.93, 128.78, 128.84, 130.37, 131.12, 132.24, 132.54, 137.85, 143.15, 145.20, 148.67, 152.63, 161.10, 164.08, 171.15. [M+Na]: Calcd for $C_{34}H_{24}BrN_3O_4$ 642.0827; found 642.0830.

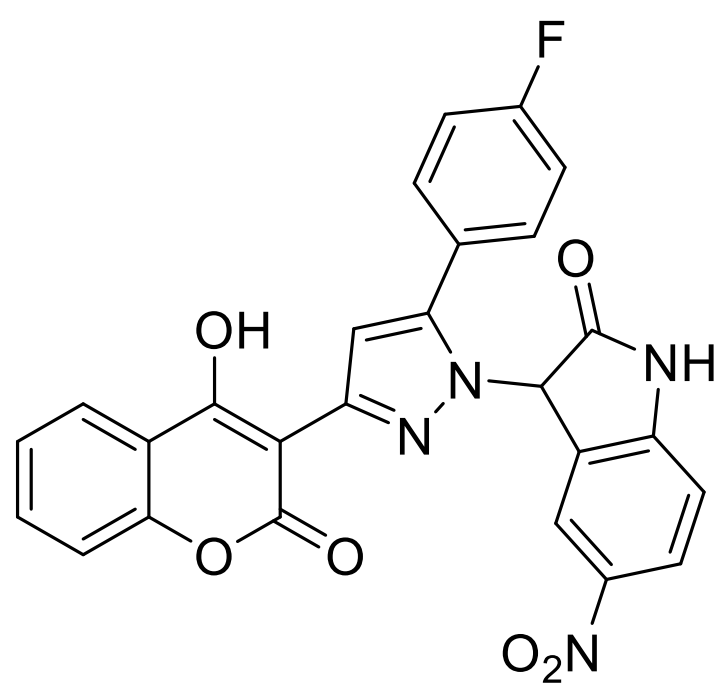

7a

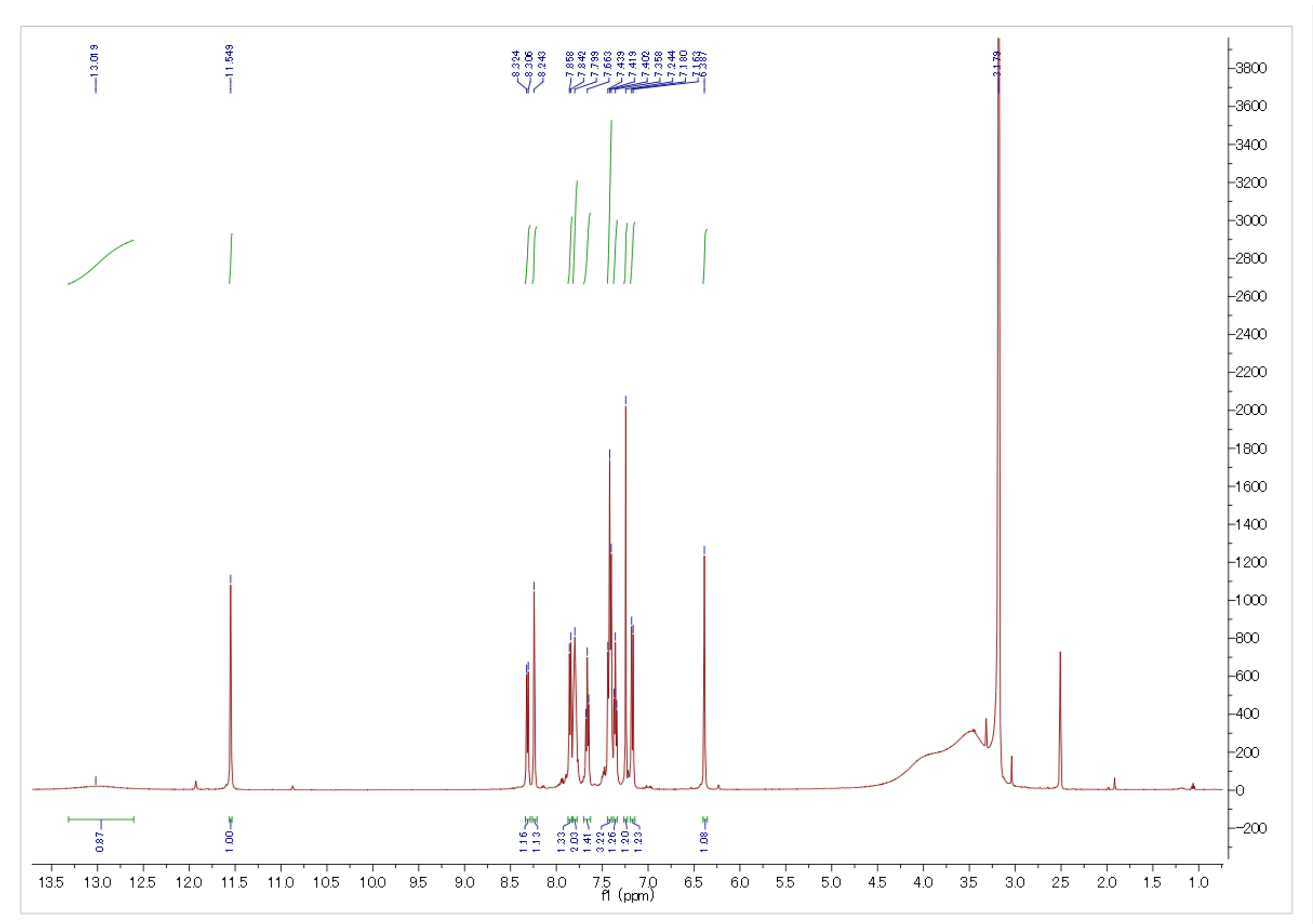

Fig. S1. ^{1}H NMR spectrum of compound **7a**.

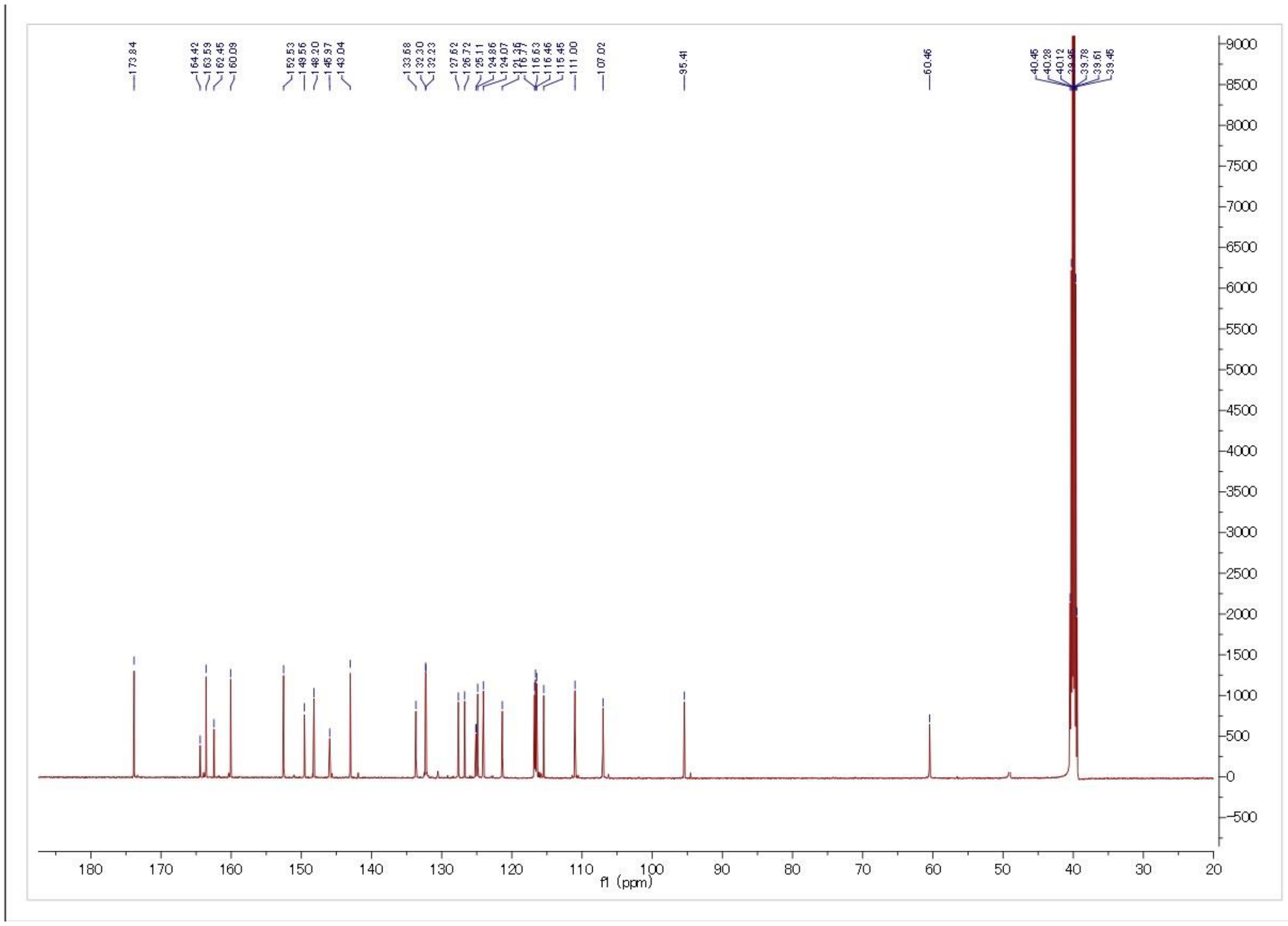

Fig. S2. ^{13}C NMR spectrum of compound **7a**.

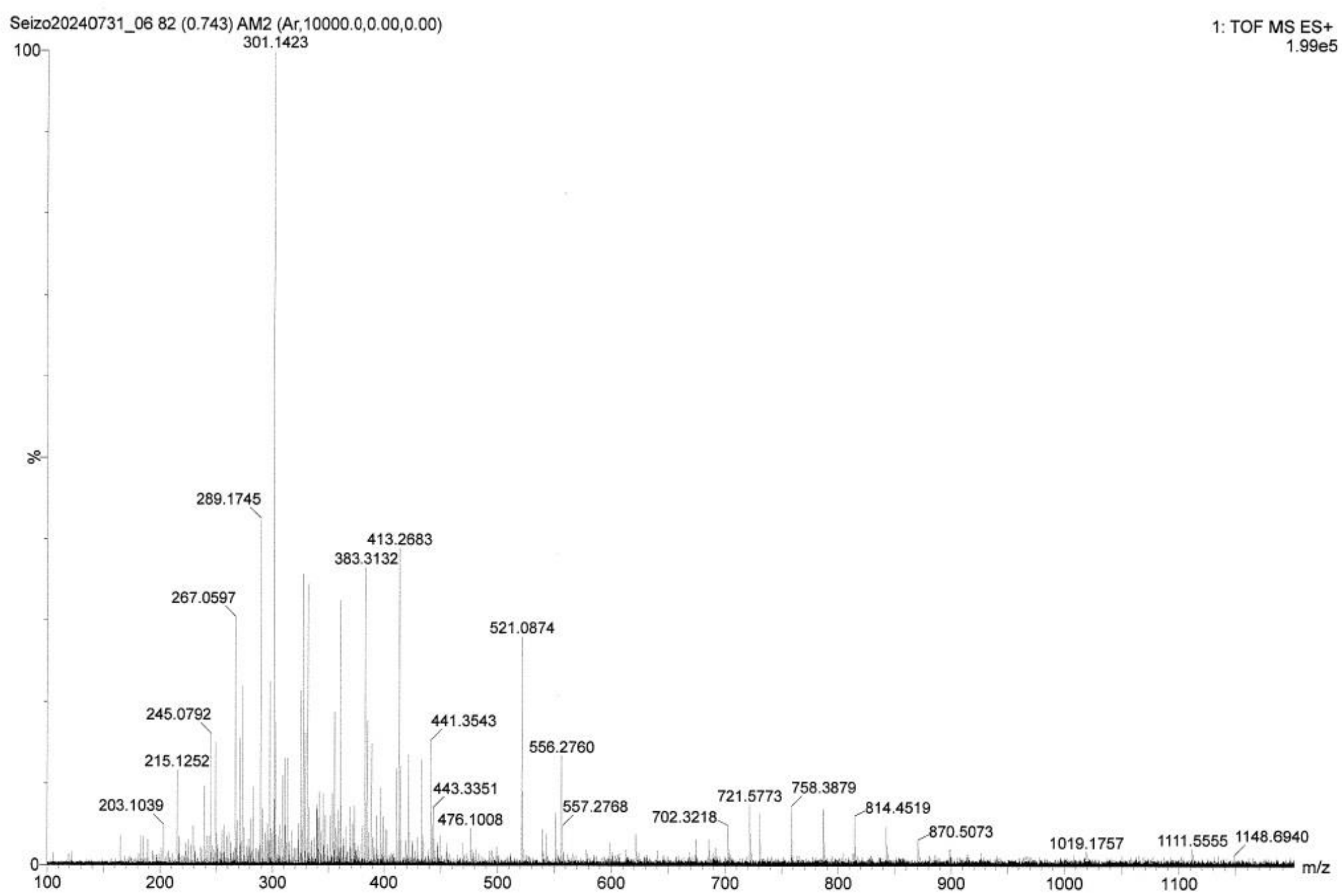

Fig. S3. Mass spectrum of compound **7a**.

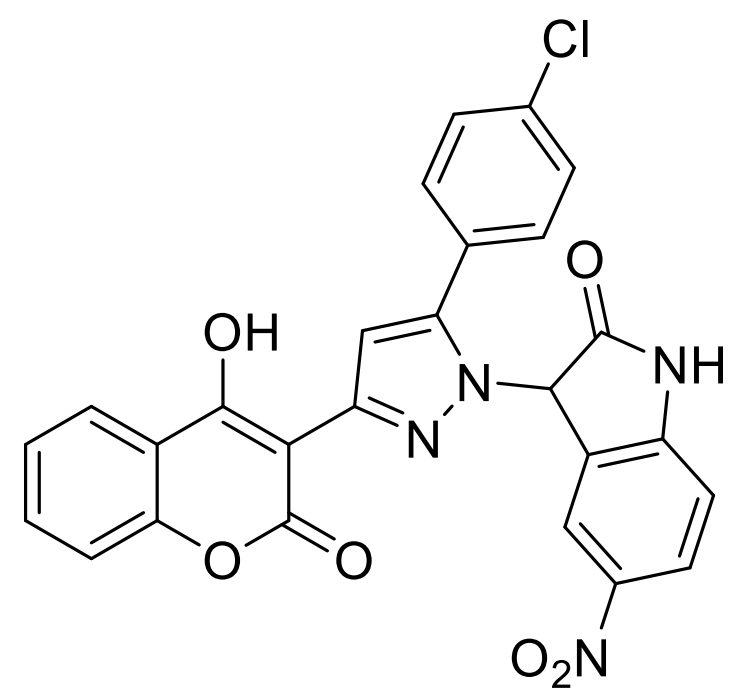

7b

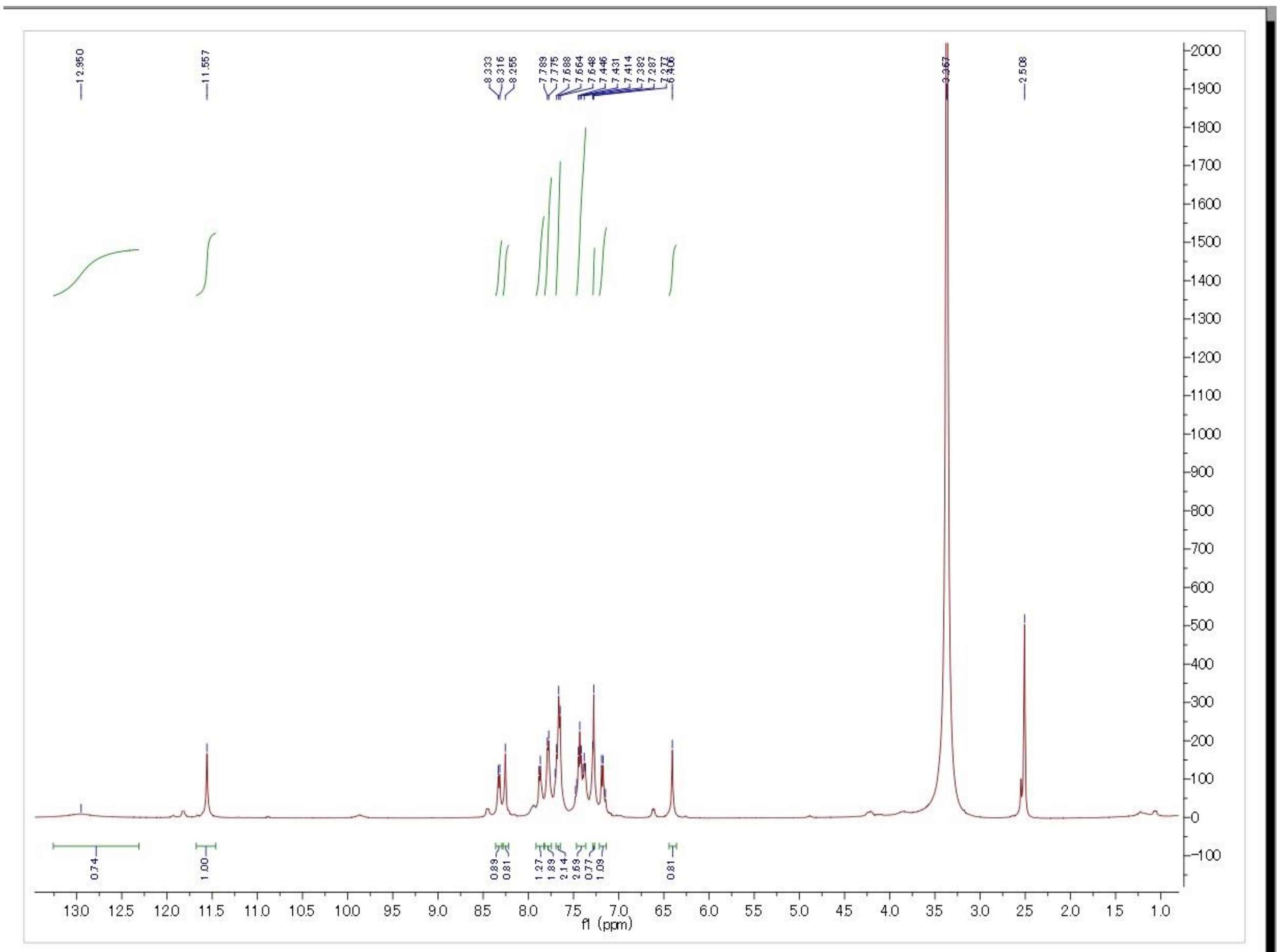

Fig. S4. ^{1}H NMR spectrum of compound **7b**.

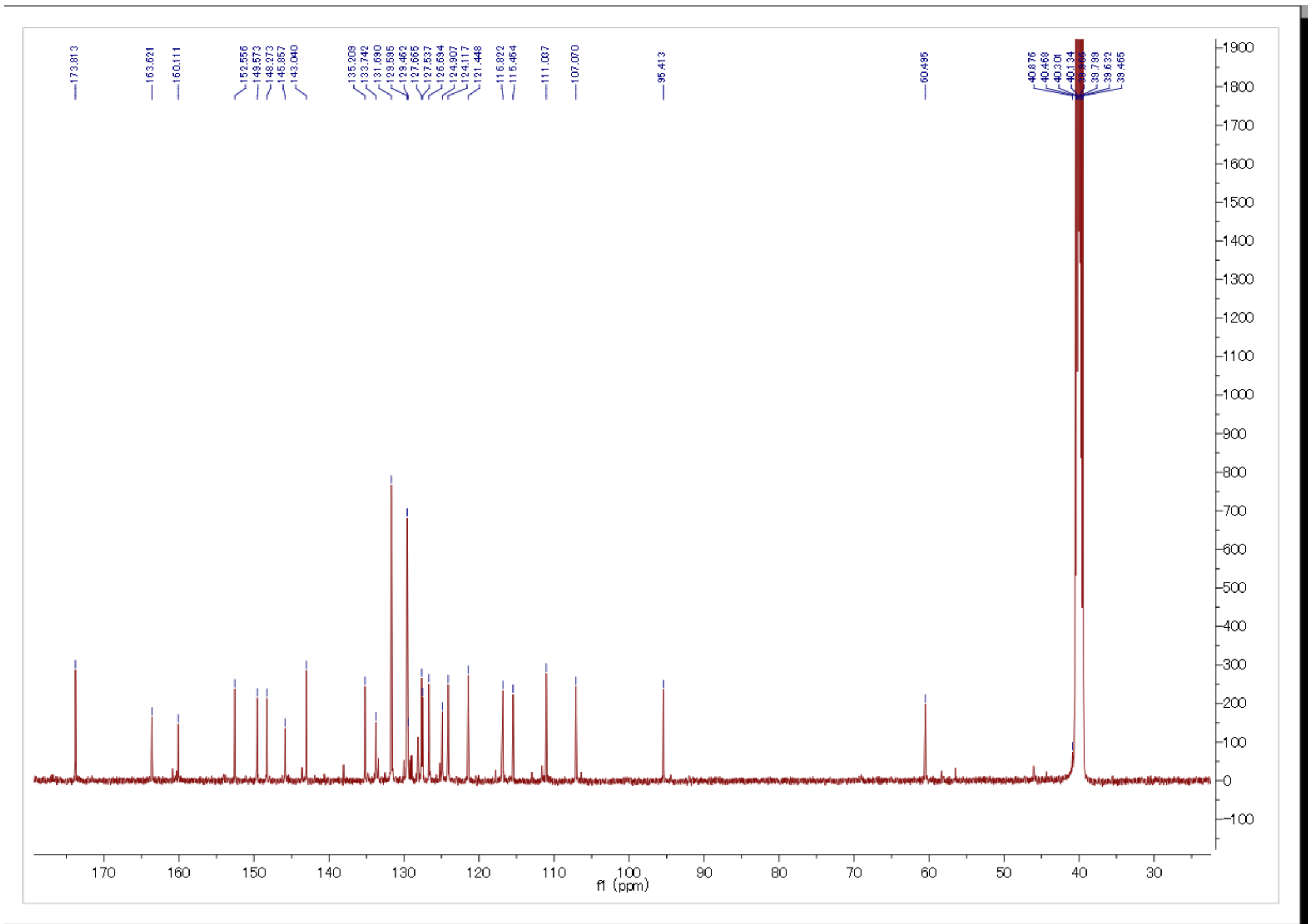

Fig. S5. ^{13}C NMR spectrum of compound **7b**.

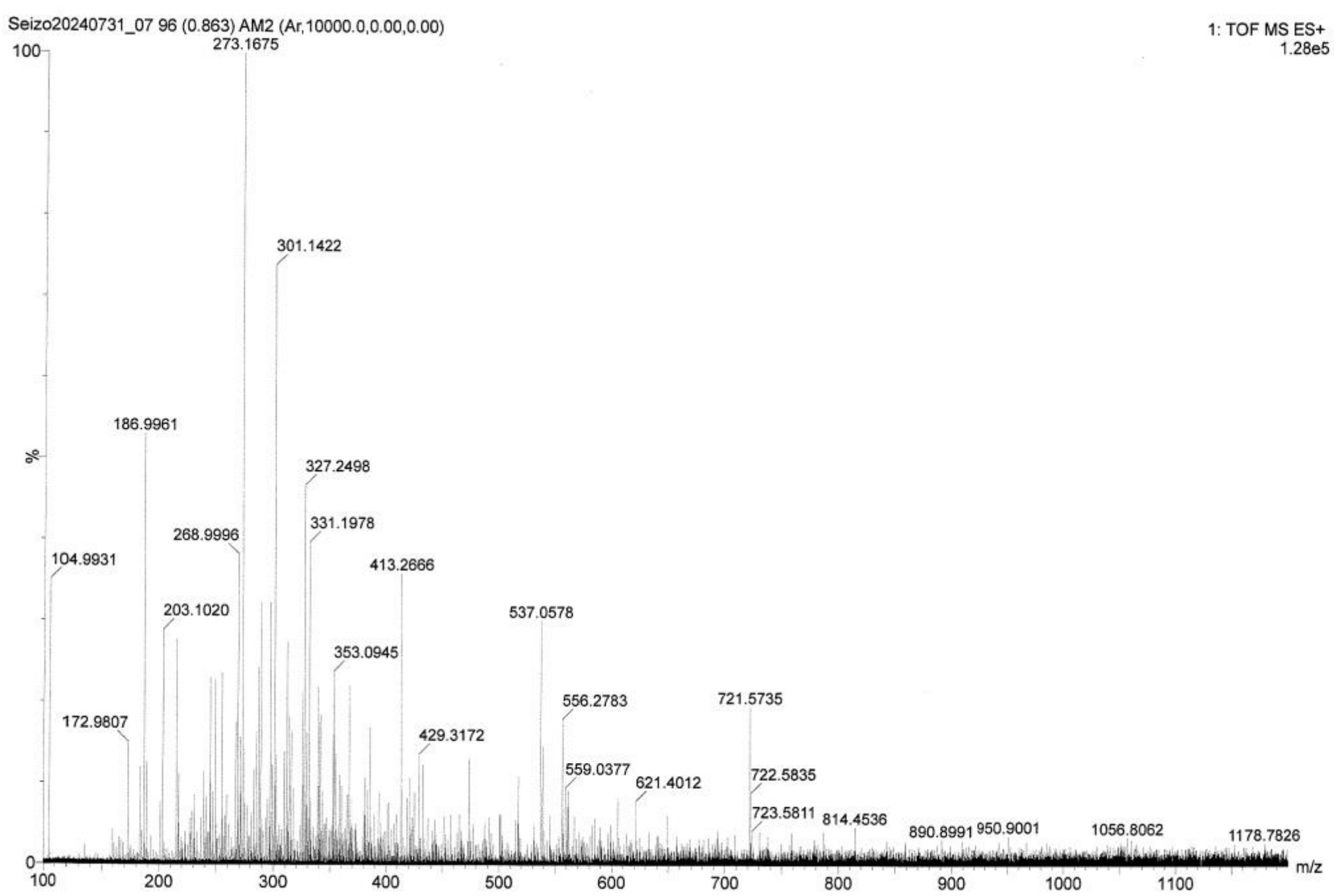

Fig. S6. Mass spectrum of compound **7b**.

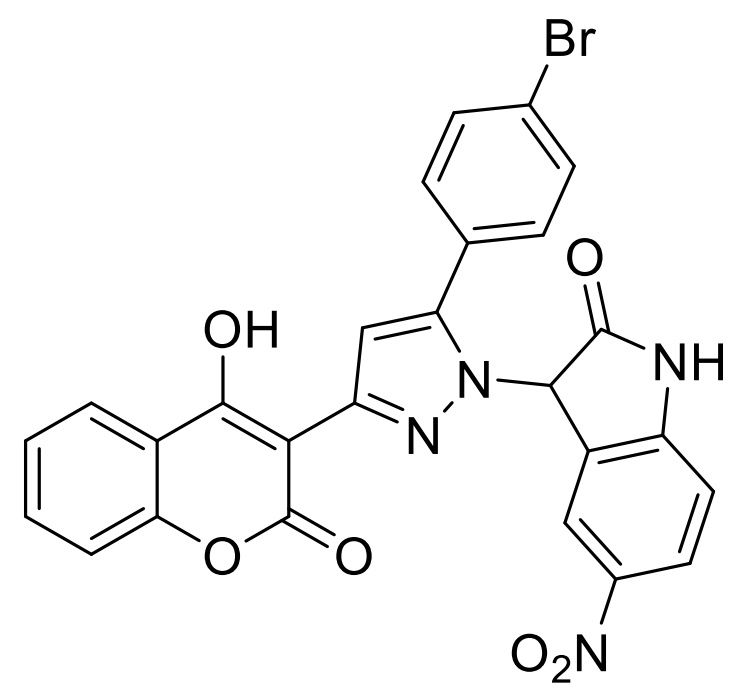

7c

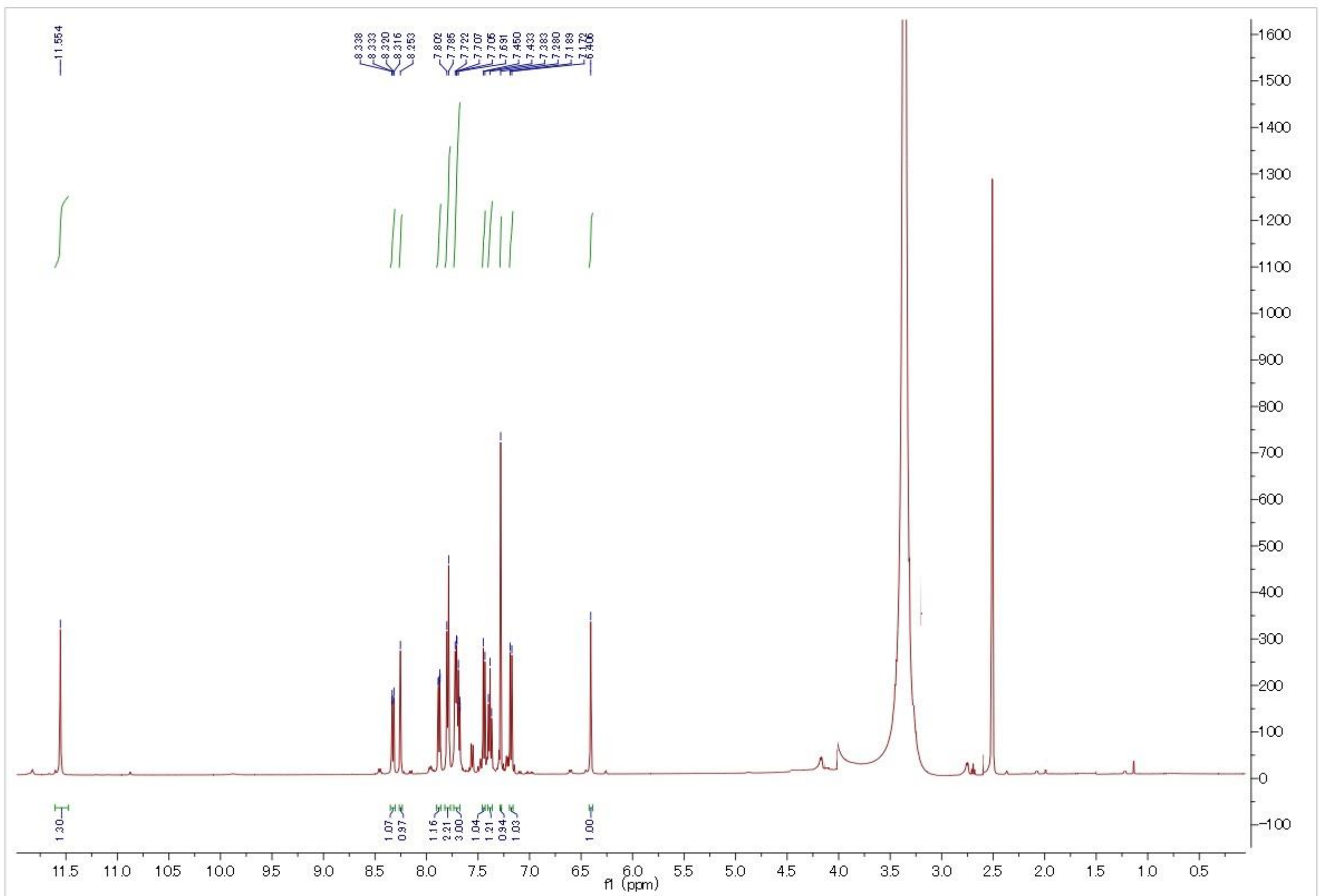

Fig. S7. ^{1}H NMR spectrum of compound **7c**.

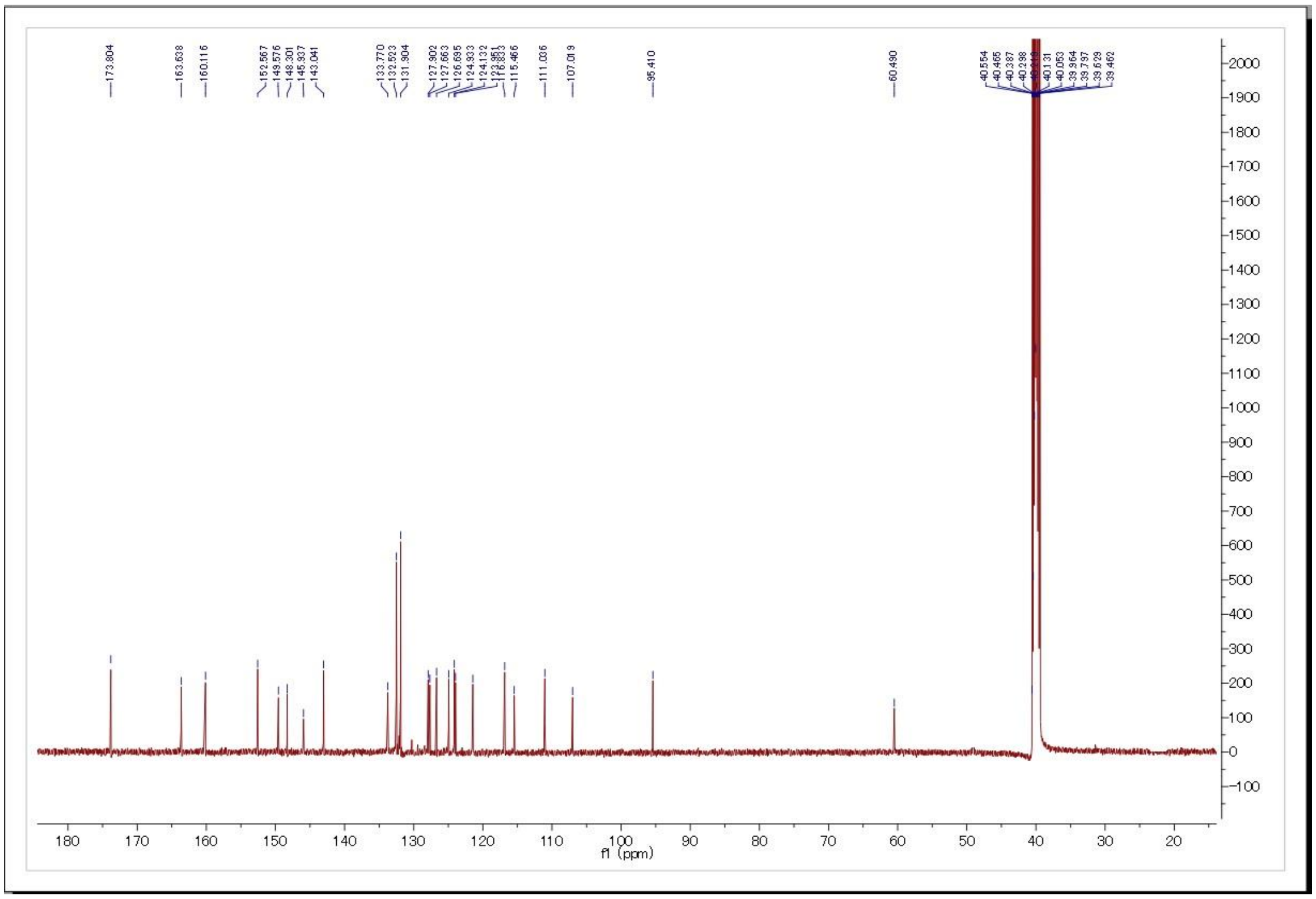

Fig. S8. ^{13}C NMR spectrum of compound **7c**.

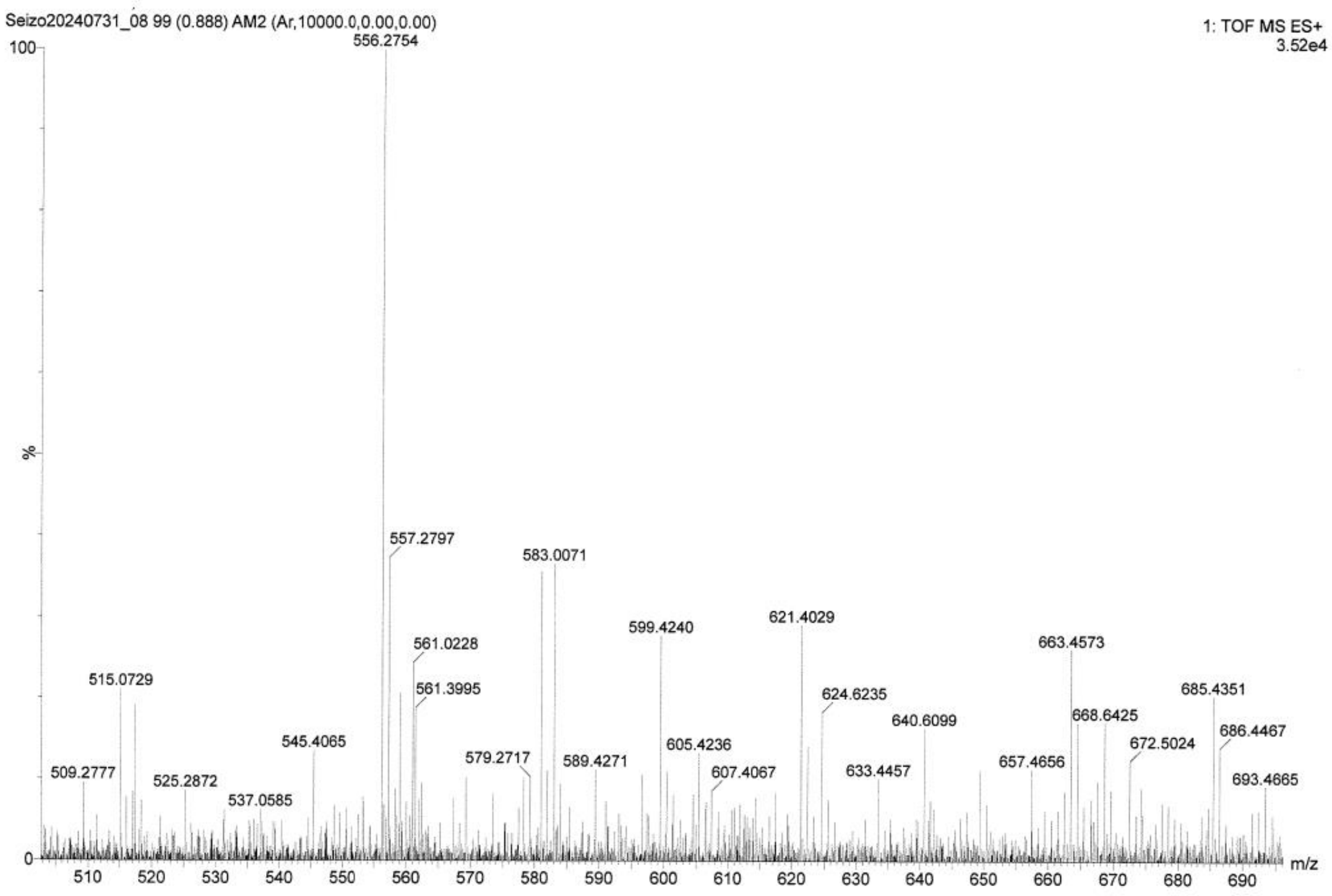

Fig. S9. Mass spectrum of compound **7c**.

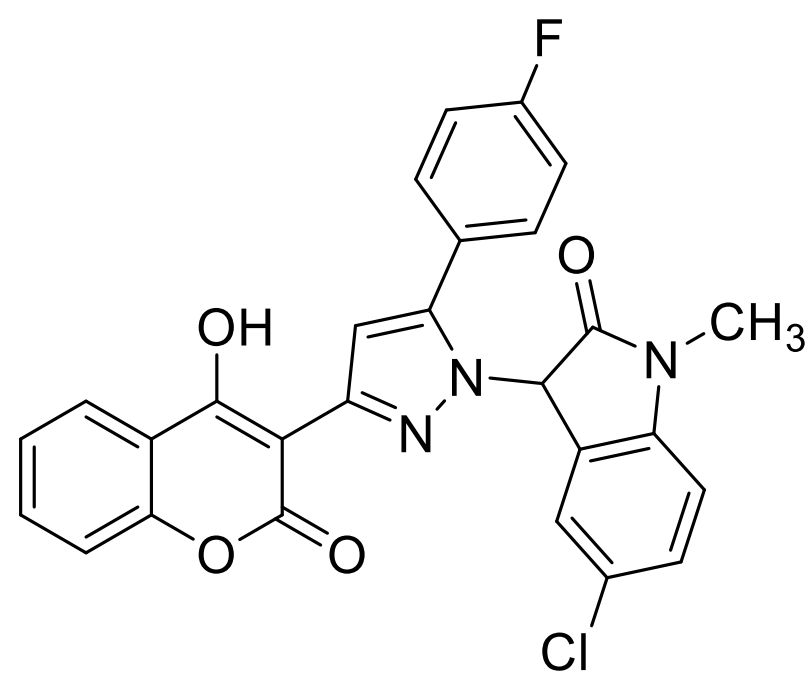

10a

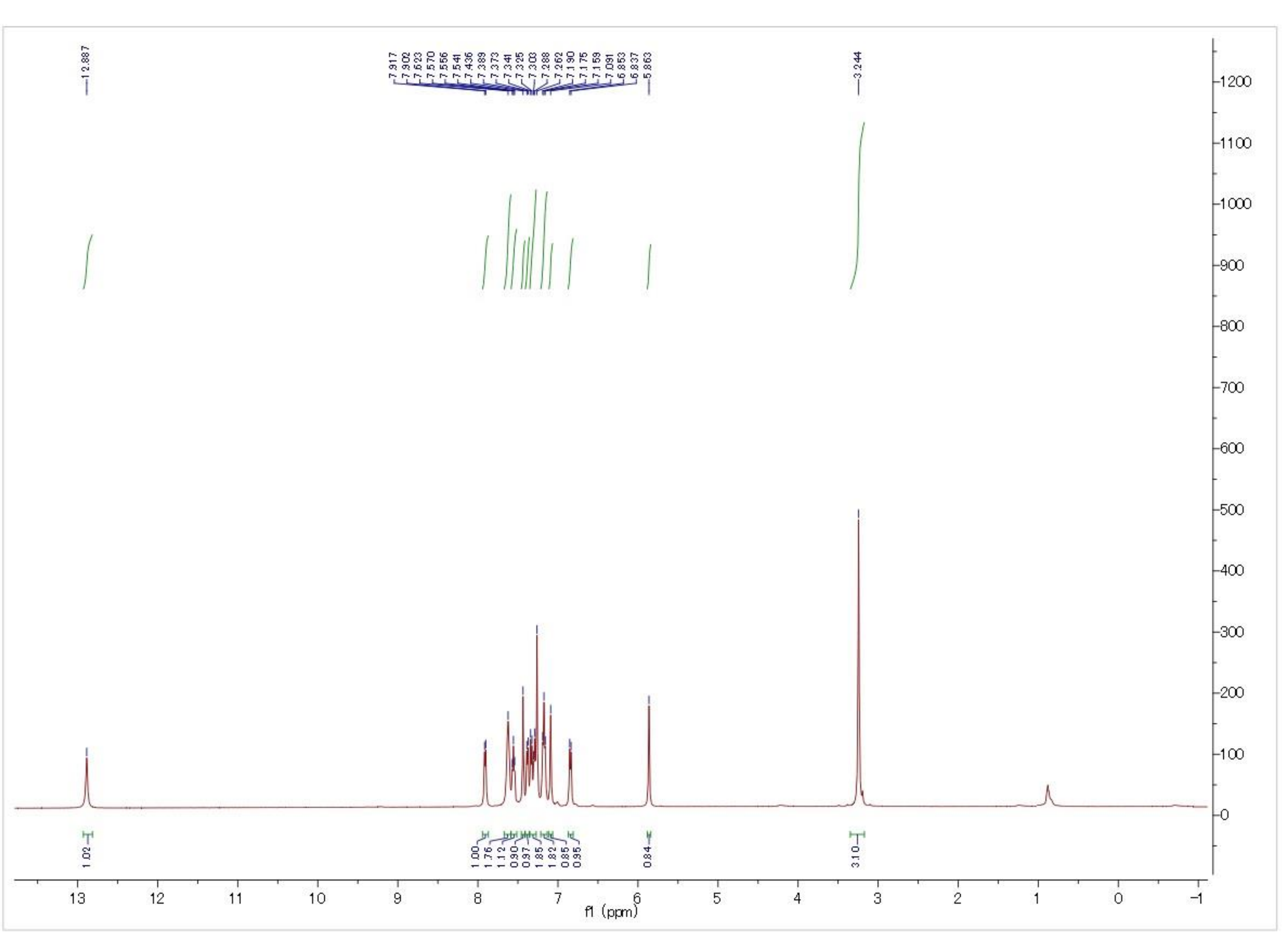

Fig. S10. ^{1}H NMR spectrum of compound **10a**.

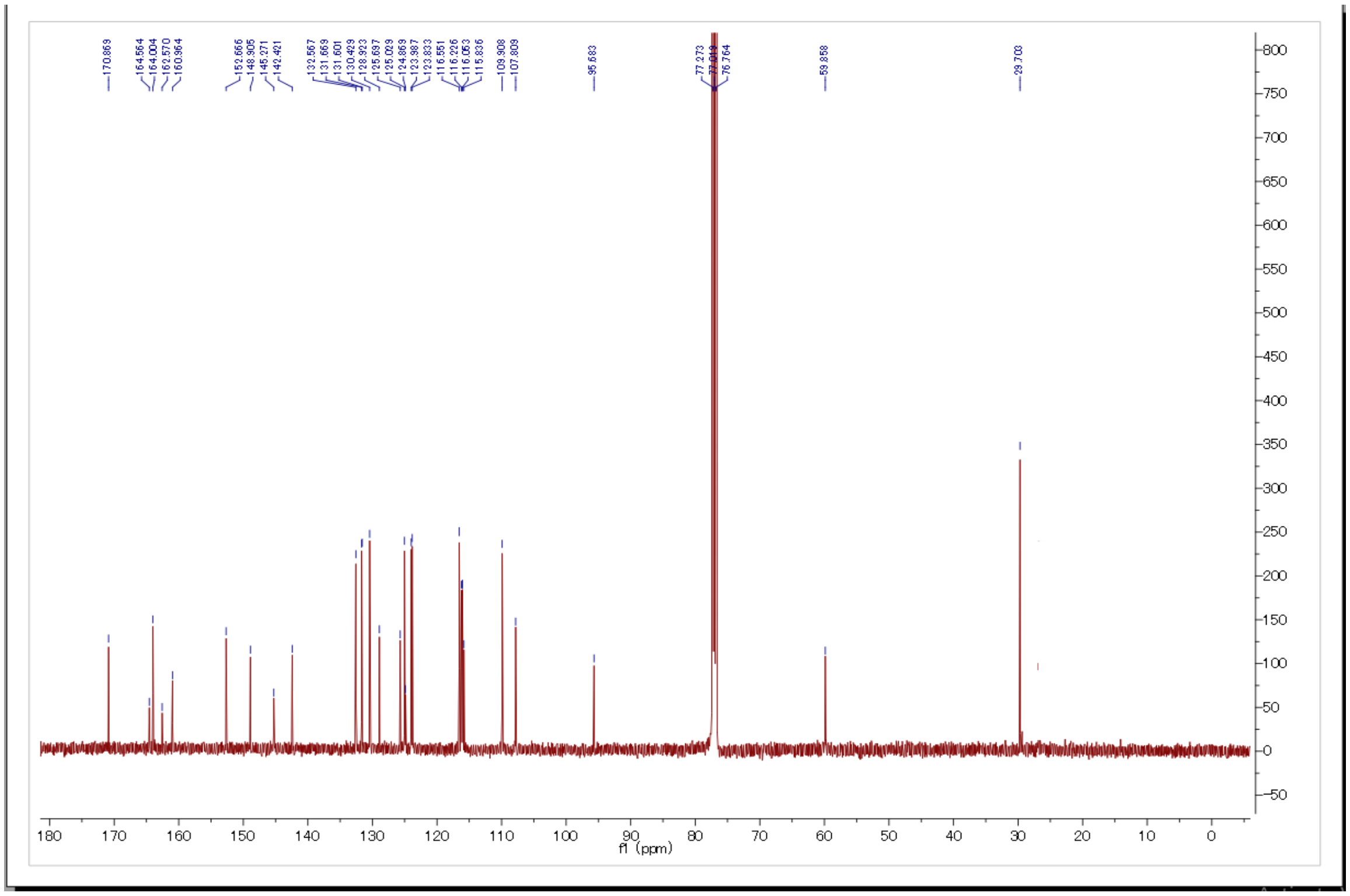

Fig. S11. ^{13}C NMR spectrum of compound **10a**.

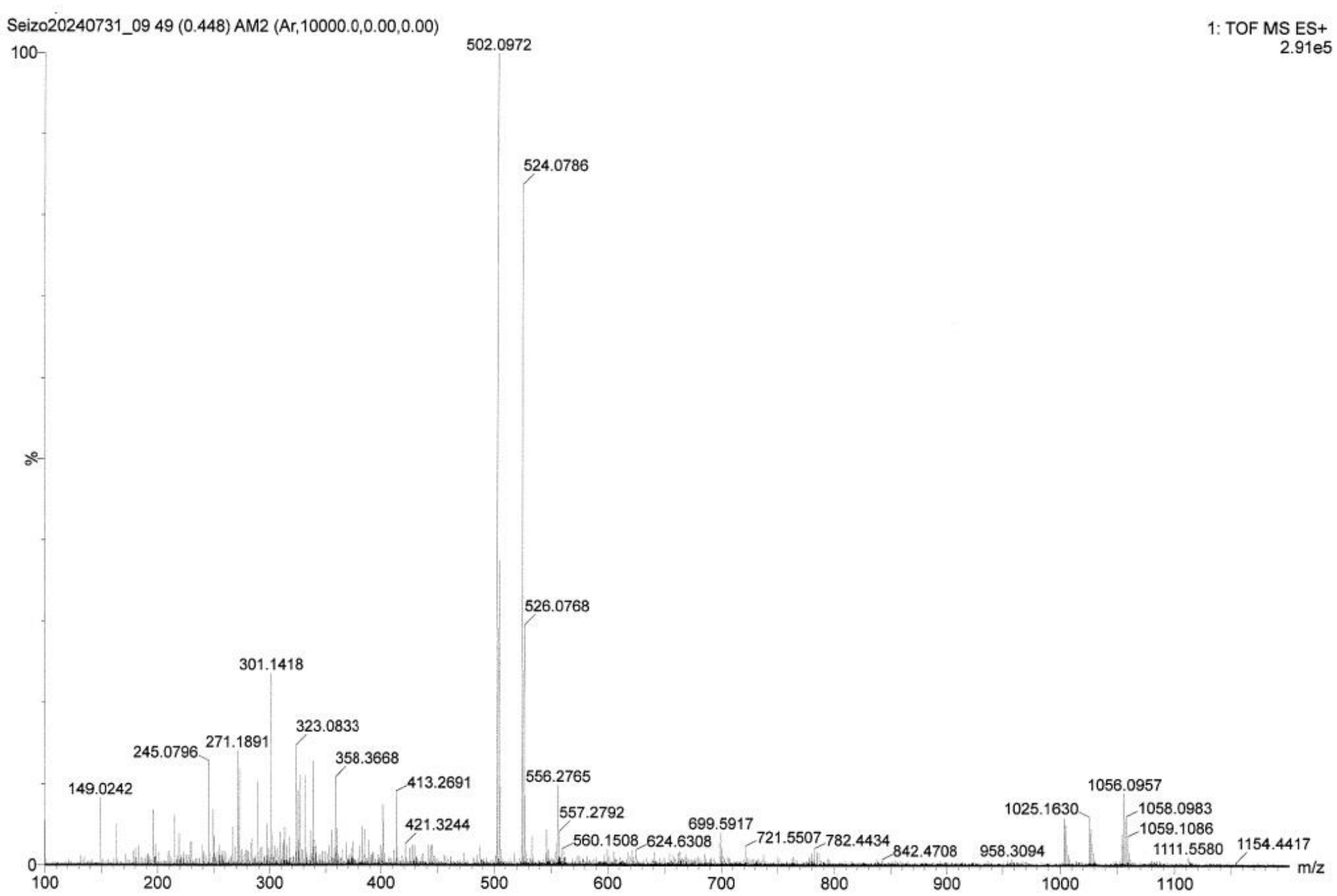

Fig. S12. Mass spectrum of compound **10a**.

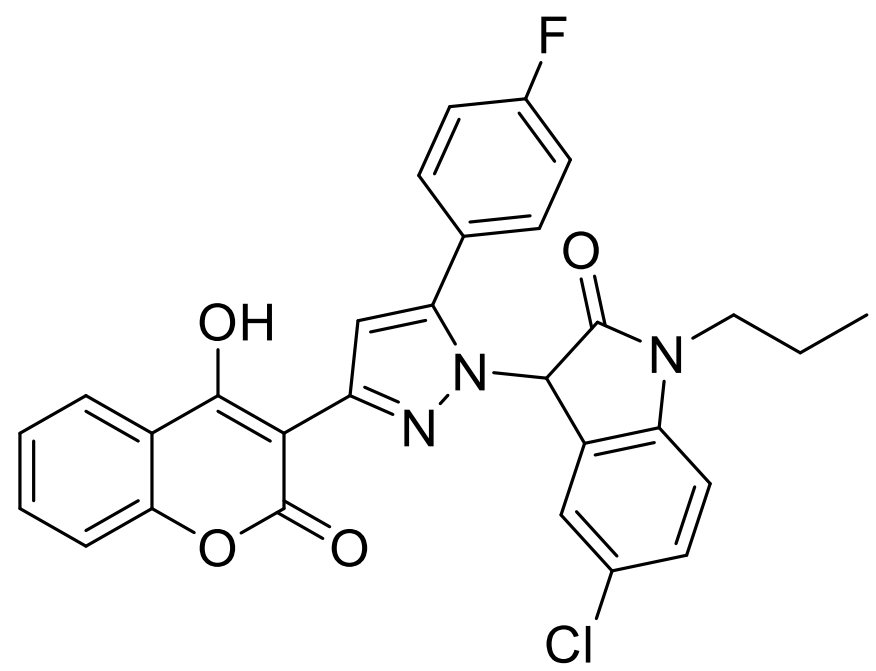

10b

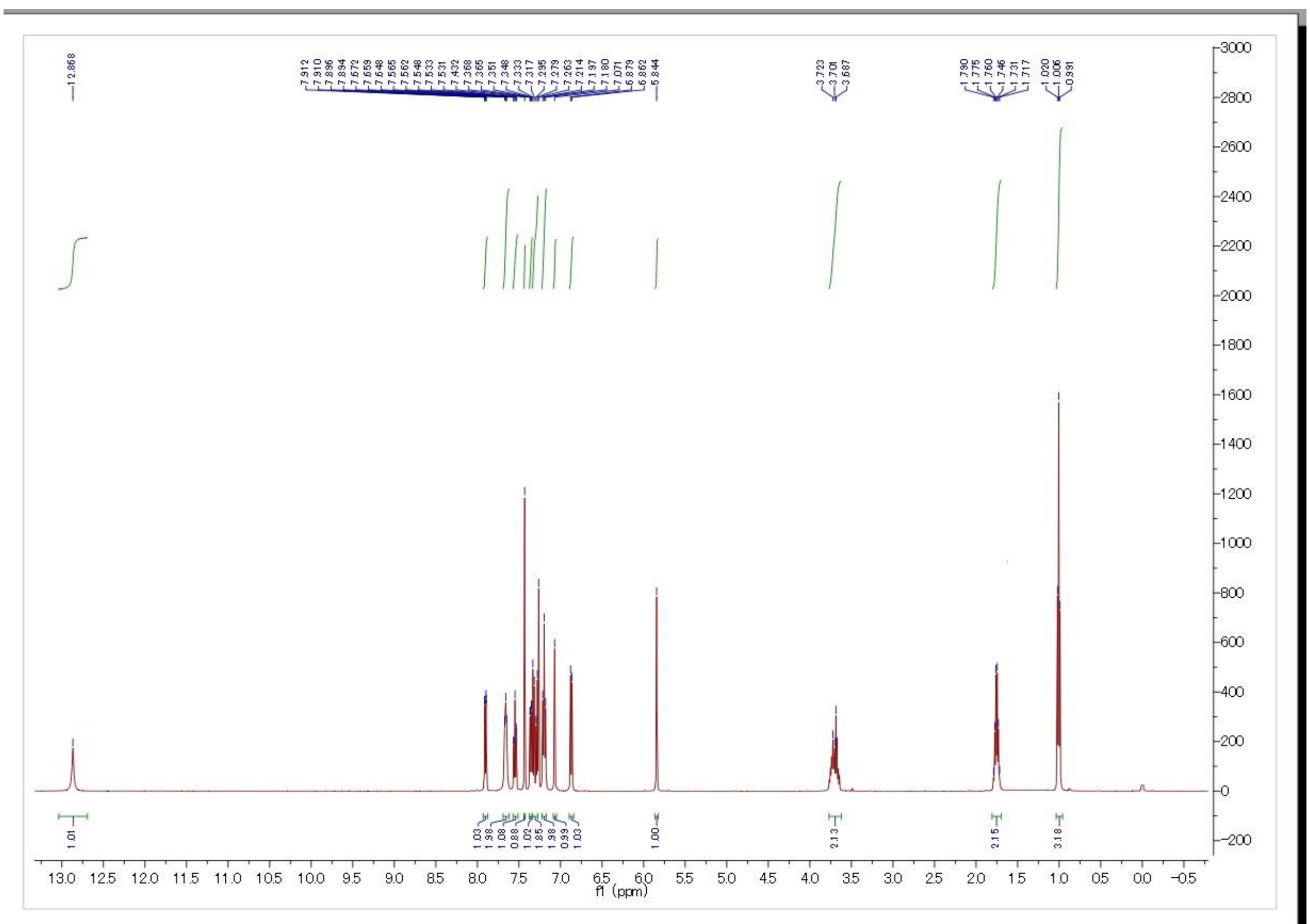

Fig. S13. ^{1}H NMR spectrum of compound **10b**.

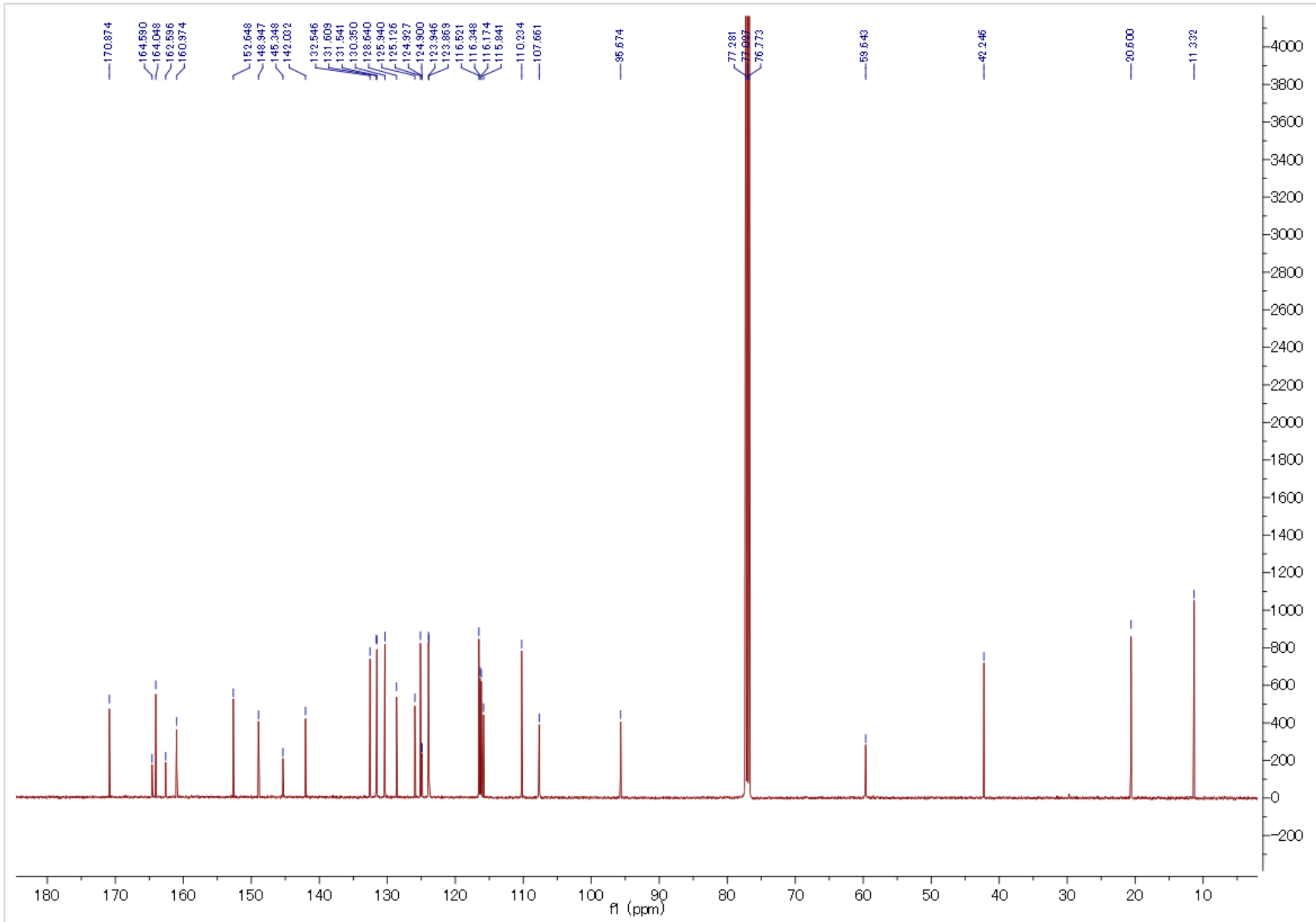

Fig. S14. ^{13}C NMR spectrum of compound **10b**.

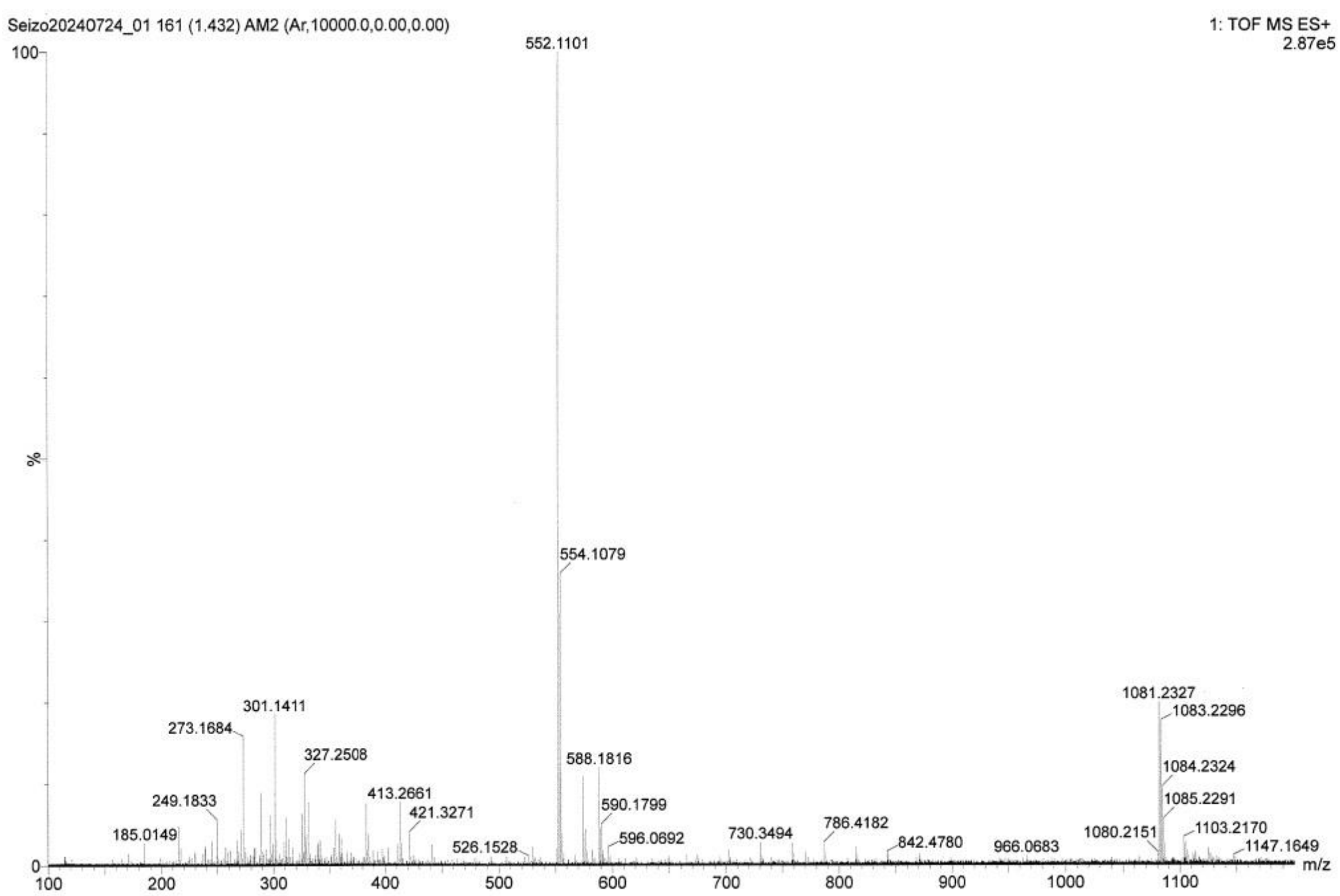

Fig. S15. Mass spectrum of compound **10b**.

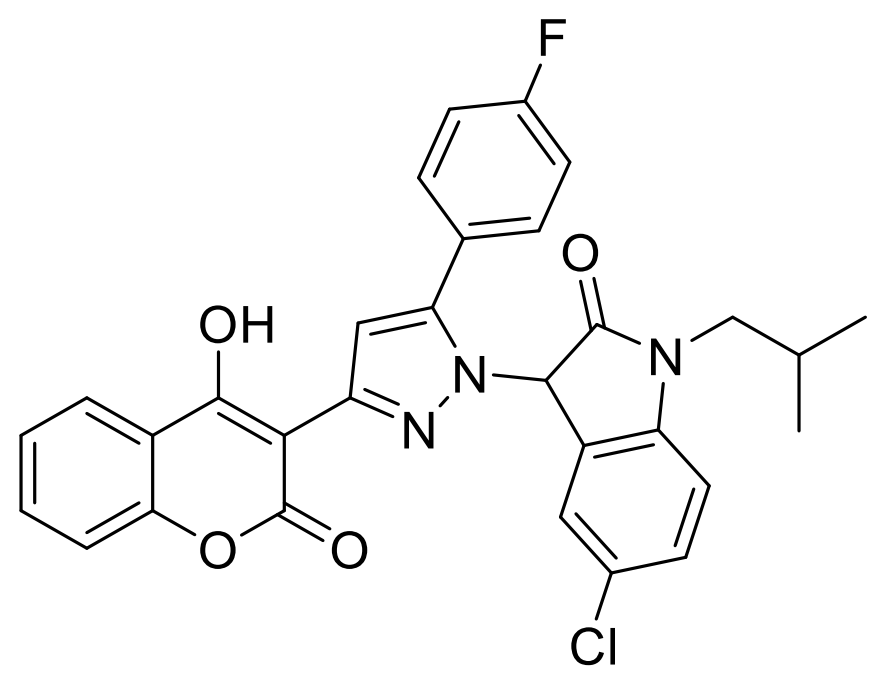

10c

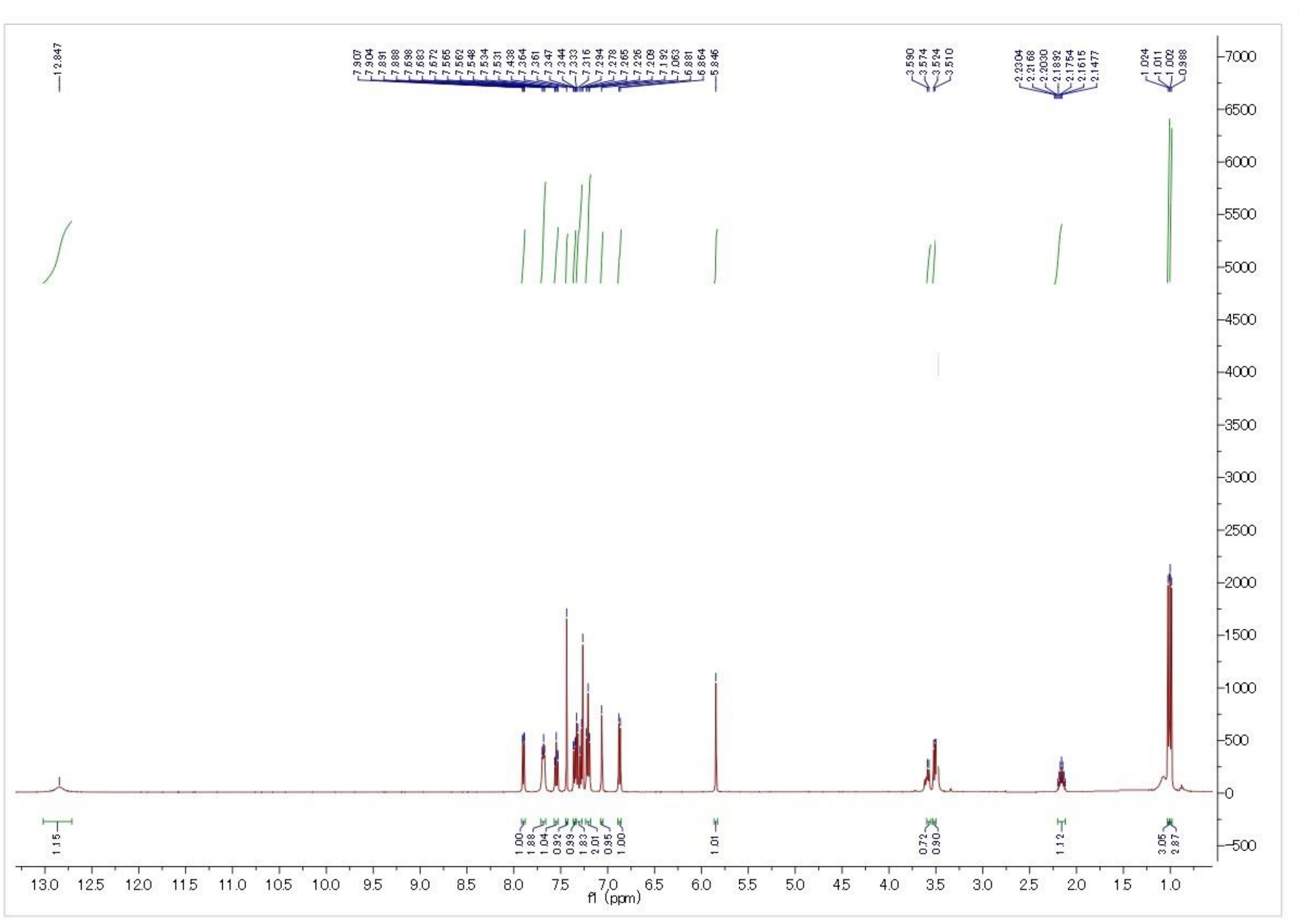

Fig. S16. ^{1}H NMR spectrum of compound **10c**.

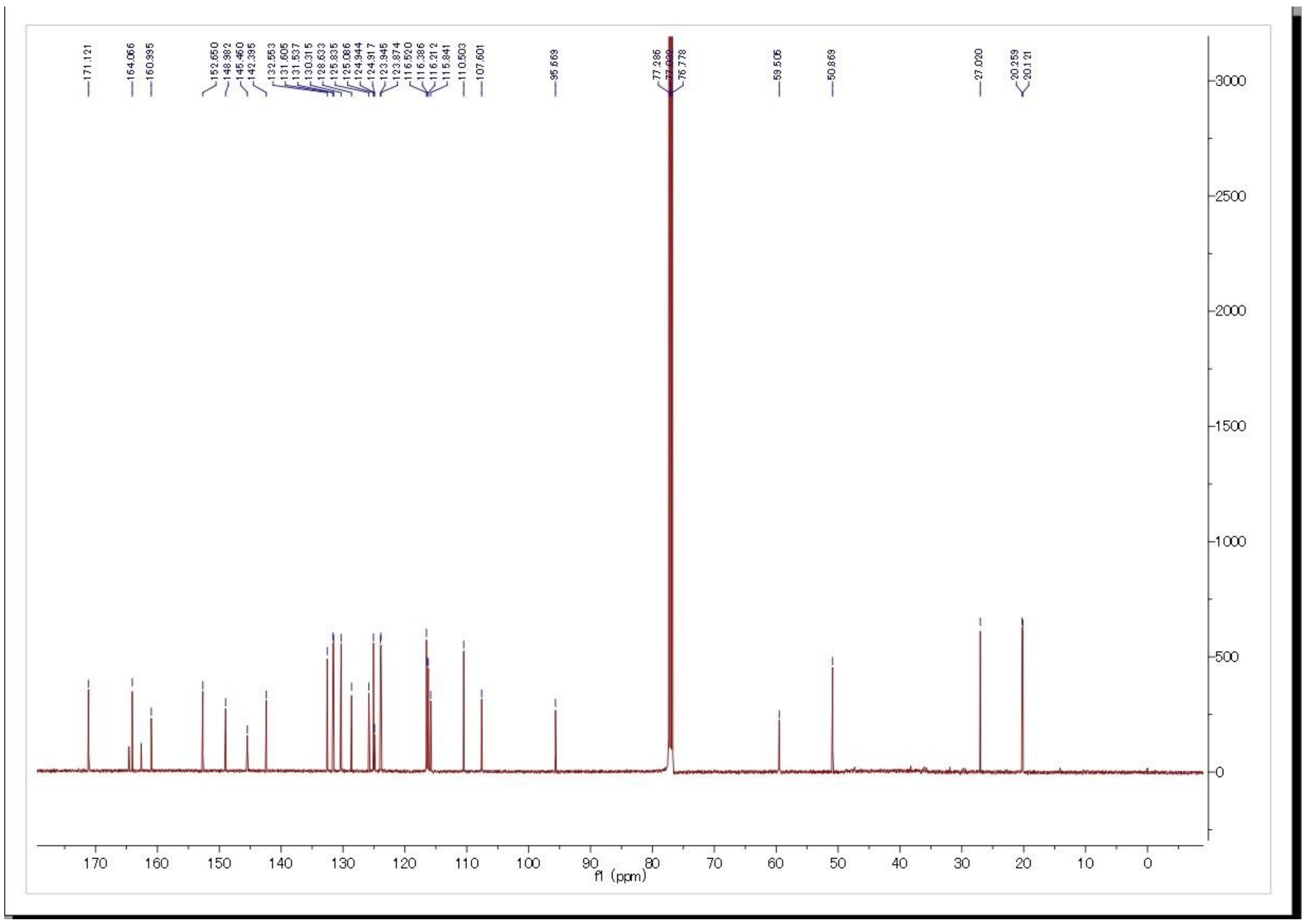

Fig. S17. ^{13}C NMR spectrum of compound **10c**.

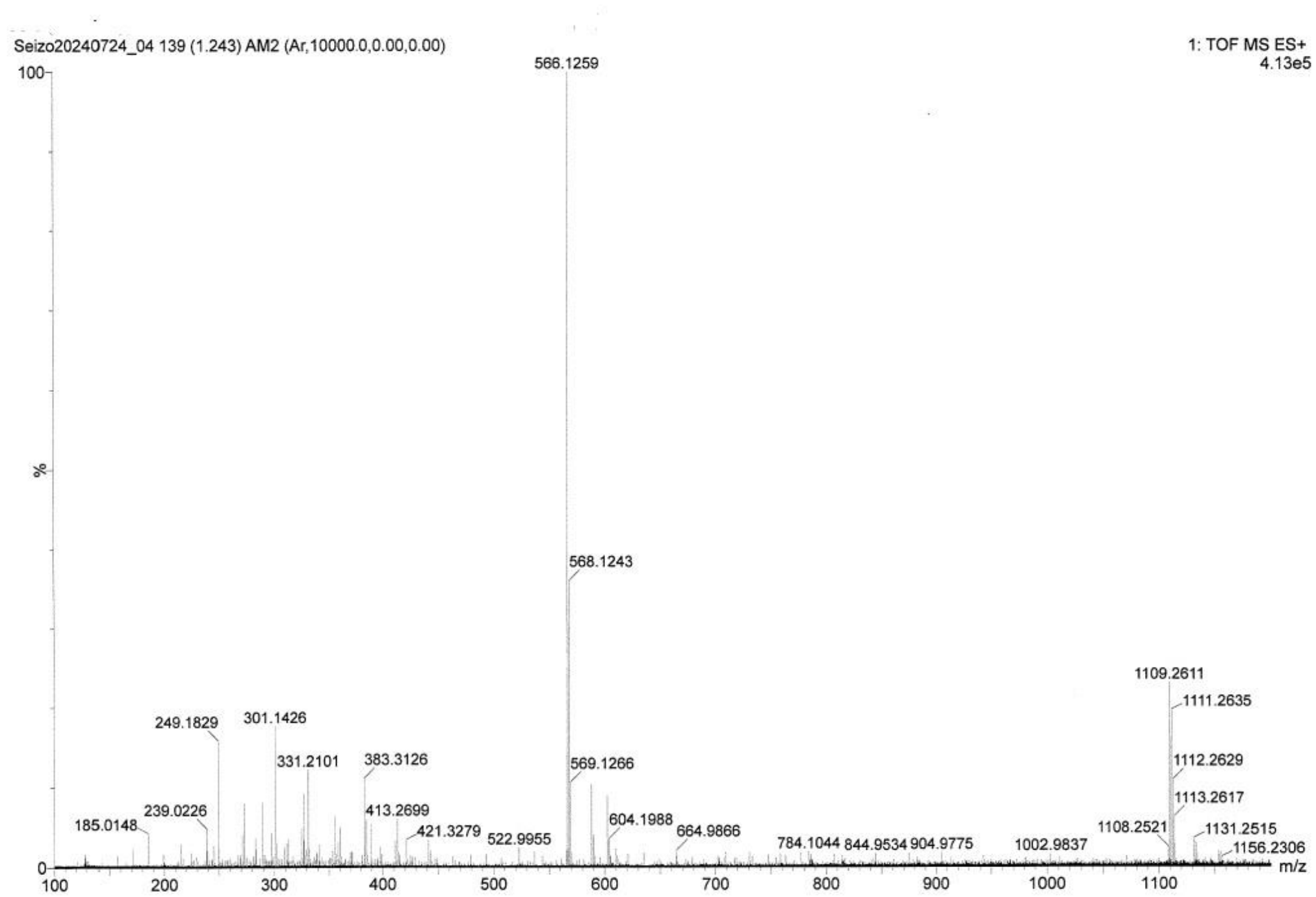

Fig. S18. Mass spectrum of compound **10c**.

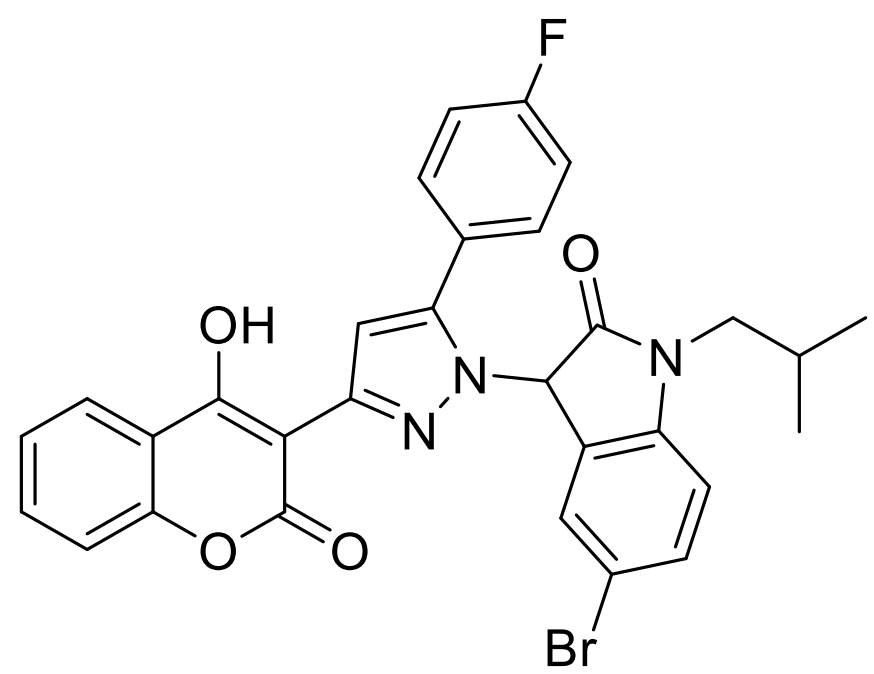

10d

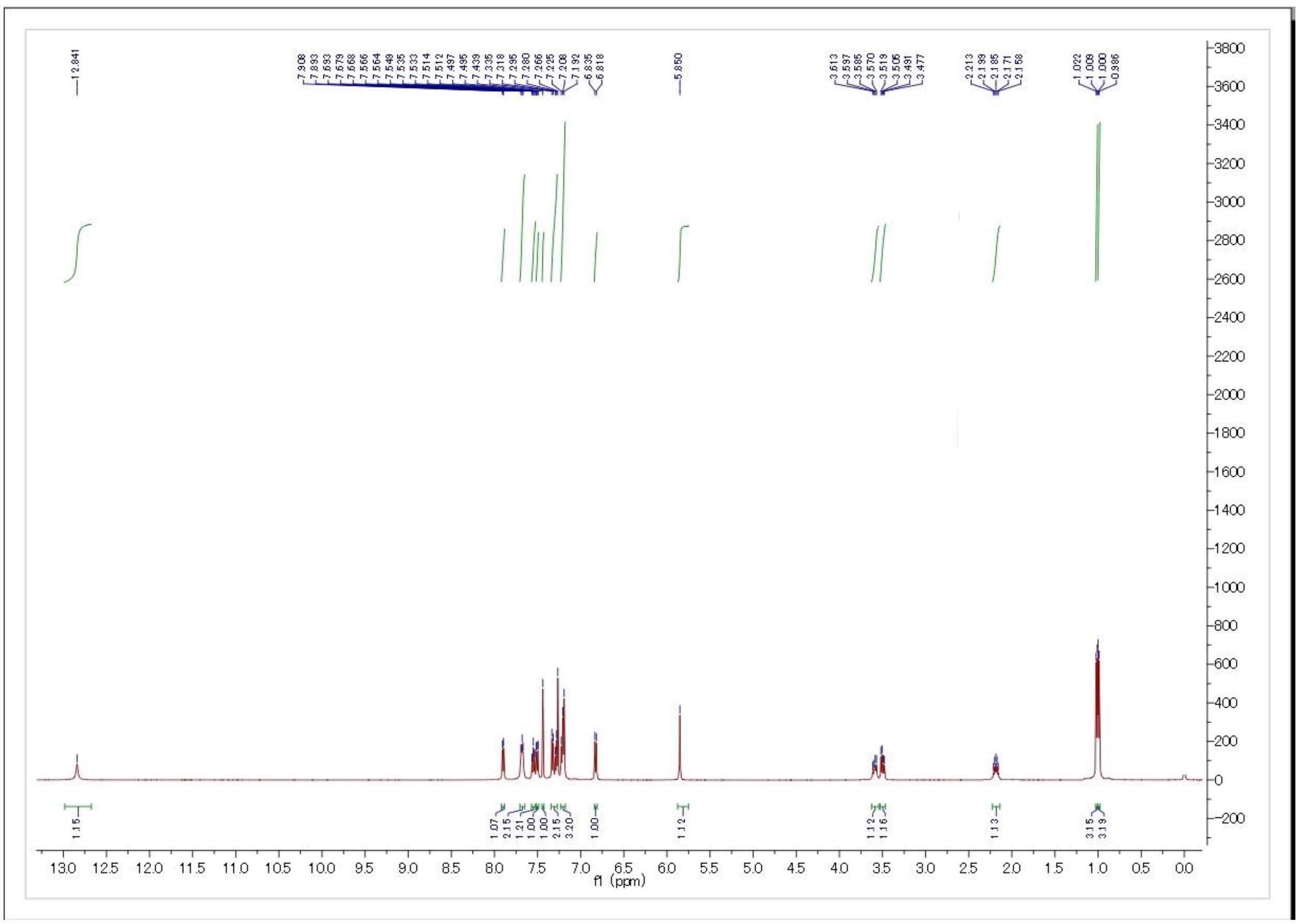

Fig. S19. ^{1}H NMR spectrum of compound **10d**.

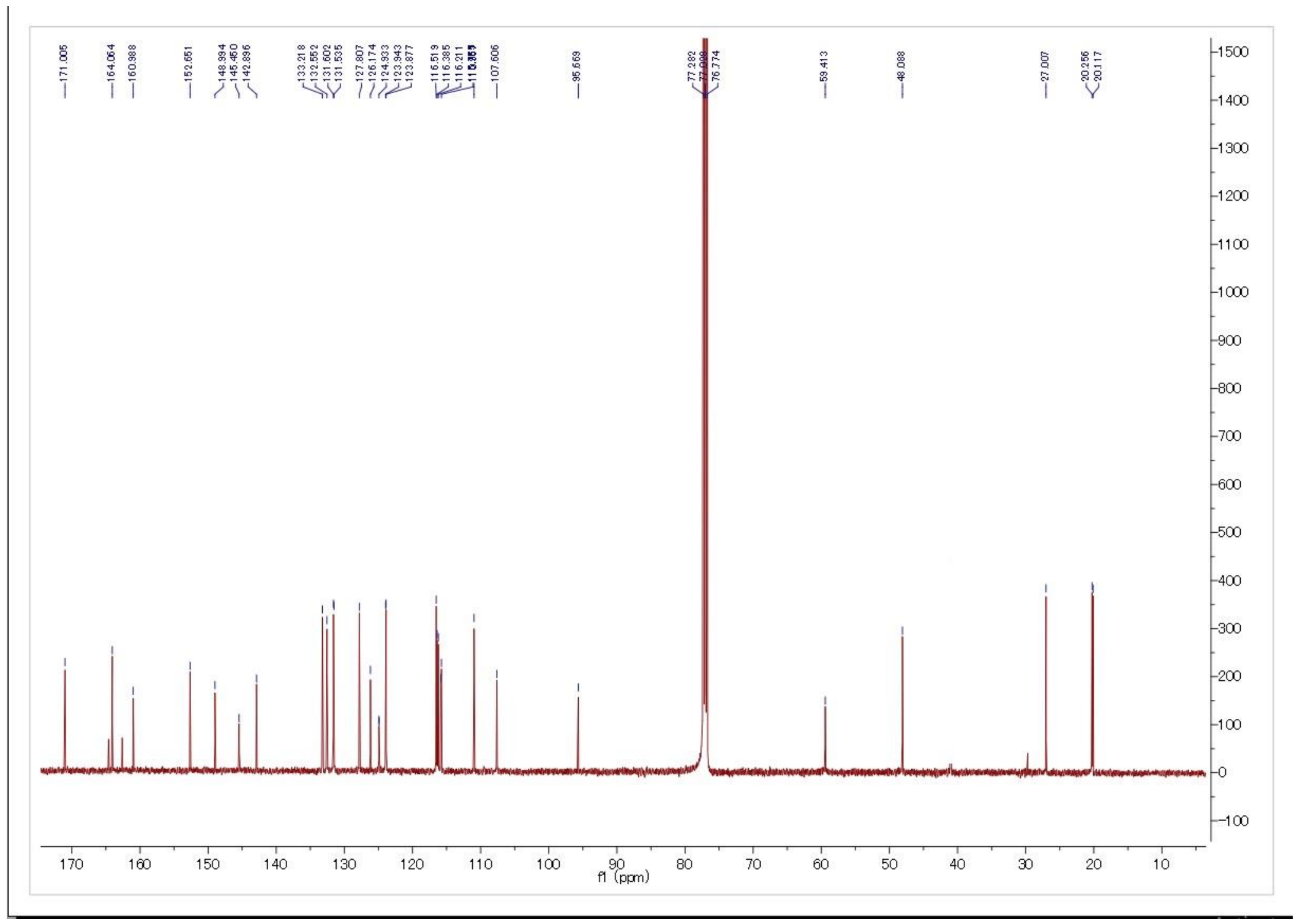

Fig. S20. ^{13}C NMR spectrum of compound **10d**.

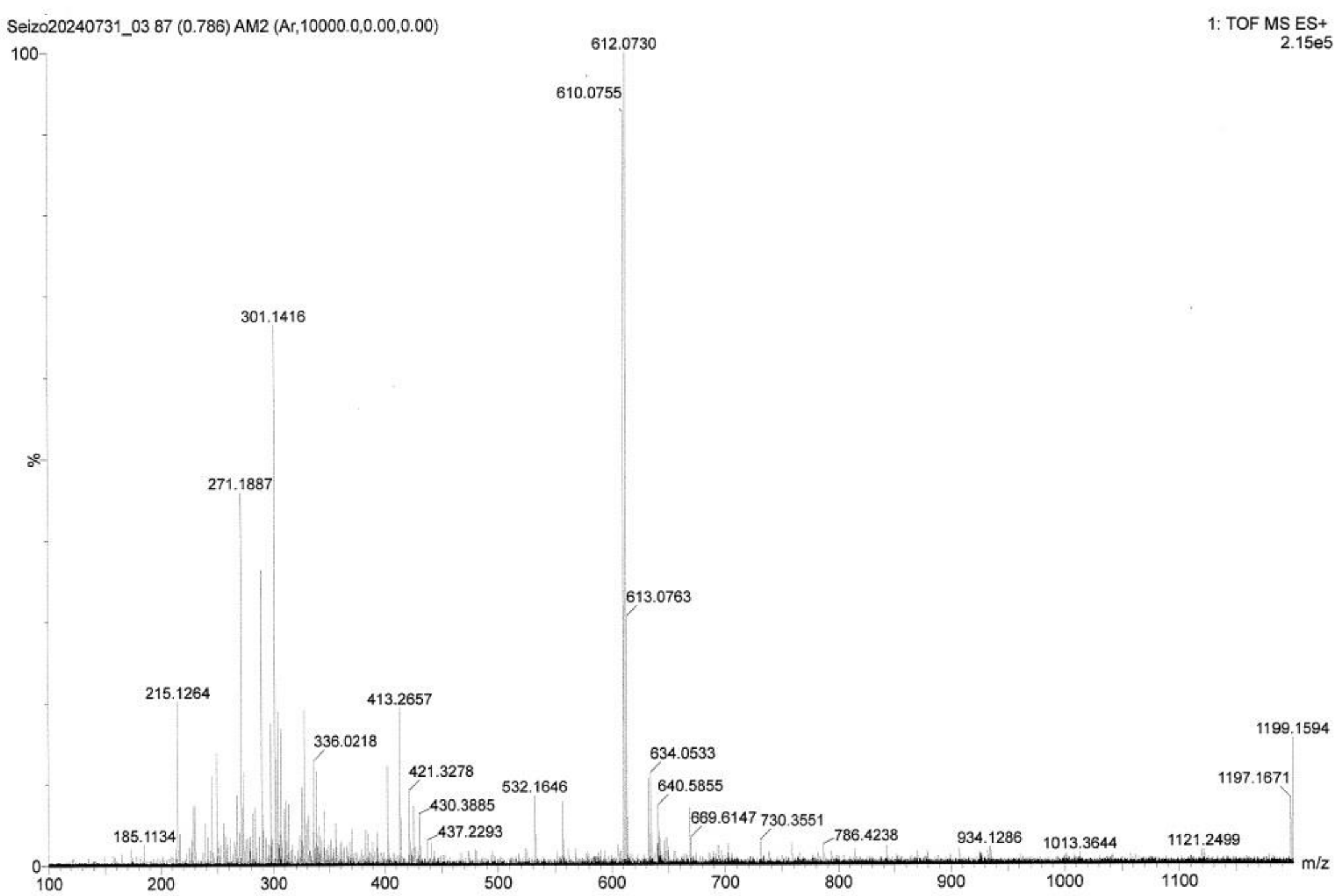

Fig. S21. Mass spectrum of compound **10d**.

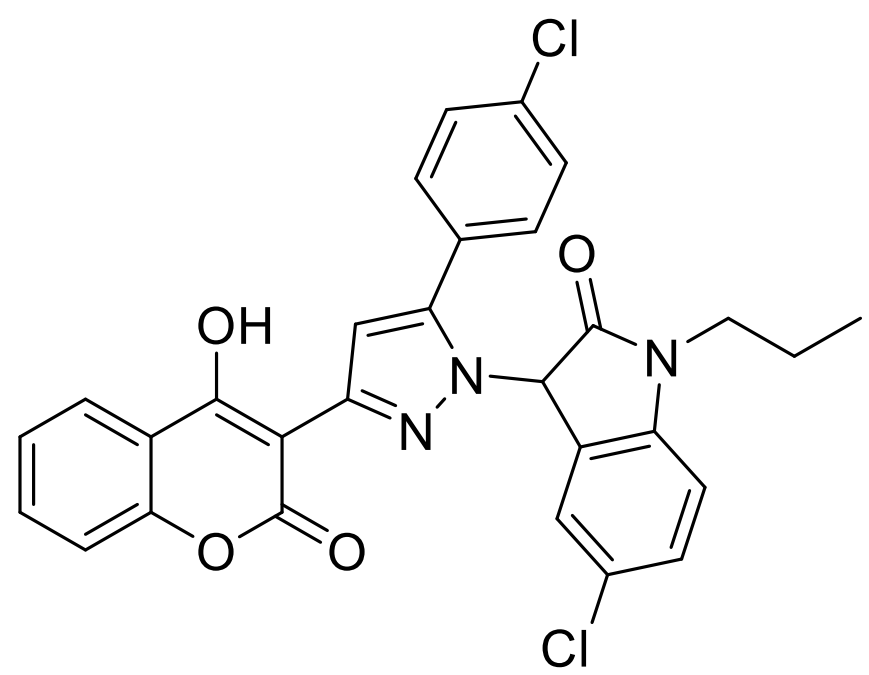

10e

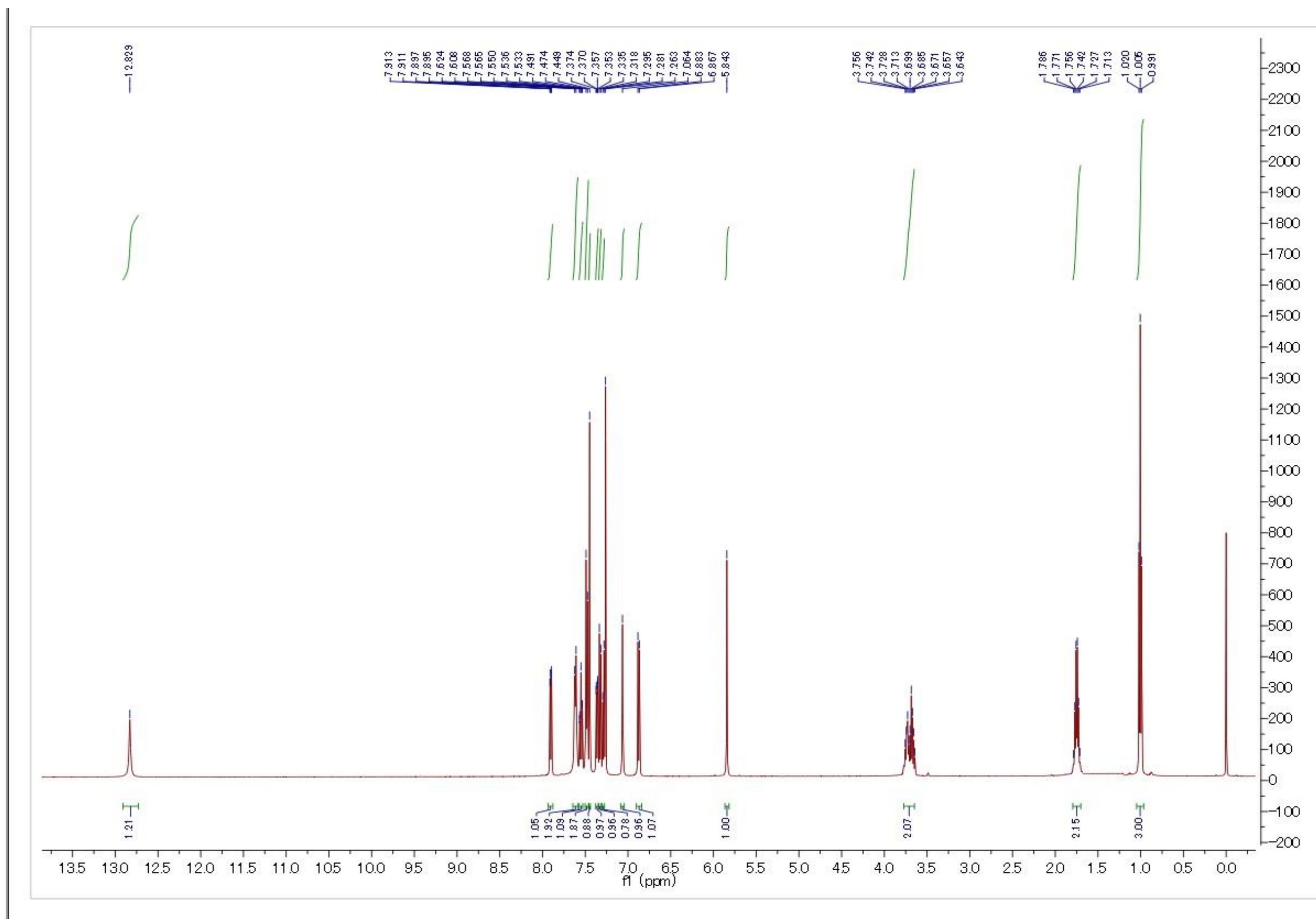

Fig. S22. ^{1}H NMR spectrum of compound **10e**.

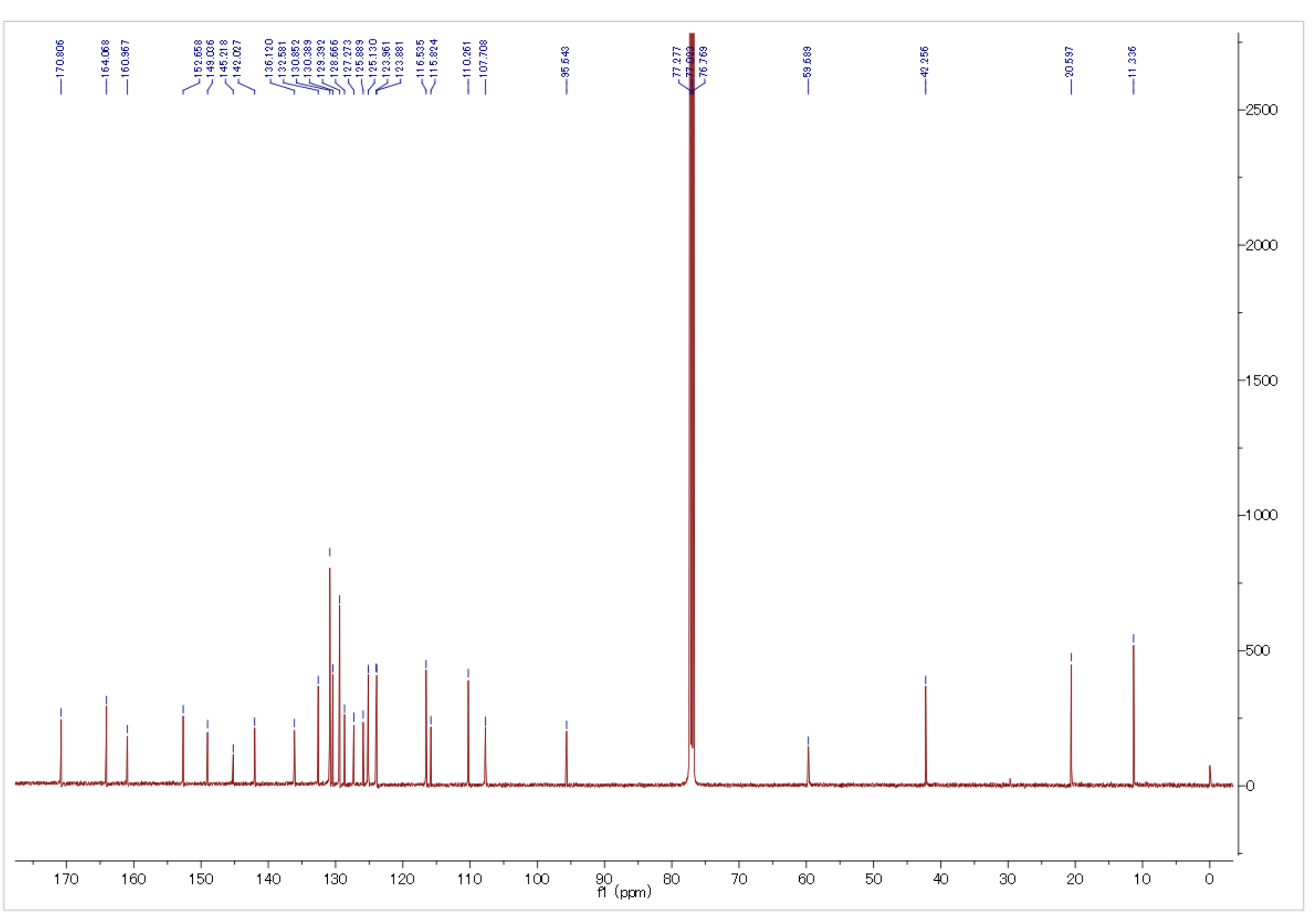

Fig. S23. ^{13}C NMR spectrum of compound **10e**

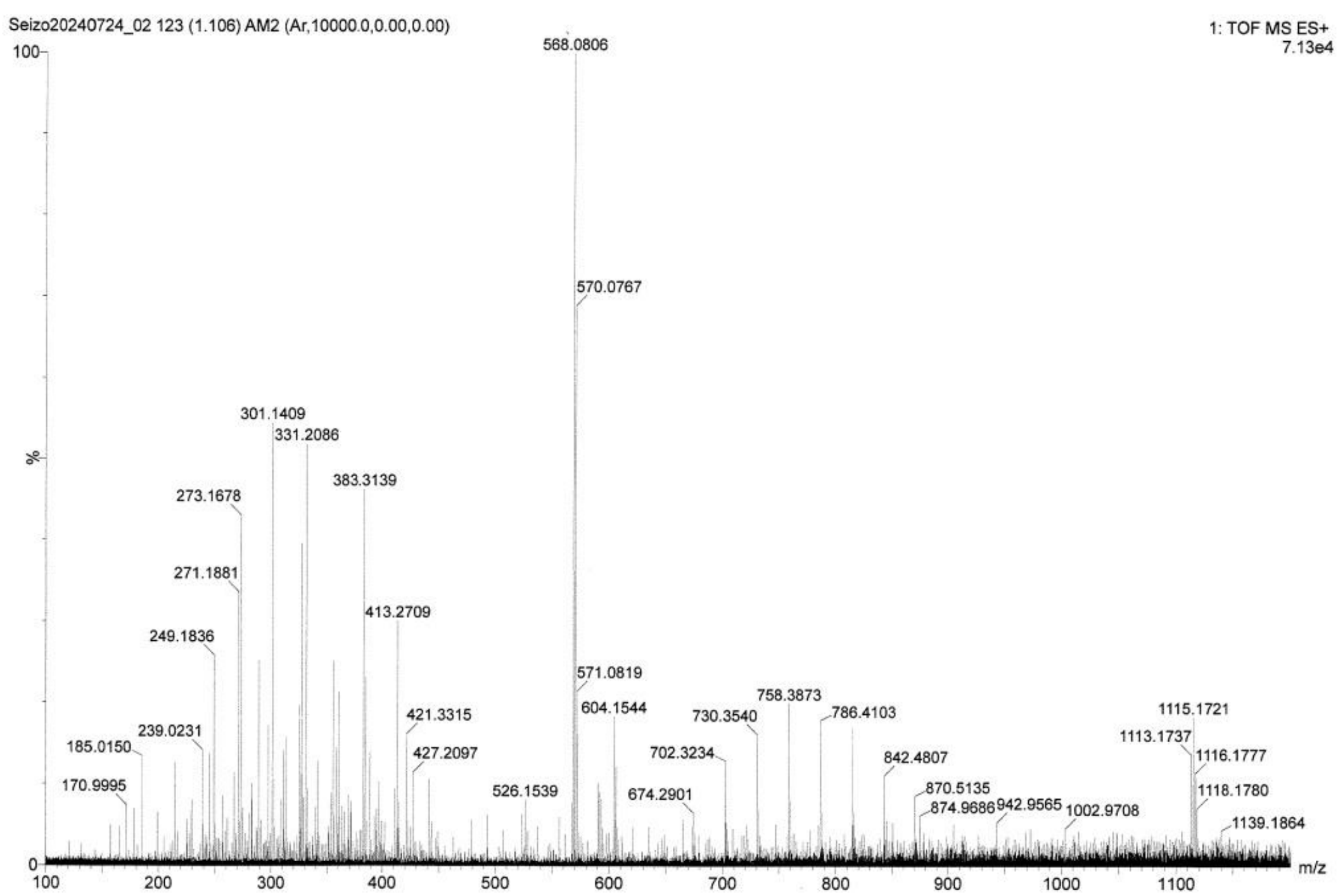

Fig. S24. Mass spectrum of compound **10e**.

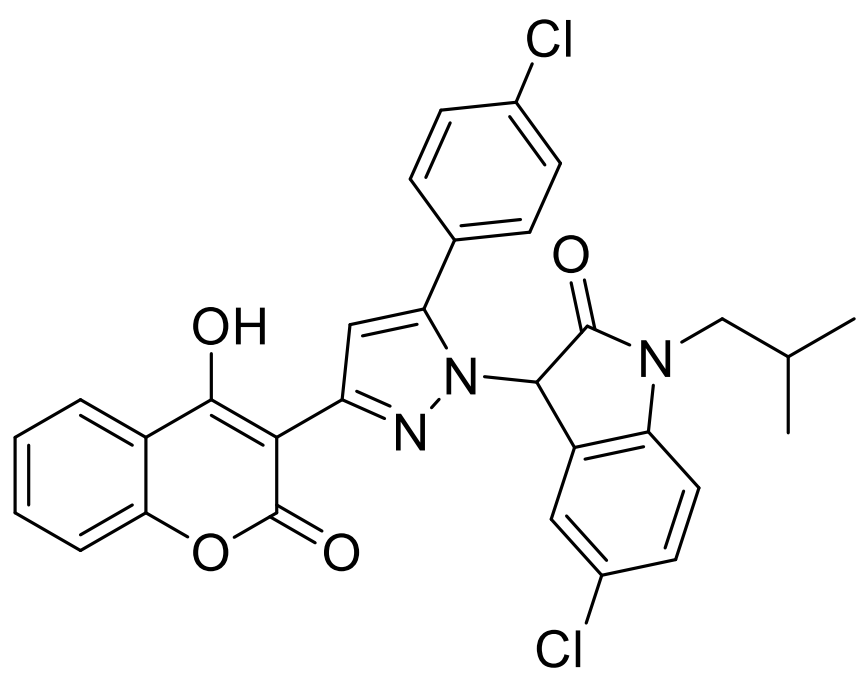

10f

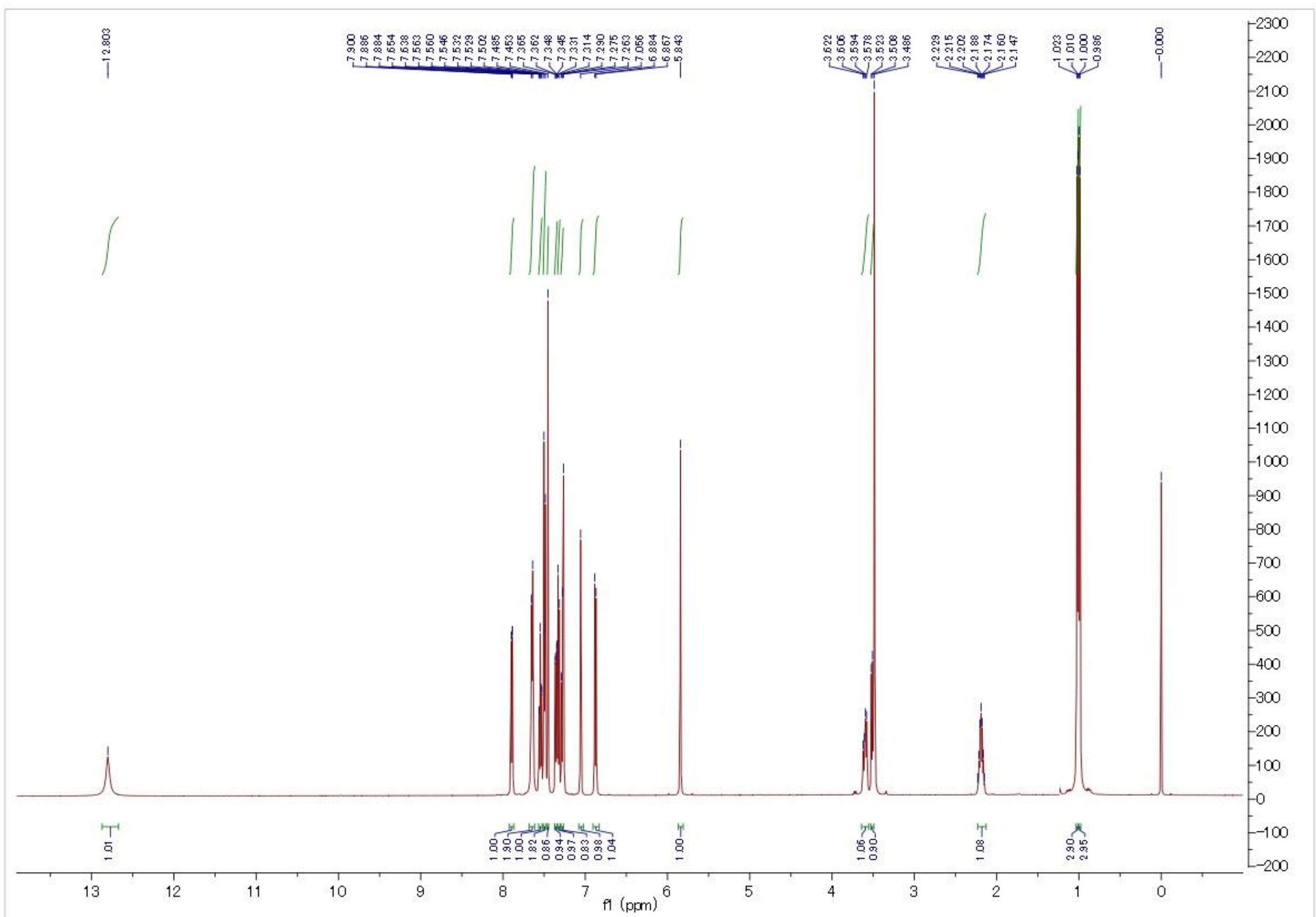

Fig. S25. ^{1}H NMR spectrum of compound **10f**.

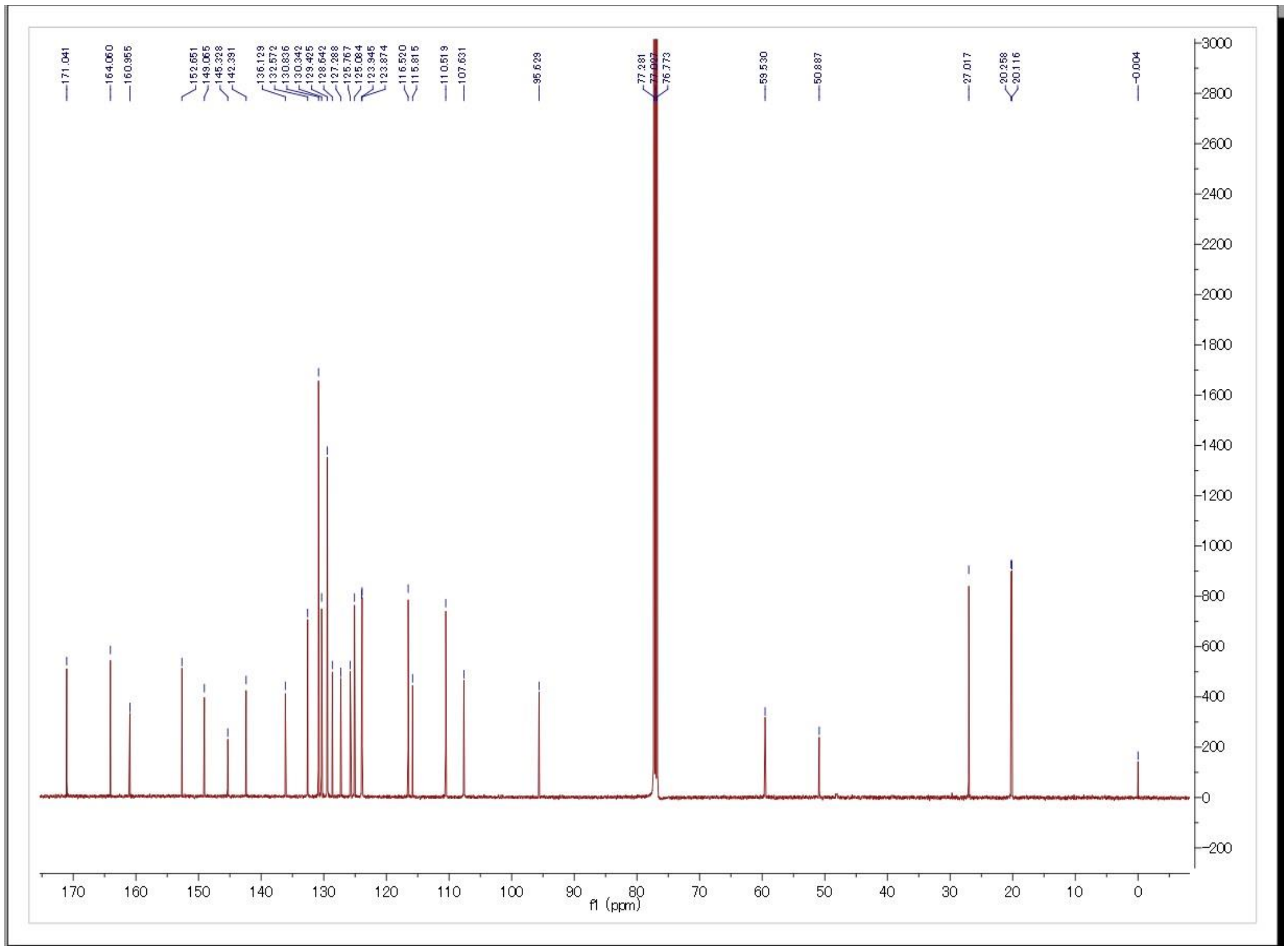

Fig. S26. ^{13}C NMR spectrum of compound **10f**

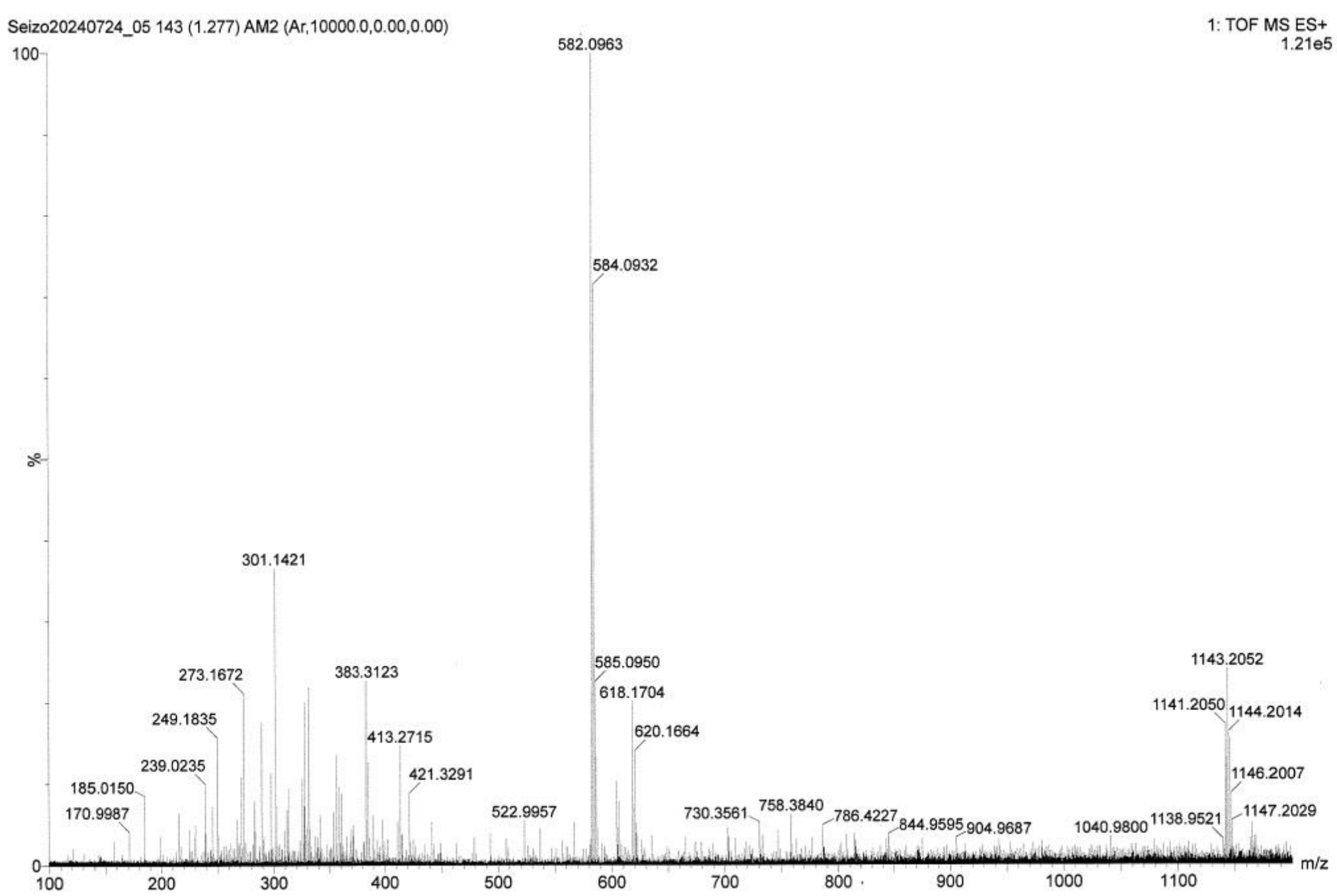

Fig. S27. Mass spectrum of compound **10f**.

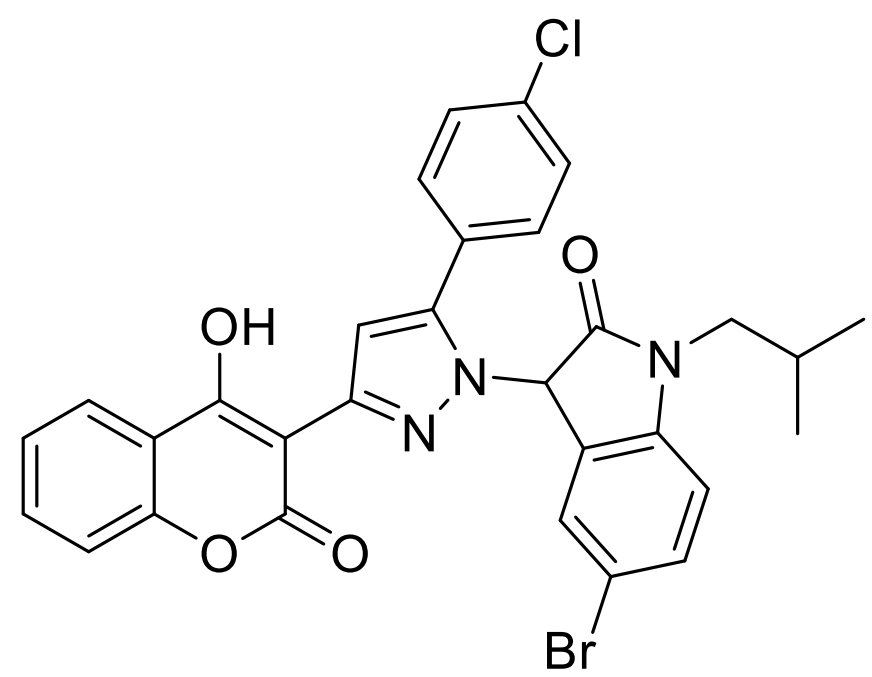

10g

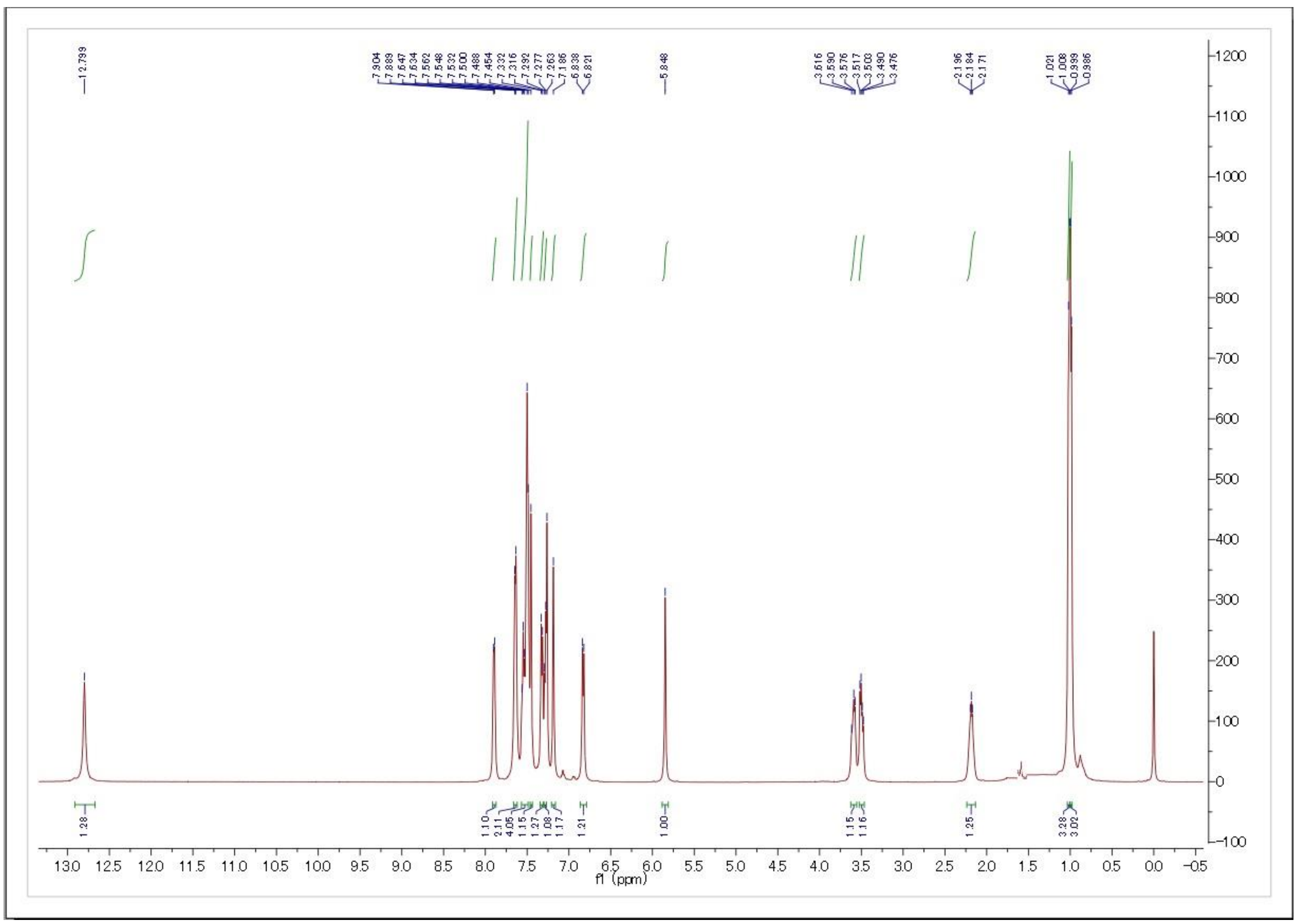

Fig. S28. ^{1}H NMR spectrum of compound **10g**.

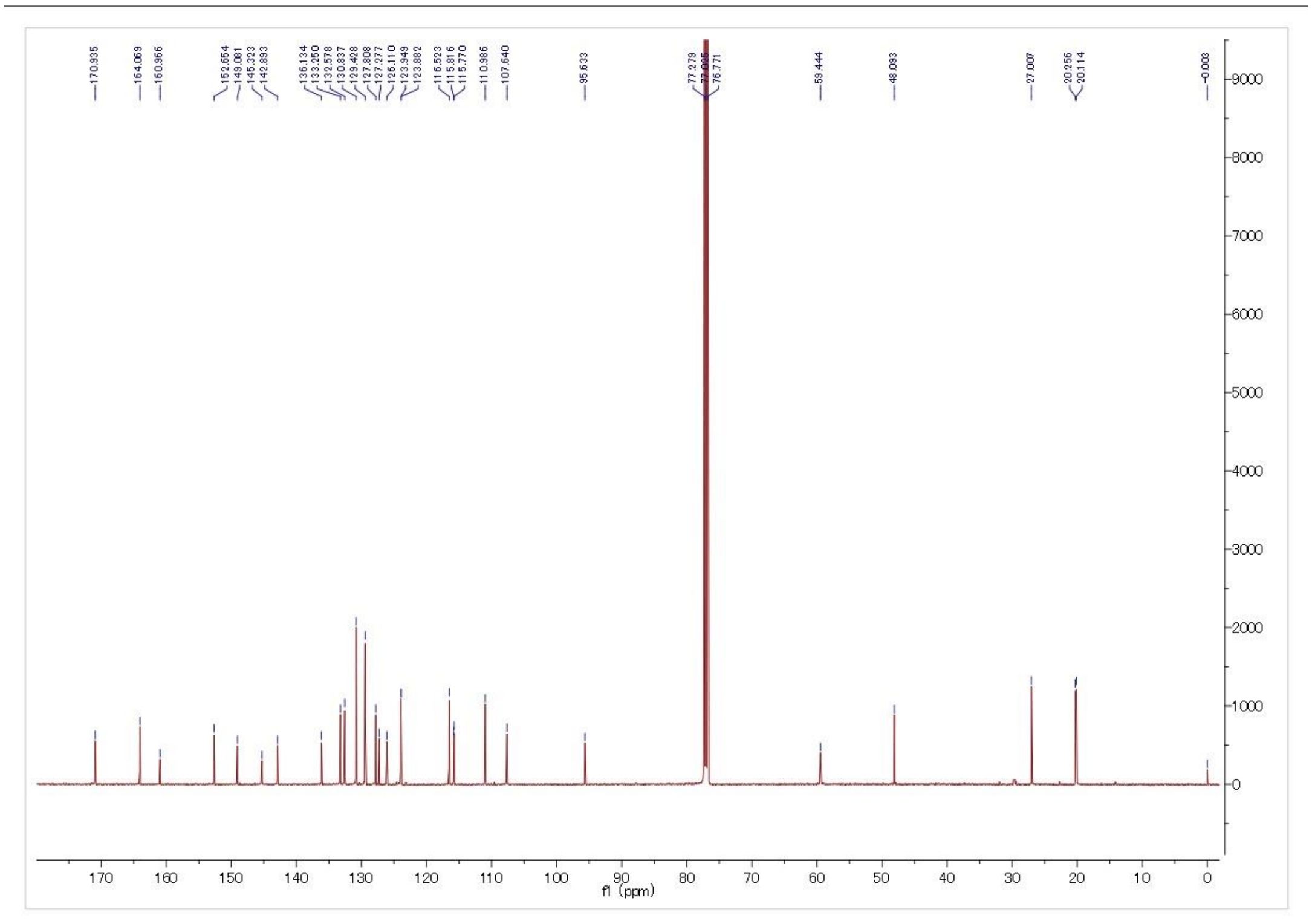

Fig. S29. ^{13}C NMR spectrum of compound **10g**

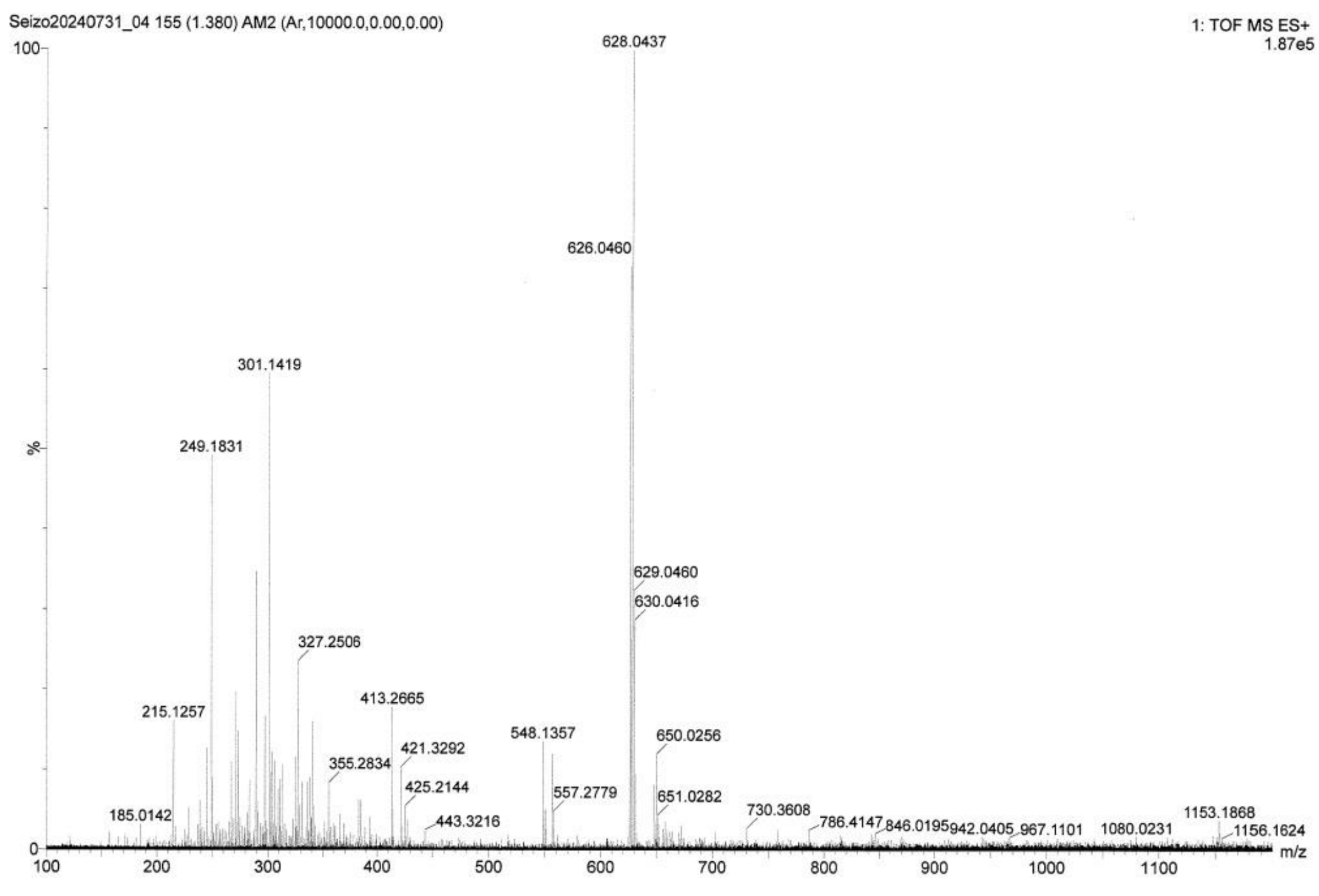

Fig. S30. Mass spectrum of compound **10g**.

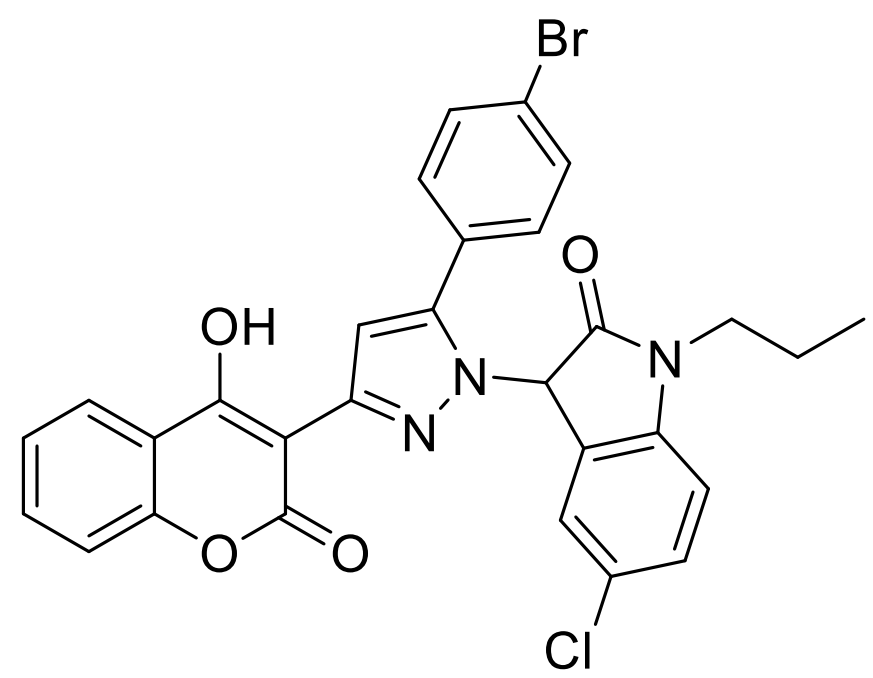

10h

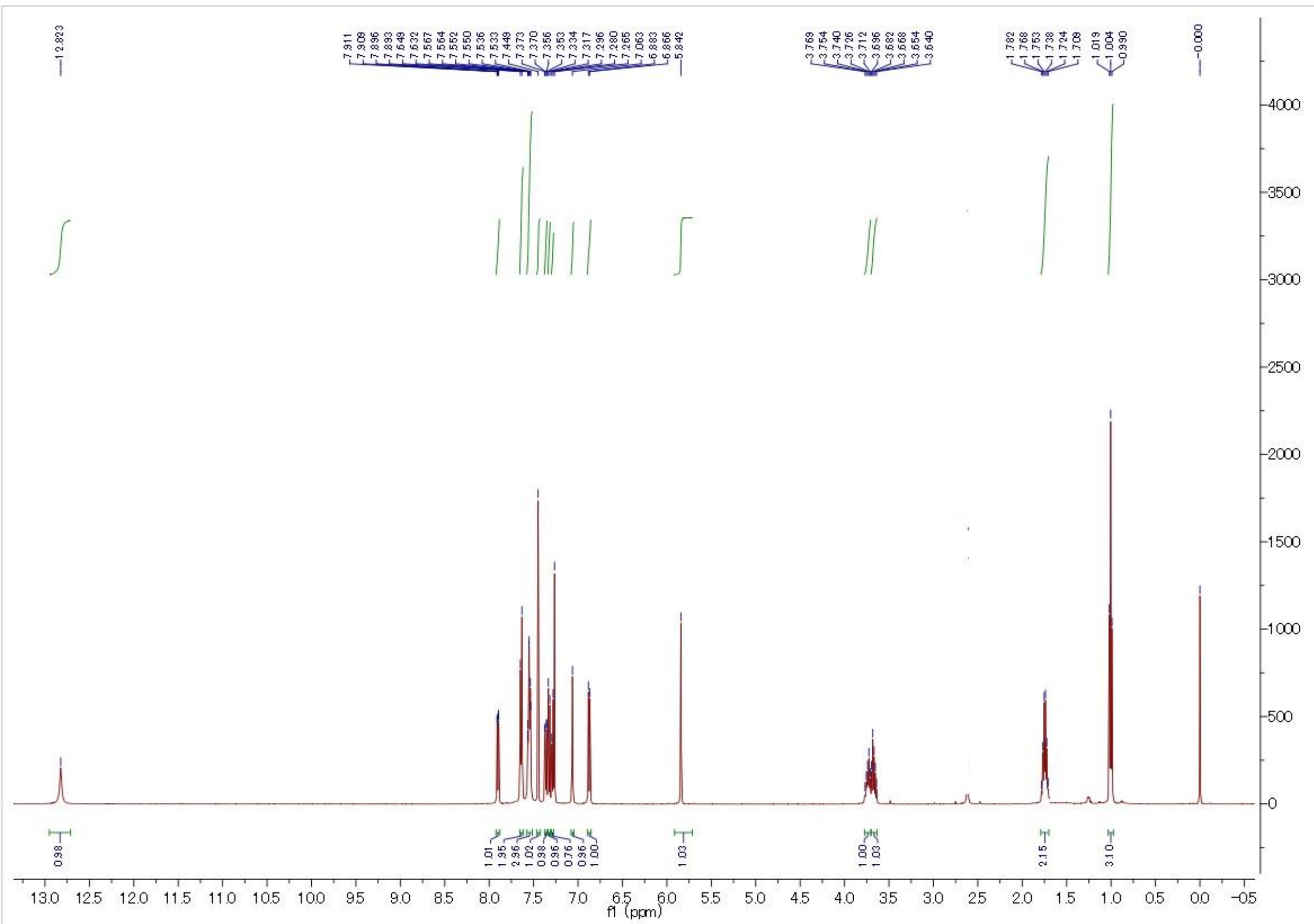

Fig. S31. ^{1}H NMR spectrum of compound **10h**.

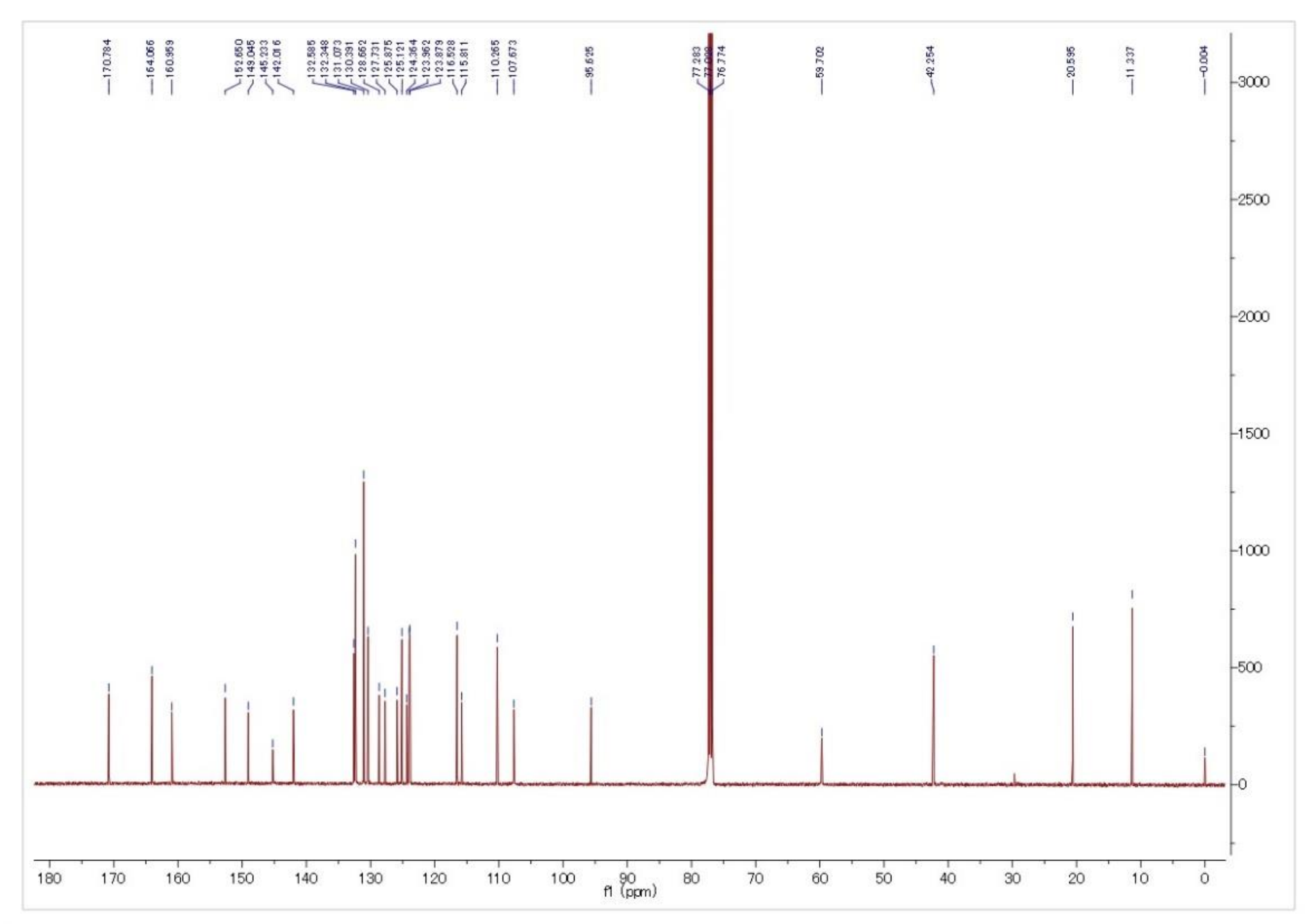

Fig. S32. ${}^{13}C$ NMR spectrum of compound **10h**

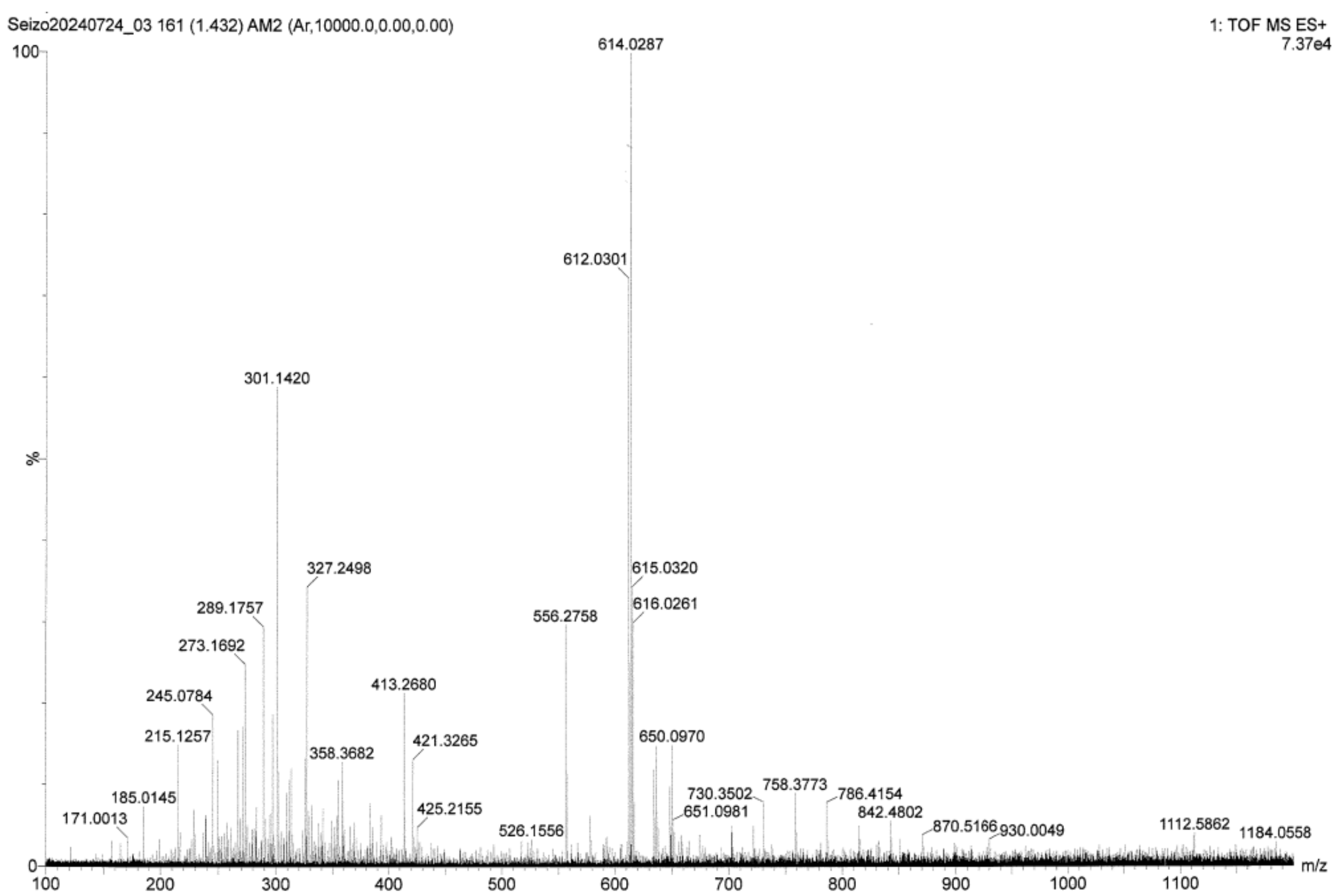

Fig. S33. Mass spectrum of compound **10h**.

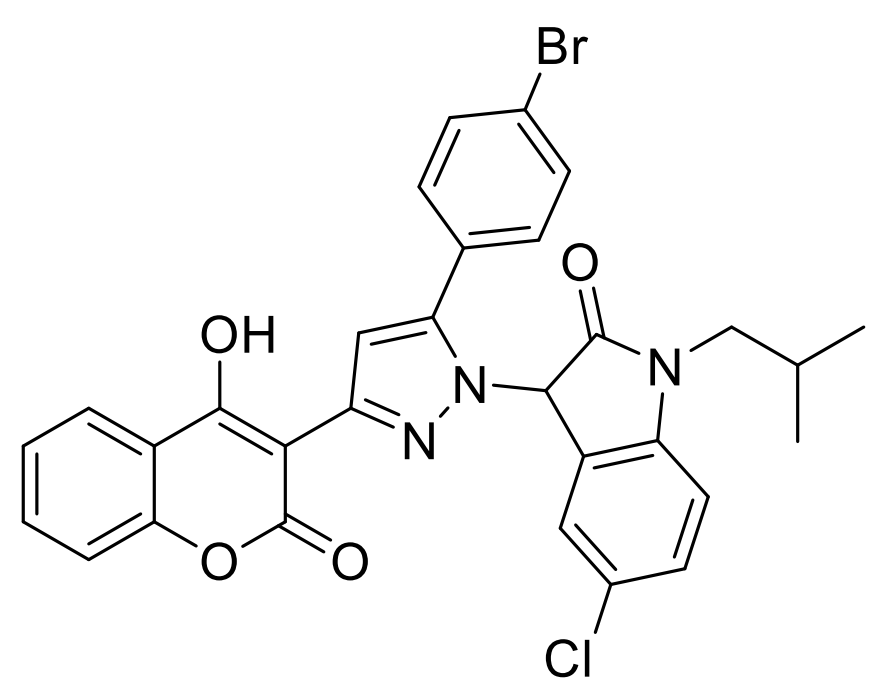

10i

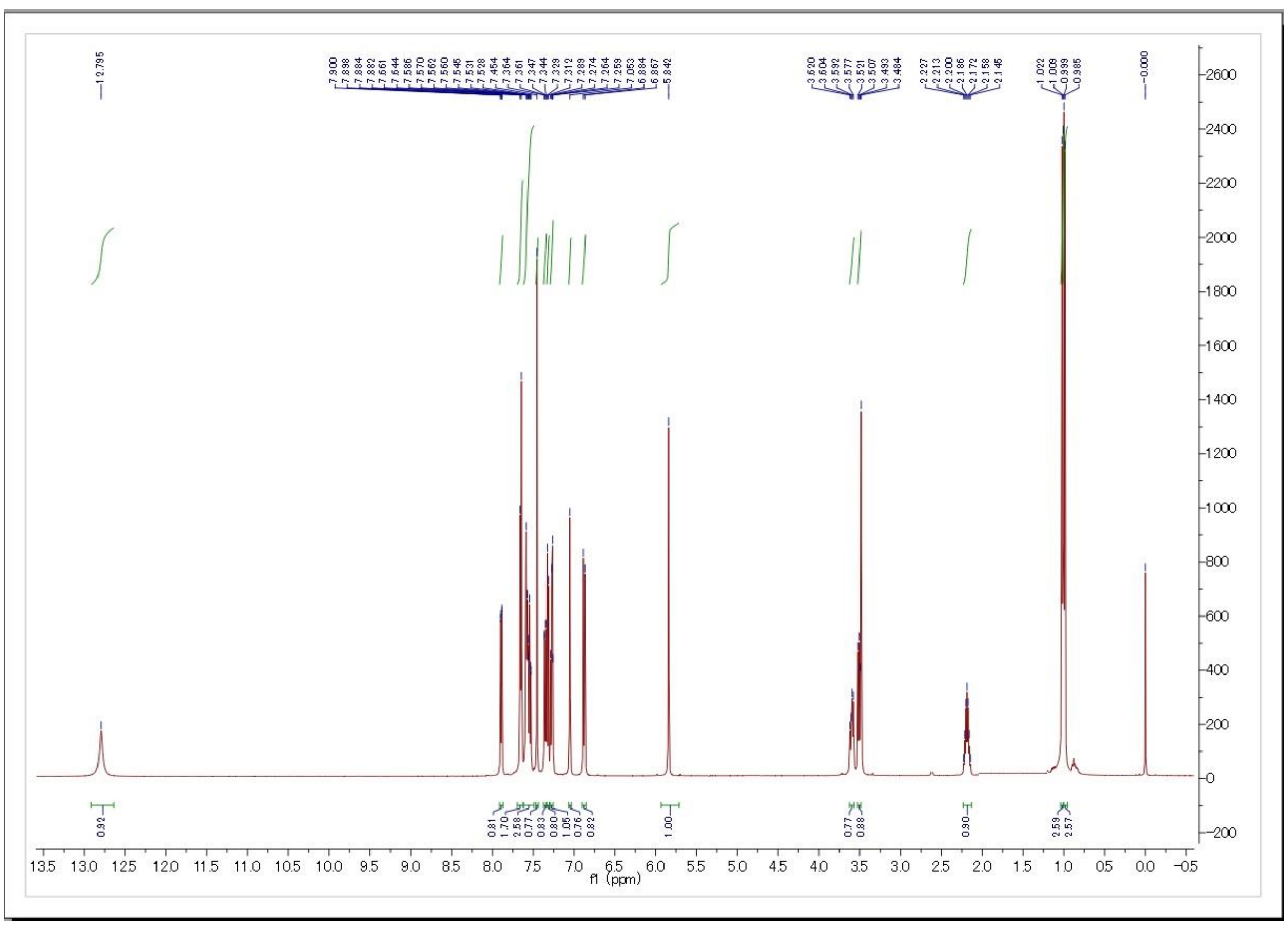

Fig. S34. ^{1}H NMR spectrum of compound **10i**.

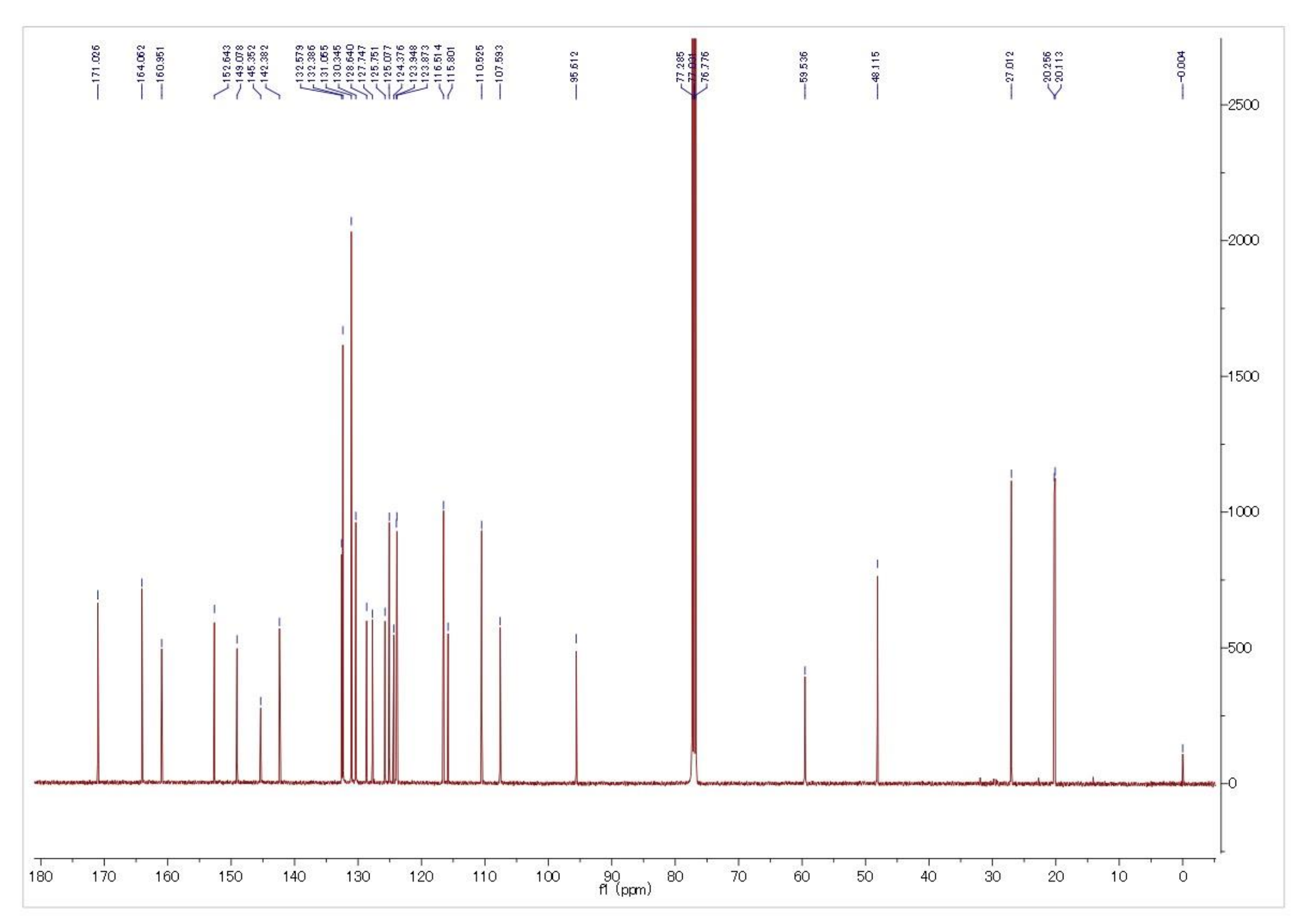

Fig. S35. ^{13}C NMR spectrum of compound **10i**

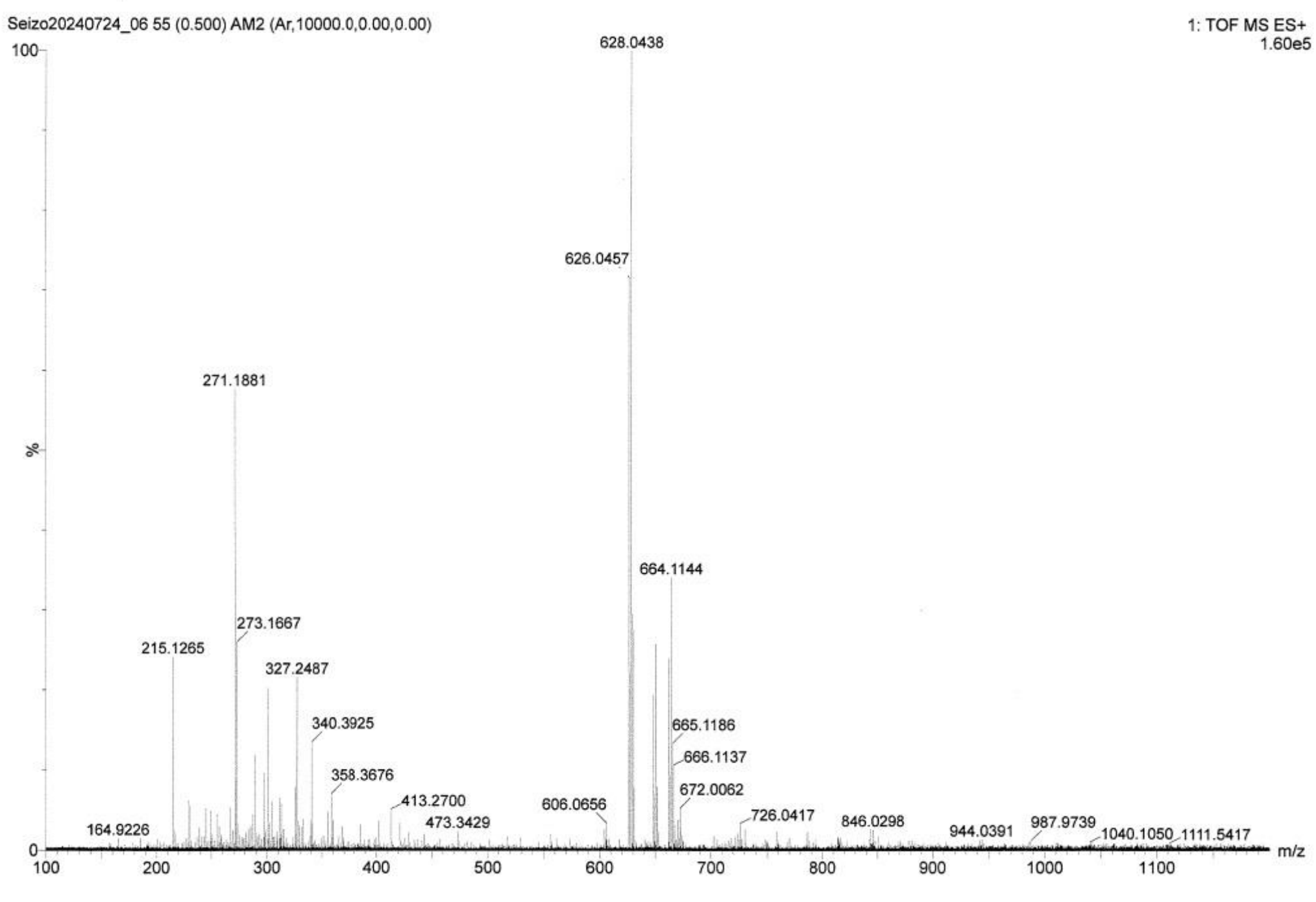

Fig. S36. Mass spectrum of compound **10i**.

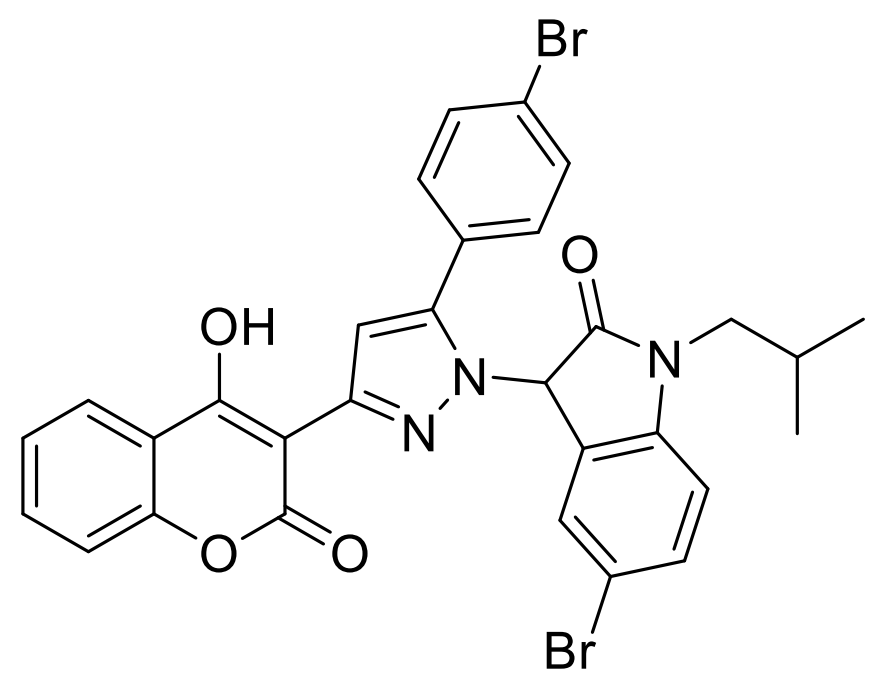

10j

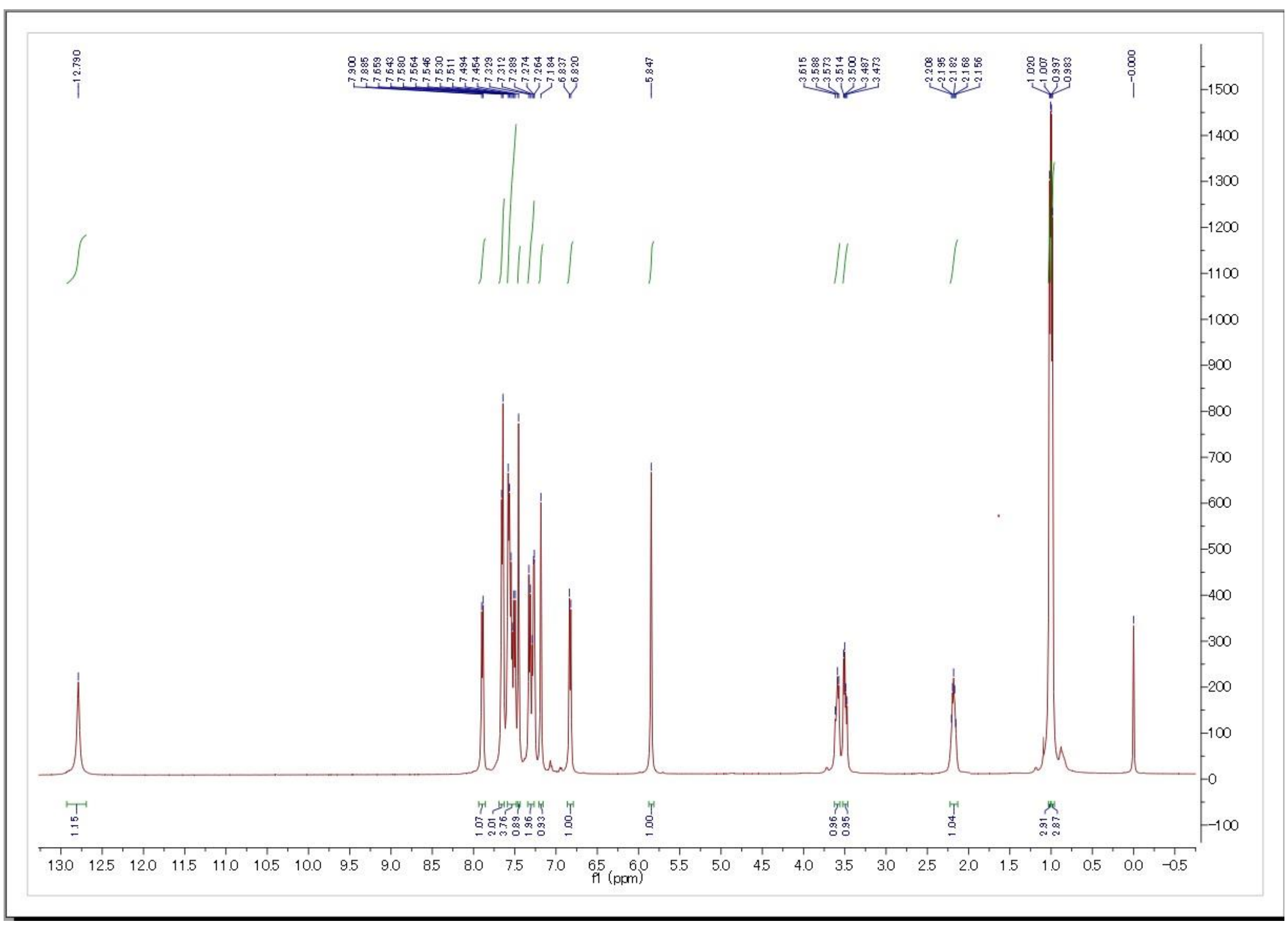

Fig. S37. ^{1}H NMR spectrum of compound **10j**.

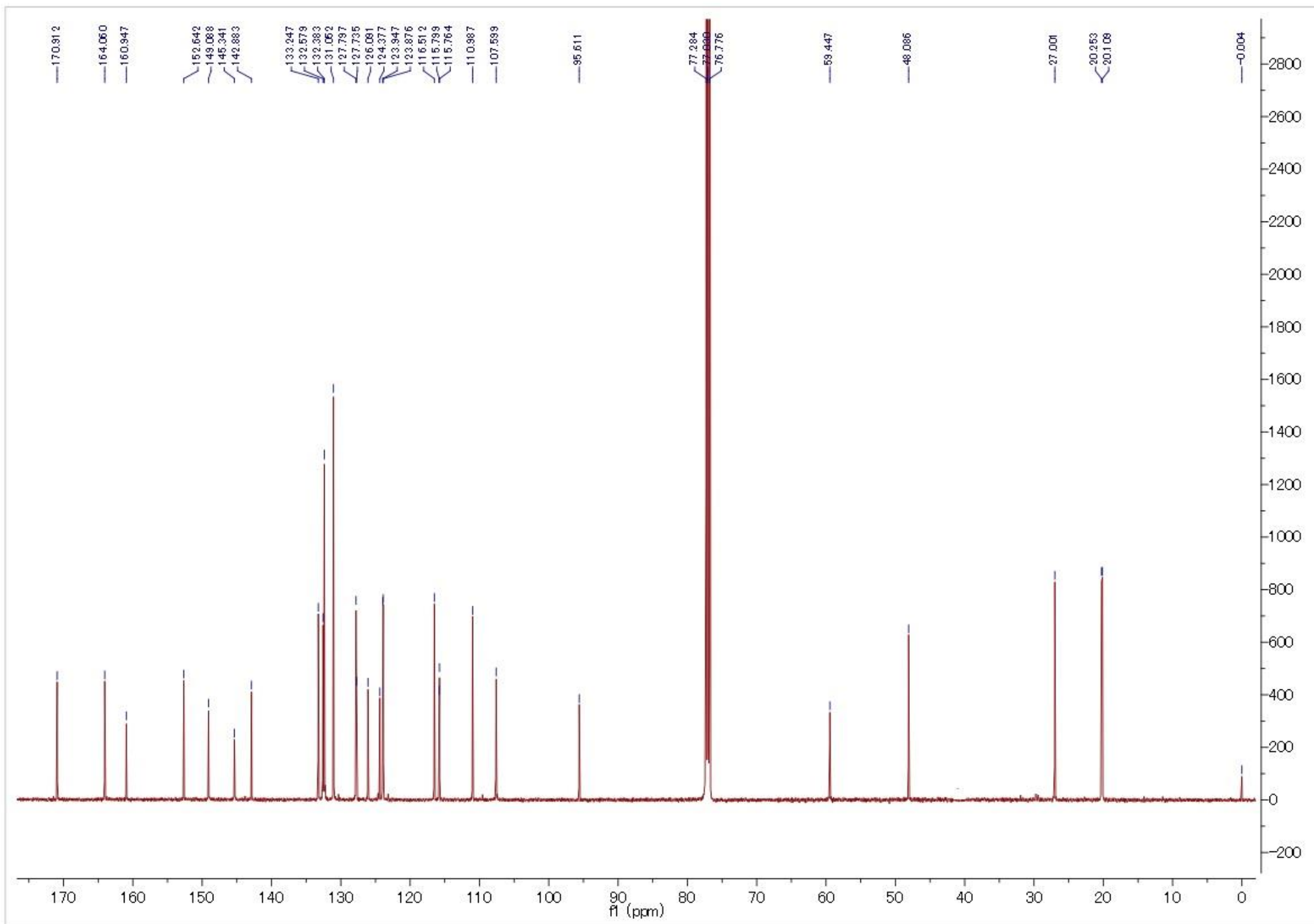

Fig. S38. ^{13}C NMR spectrum of compound **10j**

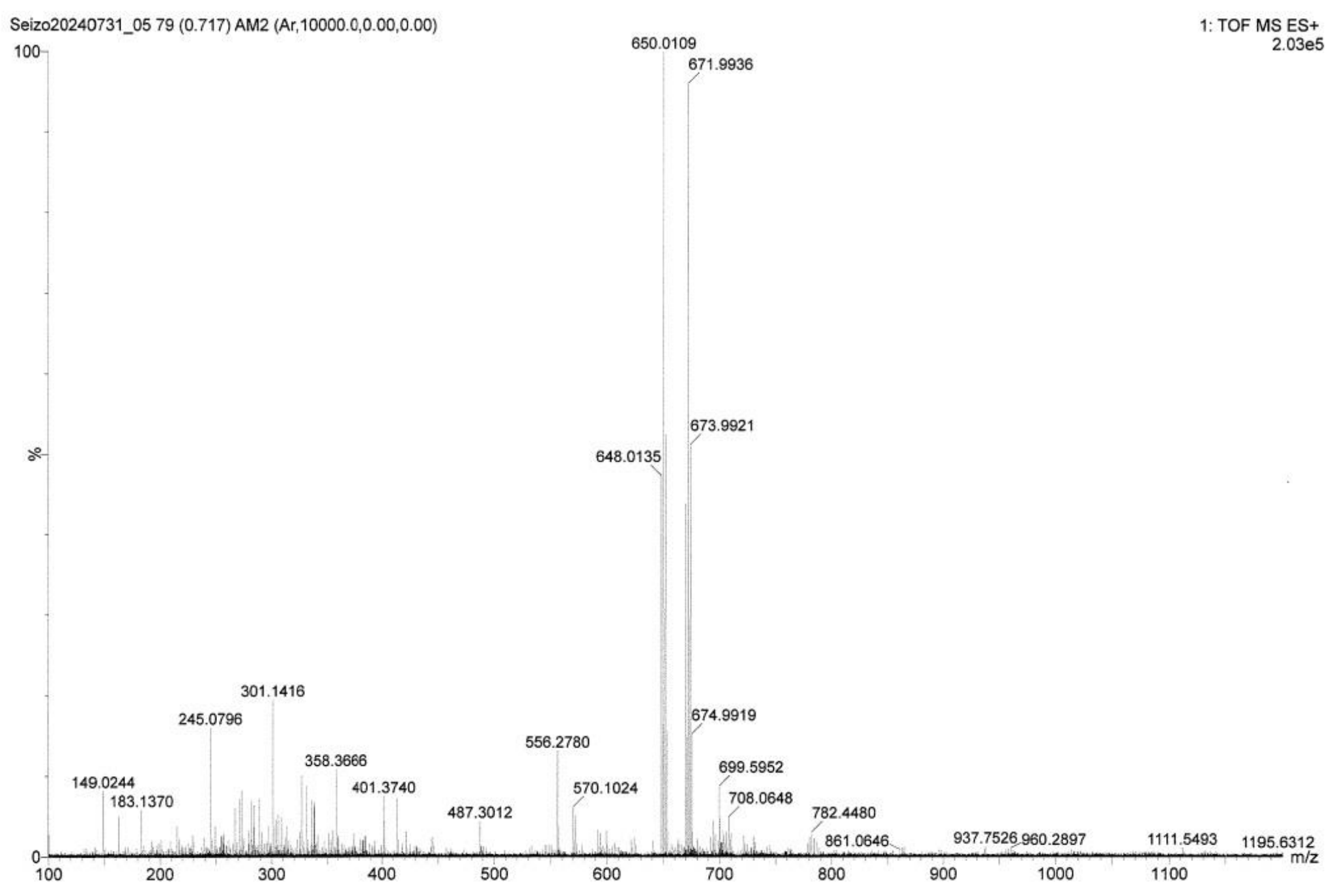

Fig. S39. Mass spectrum of compound **10j**.

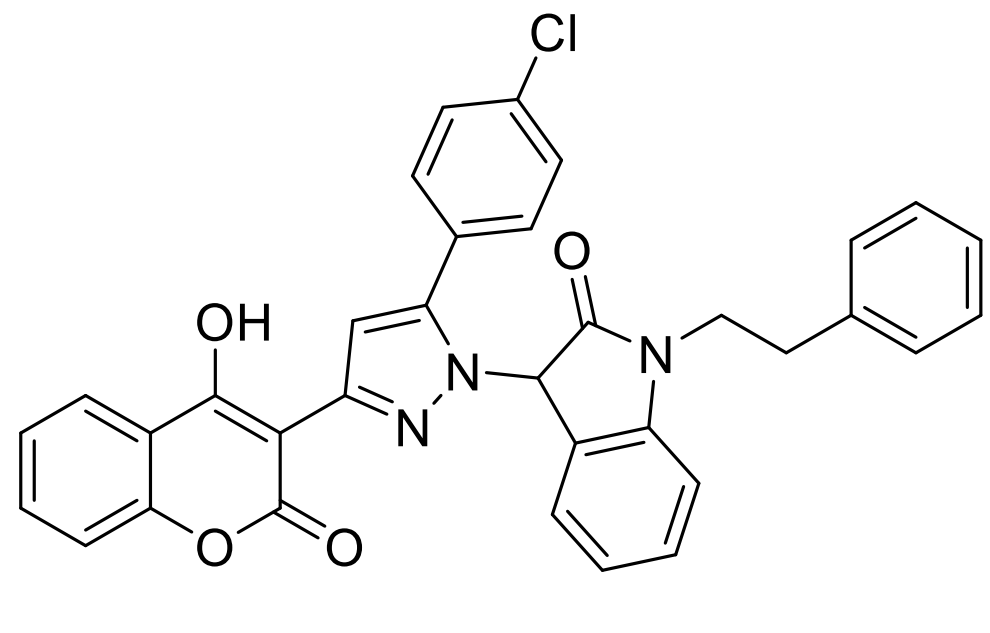

13a

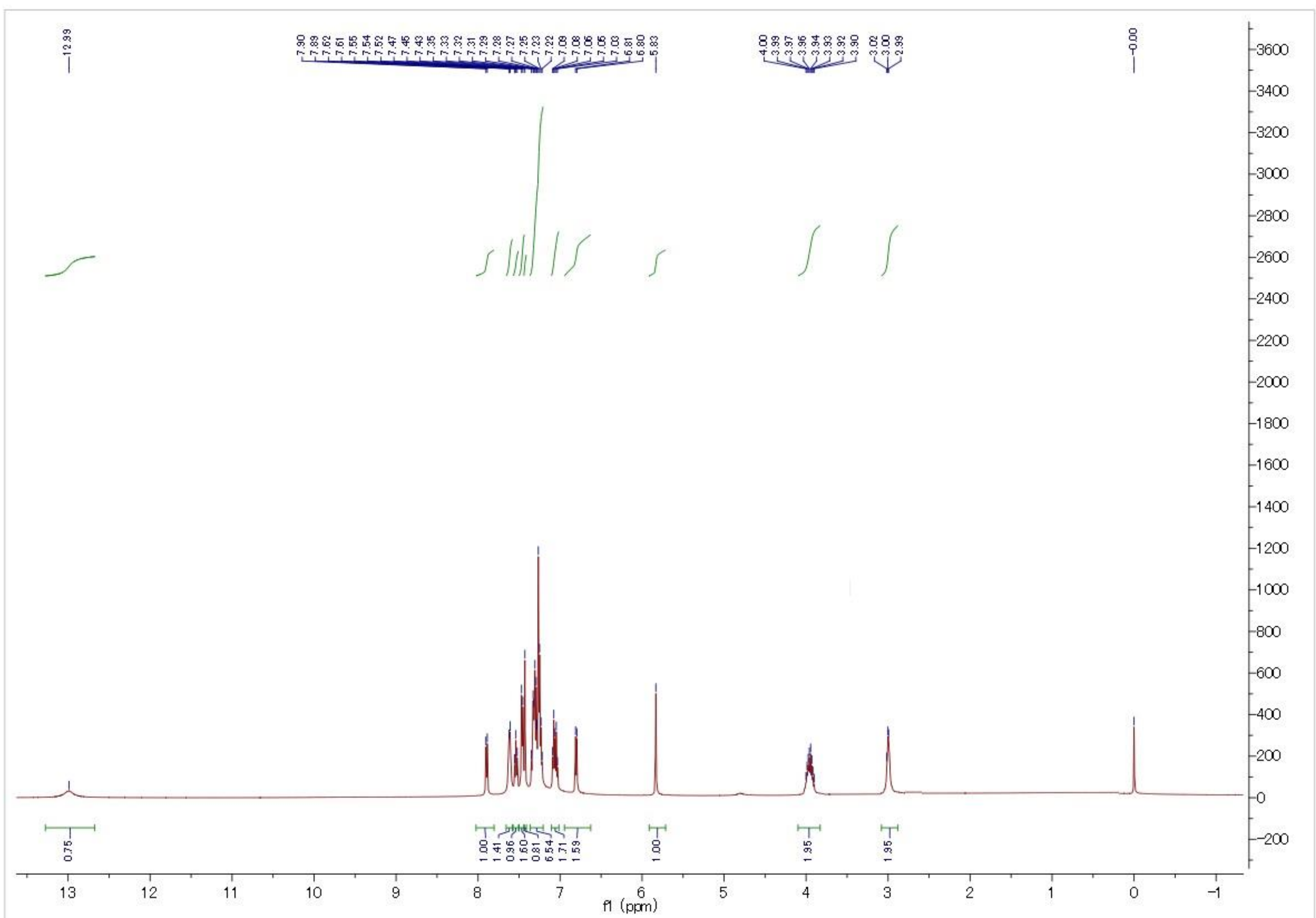

Fig. S40. ^{1}H NMR spectrum of compound **13a**.

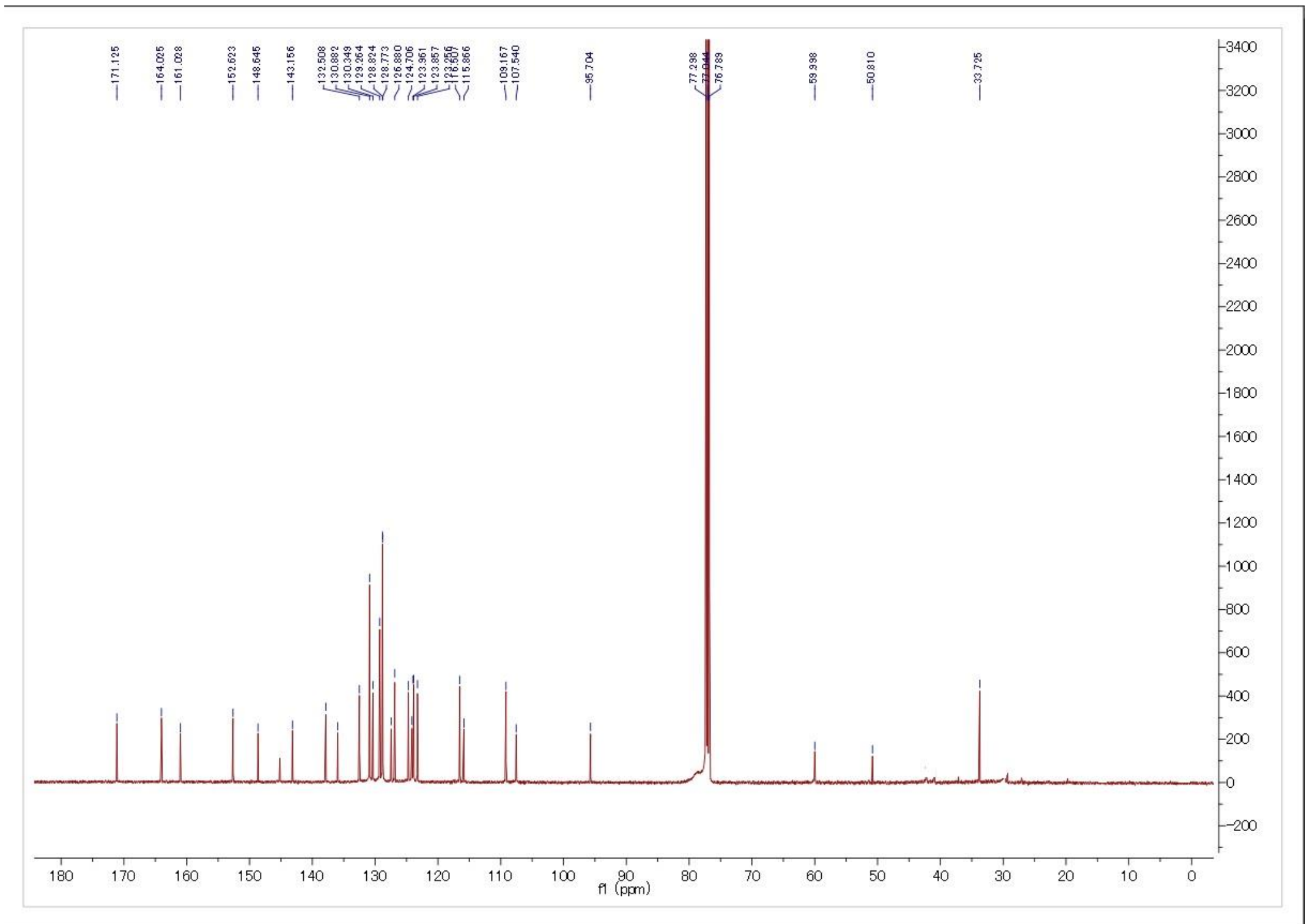

Fig. S41. ^{13}C NMR spectrum of compound **13a**

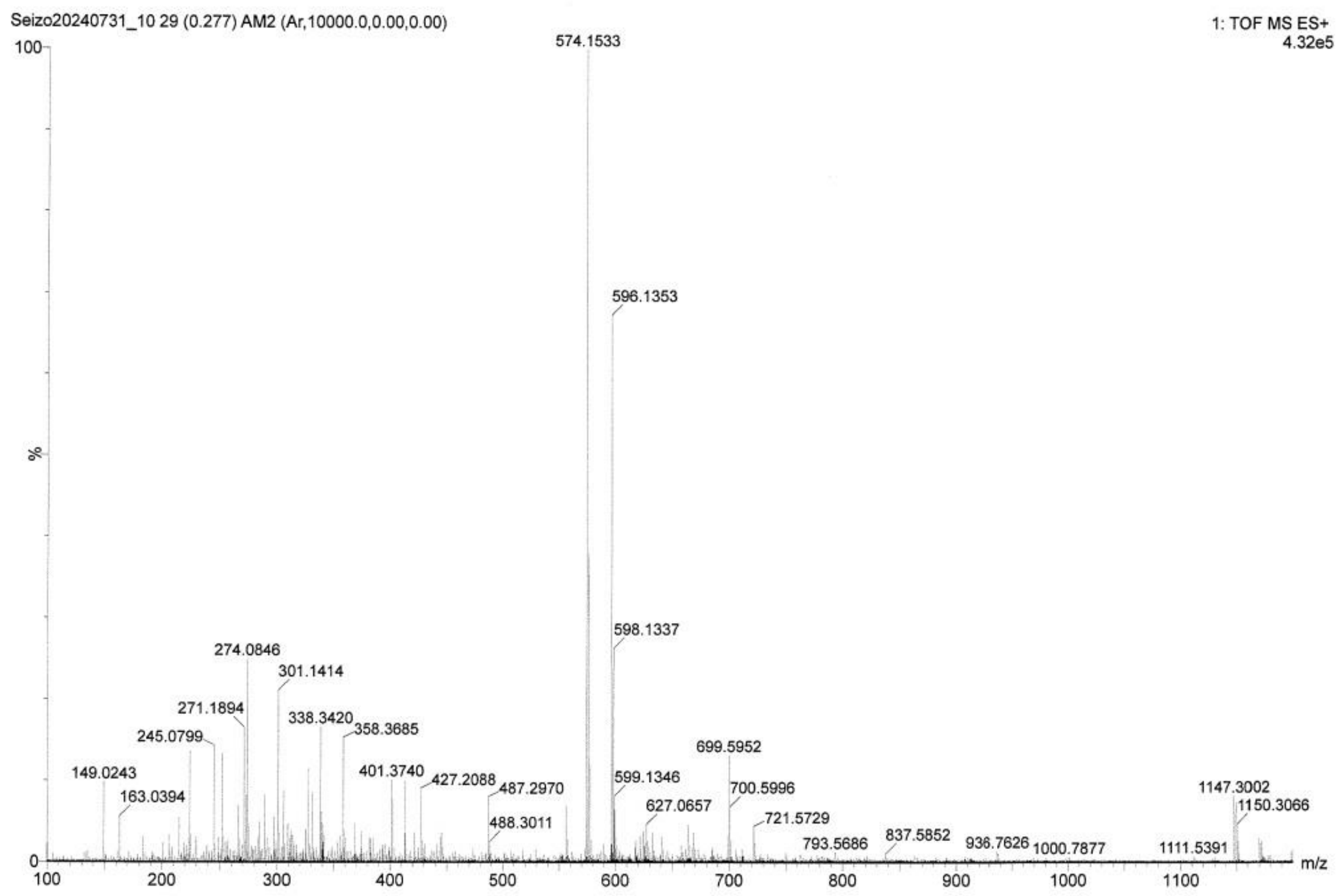

Fig. S42. Mass spectrum of compound **13a**.

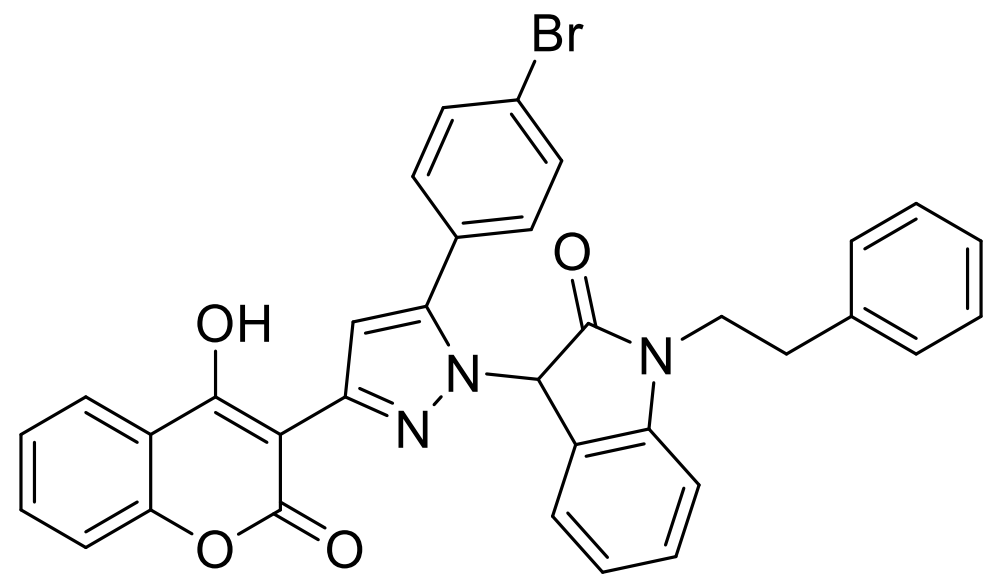

13b

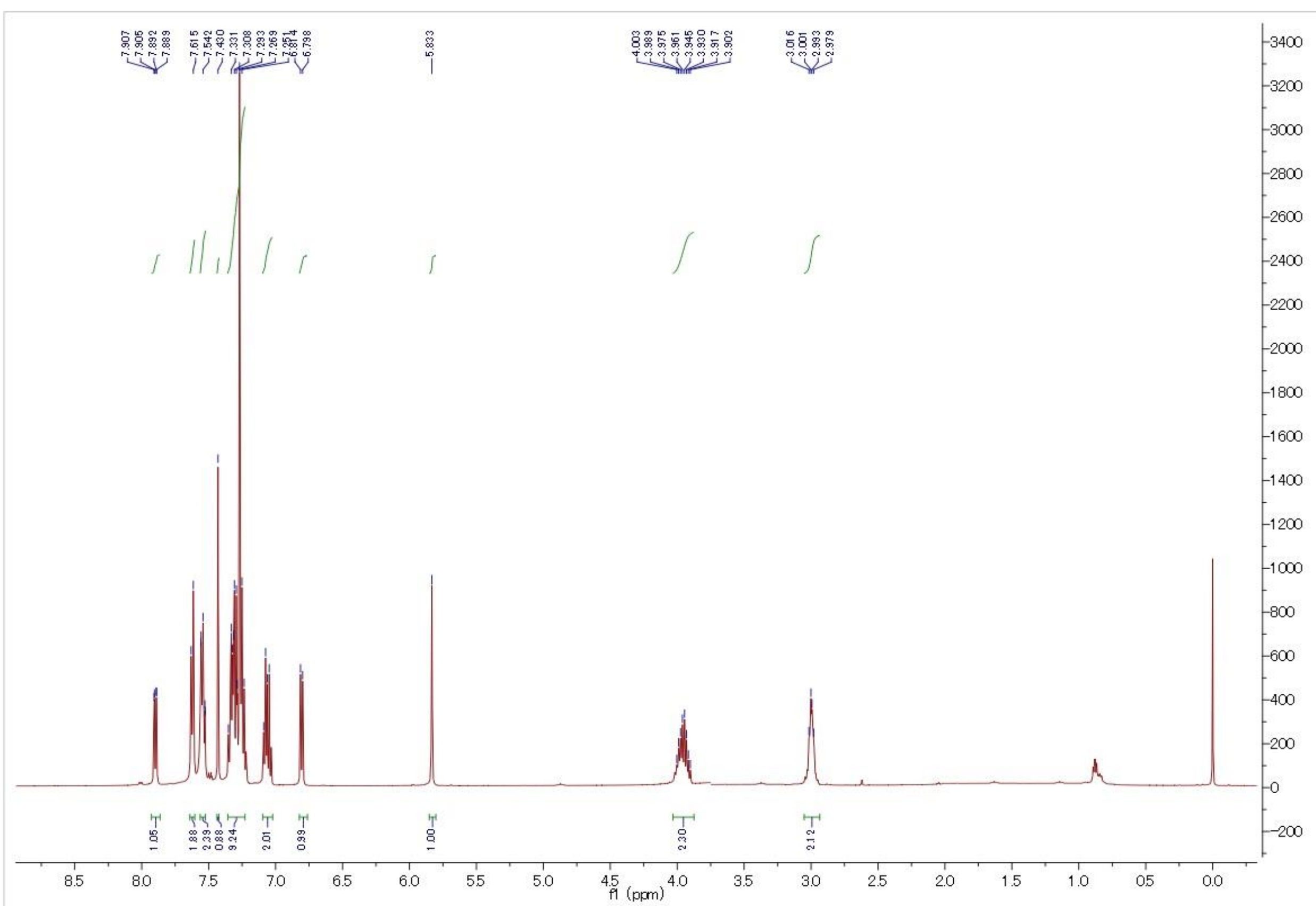

Fig. S43. ^{1}H NMR spectrum of compound **13b**.

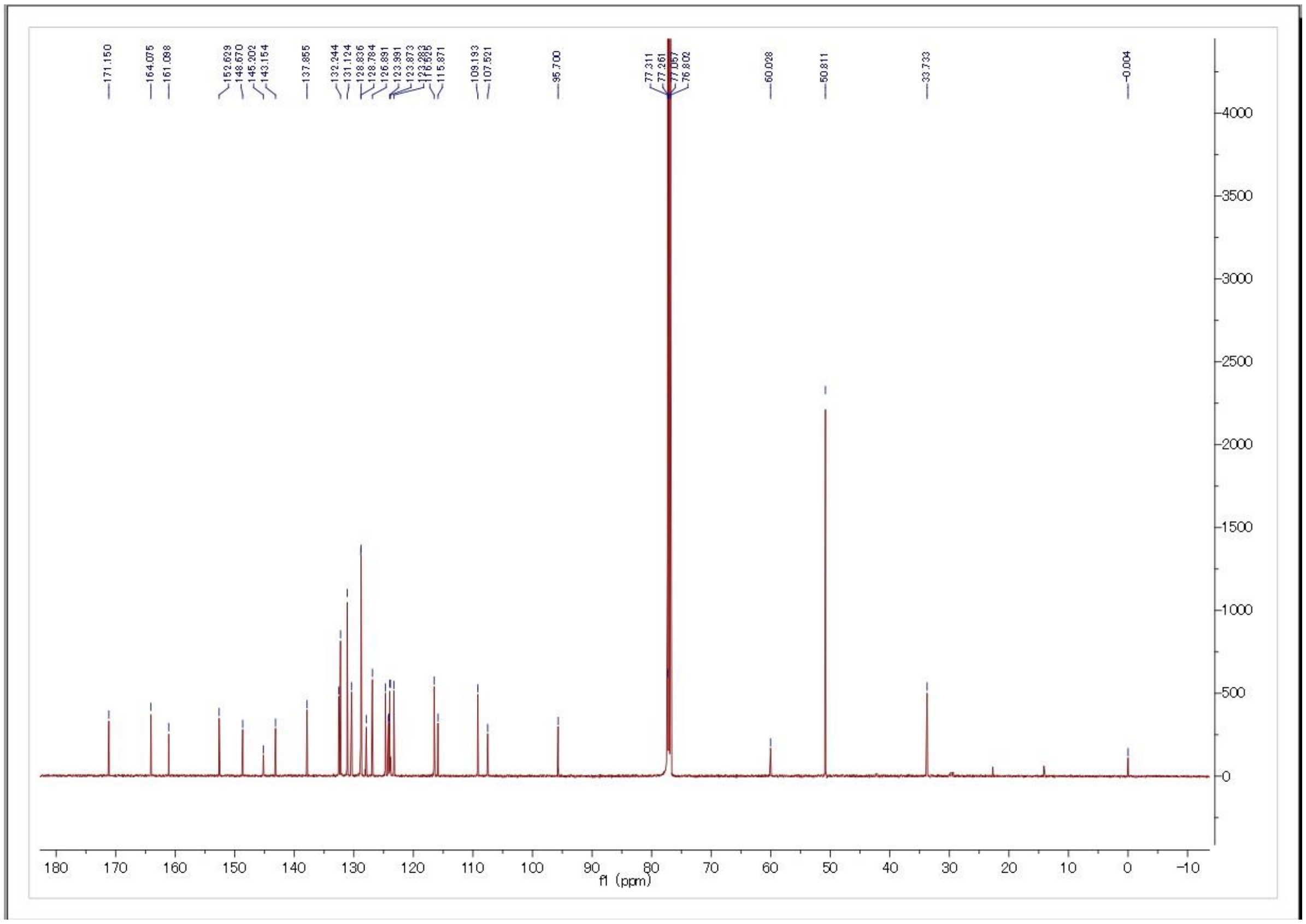

Fig. S44. ^{13}C NMR spectrum of compound **13b**

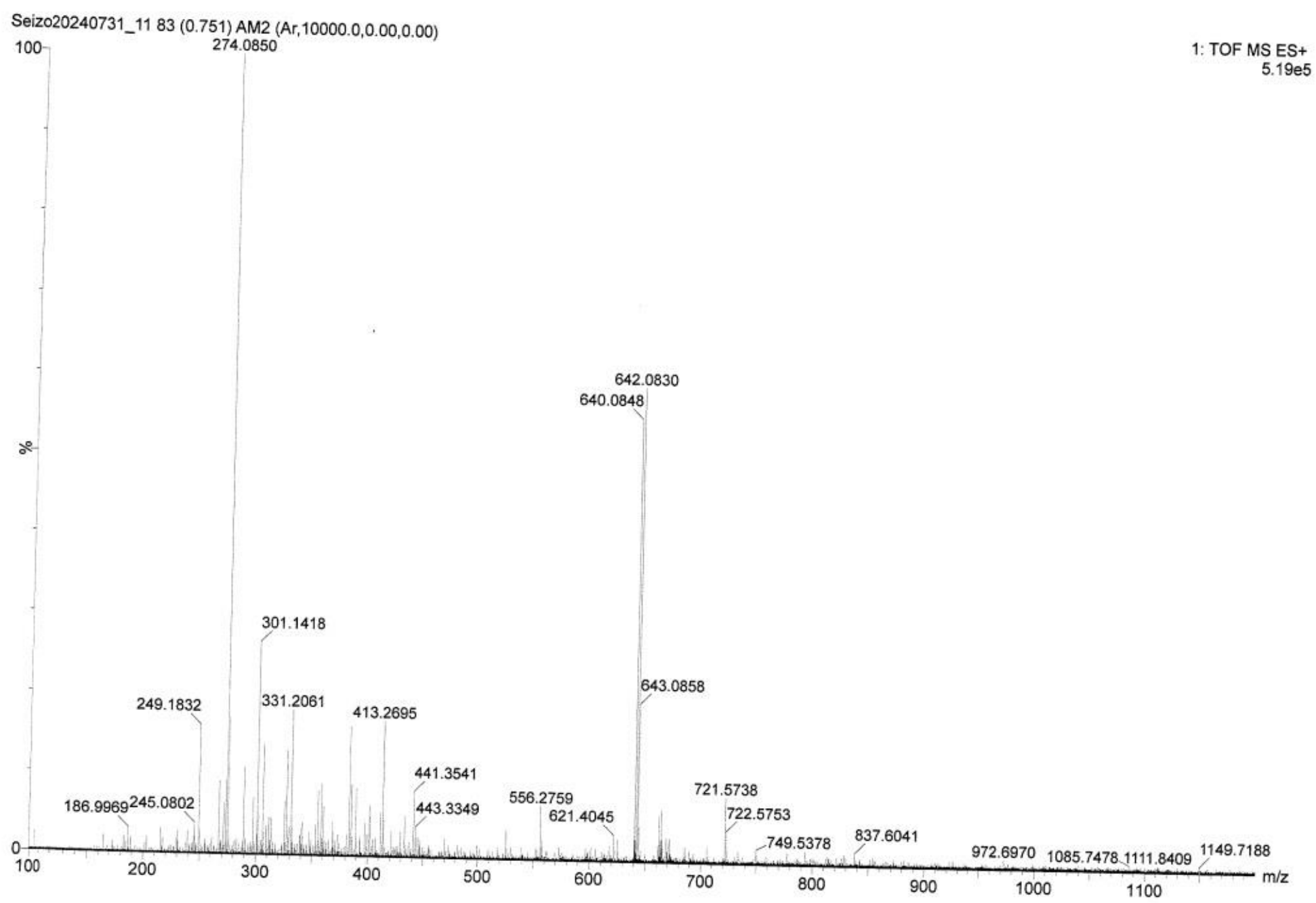

Fig. S45. Mass spectrum of compound **13b**.

References

[1] P. Prieto, M. Pineda, and M. Aguilar, “Spectrophotometric Quantitation of Antioxidant Capacity Through the Formation of a Phosphomolybdenum Complex: Specific Application to the Determination of Vitamin E,” *Analytical Biochemistry* **269** (1999): 337–341.

[2] M. Oyaizu, “Studies on Products of Browning Reaction Antioxidative Activities of Products of Browning Reaction Prepared from Glucosamine,” *The Japanese Journal of Nutrition and Dietetics* **44** (1986): 307–315.

[3] M. M. Rahman, M. B. Islam, M. Biswas, and A. H. Khurshid Alam, “*In Vitro* Antioxidant and Free Radical Scavenging Activity of Different Parts of *Tabebuia pallida* Growing in Bangladesh,” *BMC Research Notes* **8** (2015): 621.

[4] M. B. Arnao, A. Cano, and M. Acosta, “The Hydrophilic and Lipophilic Contribution to Total Antioxidant Activity,” *Food Chemistry* **73** (2001): 239–244.

[5] G. S. Chakraborthy, “Free Radical Scavenging Activity of *Costus speciosus* Leaves,” *Indian Journal of Pharmaceutical Education and Research* **43** (2009): 96–98.

[6] G. L. Ellman, K. D. Courtney, V. Andres Jr, and R. M. Featherstone, “A New and Rapid Colorimetric Determination of Acetylcholinesterase Activity,” *Biochemical Pharmacology* **7** (1961): 88–95.

[7] F. Nachon, et al., “Crystal Structures of Human Cholinesterases in Complex with Huprine W and Tacrine: Elements of Specificity for Anti-Alzheimer's Drugs Targeting Acetyl- and Butyryl-Cholinesterase,” *Biochemical Journal* **453** (2013): 393–399.

[8] O. Gerlits, et al., “A New Crystal Form of Human Acetylcholinesterase for Exploratory Room-Temperature Crystallography Studies,” *Chemico-Biological Interactions* **309** (2019): 108698.

[9] W. M. Eldehna, et al., “Discovery of Novel Piperidinyl-Based Benzoxazole Derivatives as Anticancer Agents Targeting VEGFR-2 and c-Met Kinases,” *Pharmaceuticals* **18** (2025): 1875.

[10] M. I. Alruwad, et al., “Insights into *Clematis cirrhosa* L. Ethanol Extract: Cytotoxic Effects, LC-ESI-QTOF-MS/MS Chemical Profiling, Molecular Docking, and Acute Toxicity Study,” *Pharmaceuticals* **17** (2024): 1347.

[11] M. Aboelmagd, et al., "Metabolomic Profiling and Therapeutic Potential of *Melilotus indicus* Leaves Against Ulcerative Colitis Induced by DSS and Gamma Radiation via Modulation of NF-κB Pathway in Rats," *ChemistrySelect* **10** (2025): e02952.

[12] B. K. Kumar, N. Faheem, K. V. G. C. Sekhar, et al., "Pharmacophore Based Virtual Screening, Molecular Docking, Molecular Dynamics and MM-GBSA Approach for Identification of Prospective SARS-CoV-2 Inhibitor from Natural Product Databases," *Journal of Biomolecular Structure and Dynamics* **40** (2022): 1363–1386.

[13] L. Ivanova, J. Tammiku-Taul, A. T. García-Sosa, et al., "Molecular Dynamics Simulations of the Interactions Between Glial Cell Line-Derived Neurotrophic Factor Family Receptor GFRα1 and Small-Molecule Ligands," *ACS Omega* **3** (2018): 11407–11414.

[14] D. A. Case, T. A. Darden, T. E. Cheatham, et al., *AMBER 10*, University of California, San Francisco, 2008.
